# Ambient Confined-Space Annealing for Crystallization Enhancement and Defect Passivation in Sb_2_S_3_ Thin-Film Solar Cells

**DOI:** 10.1007/s40820-026-02193-w

**Published:** 2026-05-08

**Authors:** Li-Mei Lin, Jie Huang, Hu Li, Jin-Rui Cai, Shui-Yuan Chen, Jian-Min Li, Xiao-Min Wang, Gui-Lin Chen

**Affiliations:** 1 College of Physics and Energy, Fujian Normal University, Fuzhou, 350117 People’s Republic of China; 2 Fujian Provincial Engineering Technology Research Center of Solar Energy Conversion and Energy Storage, Fujian Normal University, Fuzhou, 350117 People’s Republic of China; 3 Key Laboratory of Artificial Micro and Nano-Structures of Ministry of Education, School of Physics and Technology, Wuhan University, Wuhan, 430072 People’s Republic of China; 4 Hubei Key Laboratory of Plasma Chemistry and Advanced Materials, School of Materials Science and Engineering, Wuhan Institute of Technology, Wuhan, 430205 People’s Republic of China

**Keywords:** Sb_2_S_3_, Confined-space annealing, Crystal growth, Passivation, Sb_2_O_3_

## Abstract

Annealing is a crucial step for recrystallizing Sb_2_S_3_ and forming high-quality Sb_4_S_6_ chain-like crystals, which is essential for achieving high-efficiency photovoltaic devices. However, this process currently faces a fundamental trade-off: Although high-temperature annealing enhances crystallinity, it also introduces severe sulfur and Sb_2_S_3_ molecular escape, ultimately degrading device performance. To overcome this limitation, we propose a confined-space annealing (CSA) strategy that operates via a dual mechanism. Physical confinement generates a high local vapor pressure, which suppresses Sb_2_S_3_ re-volatilization and enables recrystallization into large-grain films under atmospheric pressure. Controlled oxygen doping preferentially fills sulfur vacancy sites, suppresses interstitial Sb_i_ defects, and promotes the self-assembly of Sb_2_O_3_ nano-belts at grain boundaries, effectively blocking leakage paths. As a result, the CSA films exhibit a 60.9% reduction in V_S_ defects and a 40.3% improvement in carrier collection efficiency compared to pristine films. Carbon-based devices fabricated using this approach achieve a power conversion efficiency of 7.17% (*V*_OC_ = 750 mV, *J*_SC_ = 14.26 mA cm^−2^, FF = 62.7%), which is the highest reported value for Sb_2_S_3_ solar cells fabricated entirely in ambient atmosphere. This work not only offers a practical fabrication route under ambient conditions but also provides fundamental insights into defect passivation in chalcogenide photovoltaics.

**Supplementary Information:**

The online version contains supplementary material available at 10.1007/s40820-026-02193-w.

## Highlights

- Effectively inhibits the re-volatilization of Sb_2_S_3_, allowing recrystallization at 450 °C, and preparing films with large grains.
- The confined-space annealing strategy enables controlled dynamic in situ oxidation targeting defect sites and reduces the defect concentration of the V_S_ by 60.9%.
- It represents the first reported highest efficiency of 7.17% in full air, which is 40.3% higher than the efficiency of Sb_2_S_3_ devices under the N_2_ atmosphere.

**Supplementary Information:**

The online version contains supplementary material available at 10.1007/s40820-026-02193-w.

## Graphical abstract

**Figure:**
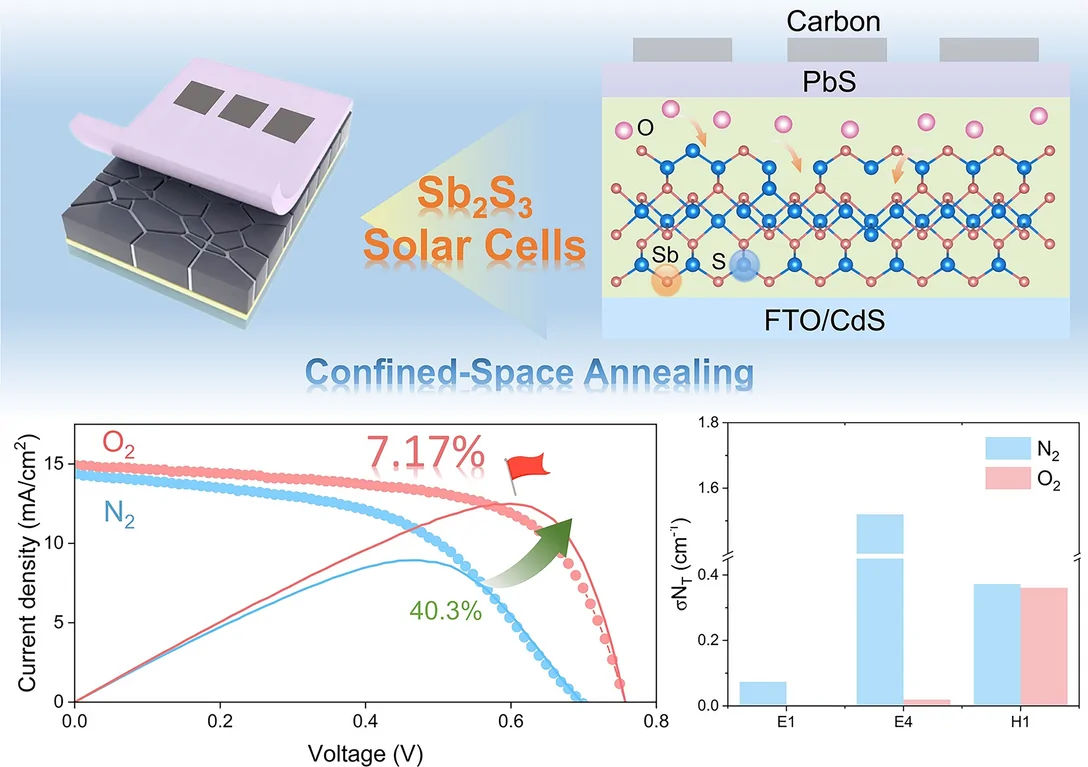

## Introduction

Thin-film solar cells have emerged as a pivotal research direction in photovoltaics, owing to their significant potential for cost reduction and lightweight design, which renders them promising for integration into emerging applications [1–3]. Among various emerging light-absorbing materials, antimony chalcogenides Sb_2_X_3_ (mainly Sb_2_S_3_, Sb_2_Se_3_, and their solid solutions Sb_2_(S,Se)_3_) exhibit notable advantages, including a stable single-phase structure, high absorption coefficients (~ 10^5^ cm^−1^), tunable bandgaps (1.1–1.7 eV), eco-friendly composition, and low crystallization temperature [4–8]. In particular, Sb_2_S_3_, with a bandgap of ~ 1.7 eV, has drawn significant interest, not only as a candidate for single-junction solar cells but also as an ideal top-cell material for tandem configurations [9–12]. These compelling prospects have motivated extensive research, leading to steady improvements in device performance [13–15]. Although recent advances, such as the use of molecular additives and interface modification, have pushed the certified efficiency of Sb_2_S_3_ devices to over 8%, this value remains well below the Shockley–Queisser theoretical limit (~ 28.6%) [9, 14, 16]. Both theoretical and experimental studies identify severe non-radiative recombination within the absorber layer and at interfacial regions as the primary bottleneck limiting further performance improvement [17–19].

Sb_2_S_3_ is a binary compound, its asymmetric crystal structure gives rise to 12 intrinsic point defects, among which sulfur vacancies (V_S_) act as deep-level recombination centers and degrade charge transport. Therefore, effective passivation of these deep-level defects represents a central challenge in enhancing device performance. Annealing is essential for enhancing crystallinity and reducing defects in Sb_2_S_3_ thin films, yet conventional approaches face two difficult trade-offs [20–24]. The first concerns the controllability of oxygen doping. Density functional theory (DFT) calculations suggest that appropriate oxygen incorporation can fill V_S_ sites [16]. Experimentally, Steiner et al. demonstrated that a surface oxide layer formed via low-temperature (200 °C) air annealing suppressed back-interface recombination, raising device efficiency from 1.4% to 2.4% [25]. Similar benefits have been observed in Sb_2_Se_3_ solar cells [26, 27]. However, this oxygen passivation requires overcoming a high energy barrier, making precise doping challenging. Moreover, oxygen incorporation inevitably leads to the formation of poorly conductive Sb_2_O_3_, which severely impedes carrier transport [28]. Thus, balancing “oxygen doping for defect passivation” and “avoiding bulk oxidation” remains an unresolved issue. The second challenge relates to volatilization limits during high-temperature annealing. The primary goal of annealing is to supply energy for the decomposition of Sb_4_S_6_ nano-belts, atomic migration, and recrystallization to promote grain growth and defect reduction. However, under a conventional open-annealing model at high temperatures, sulfur and Sb_2_S_3_ fragments readily volatilize due to their high saturated vapor pressure, restricting practical annealing temperatures to 300–400 °C, far below the 450–600 °C commonly used for other chalcogenides like copper indium gallium selenide (CIGS) and copper zinc tin sulfide selenide (CZTSSe) [15, 16, 29–36]. This upper temperature limits curbs full grain growth and crystallinity improvement, forming a major bottleneck in film quality.

To overcome the above challenges, this study introduces a confined-space annealing (CSA) strategy for post-treatment of the hydrothermally derived precursor films under ambient pressure. The CSA approach simultaneously enables: (1) high-temperature recrystallization at 450 °C by generating a localized high vapor pressure that suppresses Sb_2_S_3_ volatilization, thereby facilitating large-grained growth with reduced defect density; and (2) controlled in situ oxidation that achieves selective sulfur vacancy passivation via oxygen doping, while preventing bulk oxidation through reaction confinement. The resulting all-inorganic Sb_2_S_3_ solar cell with an FTO/CdS/Sb_2_S_3_/PbS/carbon structure achieves a power conversion efficiency (PCE) of 7.17% under fully non-vacuum conditions which delivers a 40.3% improvement over N_2_-annealed devices. This work provides a scalable fabrication route and key insight into synergistic oxygen passivation and high-temperature crystallization in Sb_2_S_3_ photovoltaics.

## Experimental Section

### Materials

The following materials were used in this study: fluorine-doped tin oxide (FTO, SnO_2_:F, Prime Option Co., Ltd), cadmium nitrate tetrahydrate (Cd(NO_3_)_2_·4H_2_O, AR, Sinopharm Chemical Reagent), thiourea (CH_4_N_2_S, AR, Sinopharm), ammonia solution (NH_3_·H_2_O, 25%–28%, Sinopharm), cadmium chloride hemipentahydrate (CdCl_2_·2.5H_2_O, AR, Sinopharm), Methanol (CH_3_OH, AR, Sinopharm), antimony potassium tartrate hemihydrate (KSbC_4_H_4_O_7_·1/2H_2_O, AR, Macklin), sodium thiosulfate pentahydrate (Na_2_S_2_O_3_·5H_2_O, AR, Sinopharm), lead(II) acetate trihydrate (Pb(CH_3_COO)_2_·3H_2_O, AR, Macklin), carbon electrode paste (C, 99%, Shanghai MaterWin New Materials Co., Ltd), and silver paste (Ag, SPI Supplies). All chemicals were used as received without additional purification.

### Device Fabrication

First, a CdS thin film was deposited on commercial FTO substrates via chemical bath deposition (CBD) at 65 °C for 15 min. The resulting CdS layer was then treated with a 5 mg mL^−1^ CdCl_2_ methanol solution and annealed at 380 °C for 5 min to enhance its crystallinity. Subsequently, an Sb_2_S_3_ precursor film was deposited onto the FTO/CdS substrate via a hydrothermal method at 120 °C for 10 h. The hydrothermal deposition time was varied (5, 10, and 15 h) to control the precursor thickness, and 10 h was determined as the optimal condition for achieving high-quality films under CSA (Fig. S1), as evidenced by the device performance statistics shown in Fig. S2. Detailed procedures for the CdS and Sb_2_S_3_ deposition are described in our previous work [37].

Next, the FTO/CdS substrate with the Sb_2_S_3_ precursor film deposited on it was inverted and placed on a clean soda-lime glass (SLG) to form a sandwich structure. The surface of the Sb_2_S_3_ film naturally came into contact with the SLG, and the contact gap was measured by an optical microscope to be 4.6 ± 0.5 μm. (Fig. S3 presents the results of multiple measurements.) The Sb_2_S_3_ precursor films were air-annealed on a hot plate at different temperatures for 3 min. After annealing, to prevent the FTO substrate from cracking due to excessive temperature difference, the samples were moved to a low-temperature zone at 250 °C for natural cooling. During the cooling process, the FTO substrate was quickly removed at different temperature to expose the Sb_2_S_3_ film to the ambient air for in situ oxidation. Control films were annealed in a nitrogen atmosphere within a glove box. Following this, a PbS film was deposited onto the annealed Sb_2_S_3_ layer via a hydrothermal method: Briefly, the Sb_2_S_3_ film was immersed in a mixture of 35 mL of 0.8 mM Pb(CH_3_COO)_2_·3H_2_O and 35 mL of 1.6 mM Na_2_S_2_O_3_·5H_2_O, and heated at 120 °C for 35 min. Energy level arrangement diagram is shown in Fig. S4. The PbS layer, serving as the hole transport layer, has a stepwise energy level alignment with the Sb_2_S_3_, which is conducive to hole extraction. This well-matched band structure promotes favorable charge carrier transport and reduces interfacial recombination, thereby contributing to the enhanced performance of the device [38]. Finally, carbon paste and silver paste were sequentially brush-coated as electrode materials to complete the device fabrication. The active area of the solar cells was defined as 0.09 cm^2^ using a shadow mask.

To facilitate the distinction of different annealing conditions, the following naming convention was adopted in this work: The prefixes “O,” “C,” “U,” “D,” and “N,” respectively, represent open-air annealing, confined-space annealing, up-coverage annealing, dynamic in situ oxidation, and nitrogen atmosphere annealing. The detailed conditions of all samples are summarized in Table S5.

### Characterization

Film morphology and composition were characterized by field-emission scanning electron microscopy (FESEM, Hitachi SU-8010) equipped with an energy-dispersive X-ray spectrometer (EDS). Structural properties of the films were investigated by X-ray diffraction (XRD, Rigaku Ultima IV, Cu Kα radiation λ = 1.5406 Å). Photoluminescence (PL) spectra were acquired using a Raman spectrometer (Horiba LABRAM-HR) with 532 nm laser excitation. The electrical conductivity of Sb_2_S_3_ films was measured via conductive atomic force microscopy (c-AFM, Bruker Dimension Icon). Surface composition was analyzed by X-ray photoelectron spectroscopy (XPS, Thermo Fisher Escalab 250XI). Optical properties were evaluated by measuring transmittance using an ultraviolet–visible–near-infrared spectrophotometer (PerkinElmer Lambda 950). The current density–voltage (*J*-*V*) characteristics of the solar cells were measured under AM 1.5 G illumination (100 mW cm^−2^) using a solar simulator (San-Ei Electric XES-40S1) and a semiconductor parameter analyzer (Keithley 2400). The scanning range is from − 0.2 to 0.8 V, with a forward scan. The data points are 100 (with a voltage step size of approximately 0.01 V), the waiting time is 0.5 s, the effective scanning rate is 0.02 V s^−1^, the current range is 0.2 A, and the current compensation is − 0.2 A. No light soaking or voltage pre-treatment was performed before the *J*-*V* test. The effective area of the device was precisely limited by a metal mask to 0.09 cm^2^. Light intensity was calibrated using a reference cell and filters to match the measured short-circuit current density (*J*_SC_) and open-circuit voltage (*V*_OC_) under standard conditions. Dark *J*-*V* curves were obtained using a Keithley 4200-SCS parameter analyzer. Capacitance–voltage (*C*-*V*) measurements were performed in the dark at room temperature using the Keithley 4200-SCS system, over a frequency range of 1 to 50 kHz with an AC amplitude of 30 mV. External quantum efficiency (EQE) spectra were acquired using a quantum efficiency measurement system (PV Measurements QEX10). Electrochemical impedance spectroscopy (EIS) was conducted in the dark using an electrochemical workstation (Zahner Zennium) across a frequency range of 10 Hz to 1 MHz. Transient photovoltage (TPV) and transient photocurrent (TPC) measurements were carried out using a system comprising a 530 nm monochromatic light source, a function generator, and an oscilloscope. Deep-level transient spectroscopy (DLTS) characterization for defect analysis was performed using a Phystech FT-1230 HERA DLTS system, with temperature scans from 120 to 425 K in 2 K steps. Additional EIS measurements were performed using a Chi760e electrochemical workstation under a 0.50 V bias in the dark, spanning a frequency range from 1 to 1 MHz.

## Results and Discussion

### Confined-Space Annealing for High-Temperature Processing

**Fig. 1:**
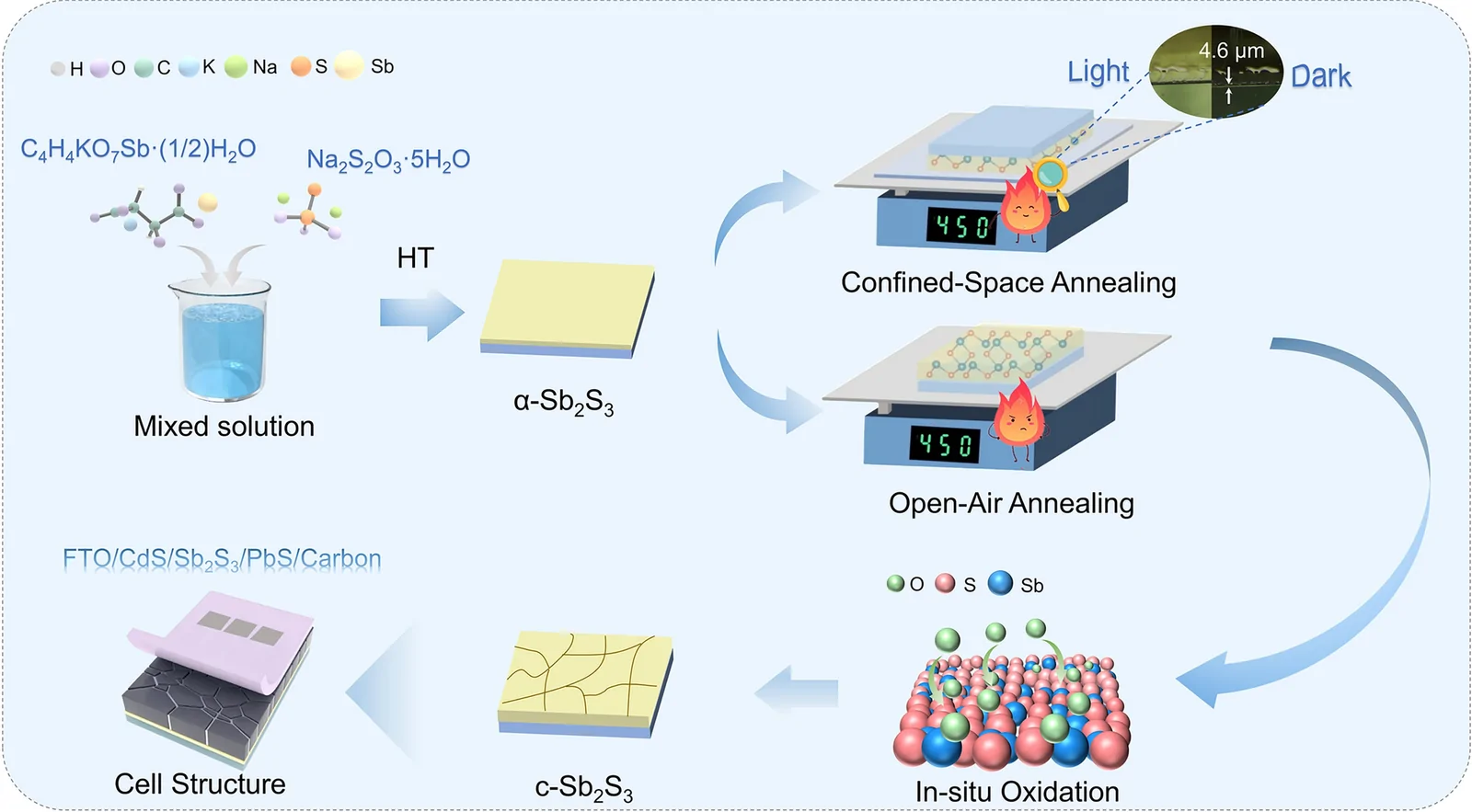
Schematic diagram of the open-air annealing or confined-space annealing FTO/CdS/Sb_2_S_3_ preparation process

As an essential step for driving solid-state recrystallization of Sb_2_S_3_ precursor, annealing enables the decomposition of Sb_4_S_6_ nano-belts, atomic migration, and grain growth at temperatures above 350 °C, which are crucial for forming large-grained films with low defect density. As shown in the device fabrication process of Fig. 1, the hydrothermal-derived amorphous Sb_2_S_3_ (denoted as α-Sb_2_S_3_) experiences two distinguished annealing models, namely conventional open-air annealing (OAA) and newly designed confined-space annealing (CSA), to promote the recrystallization of Sb_2_S_3_ (denoted as c-Sb_2_S_3_). With the open model in OAA, the sharp increase in saturated vapor pressure of Sb_2_S_3_ (e.g., based on Note S1, approximately increases from 9.1 Pa at 350 °C to 194.8 Pa at 450 °C, as shown in Fig. S5) [39], the progressive weakening of Sb-S bonds at elevated temperatures [40], and the structural instability arising from the partially unsaturated coordination of sulfur in the orthorhombic (Pnma) lattice together (Fig. S6) lead to severe component re-evaporation and sulfur vacancy (V_S_) formation. Herein, we designed this CSA strategy under an ambient atmosphere, whose configuration and key dimensions are illustrated in Fig. 1. The approach is based on a sandwich structure in which the α-Sb_2_S_3_ films are inversely placed over a SLG, forming a microscale confined gap (4.6 ± 0.5 μm). As shown in the inset optical micrographs of Fig. 1, the confined configuration was characterized by dark-field (left) and bright-field (right) imaging to verify the gap uniformity and contact state. The elliptical region highlights a representative local confined gap, where forms a stable submicron air interlayer. This geometry significantly increases the local vapor pressure of Sb_2_S_3_, thereby suppressing the evaporation of volatile species and permitting high-temperature annealing.

**Fig. 2:**
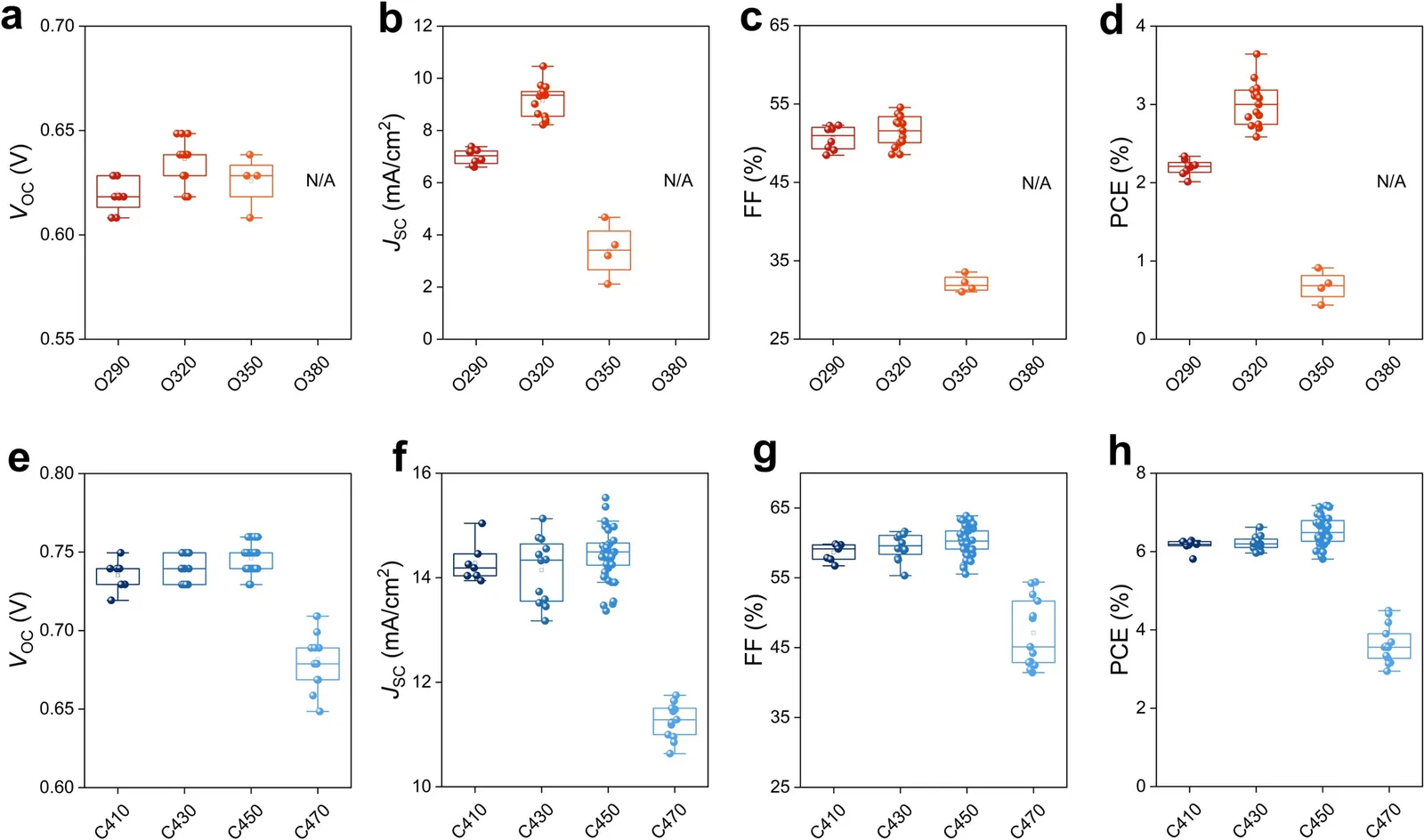
Device Performance. The performance parameters of Sb_2_S_3_ devices annealed with **a-d** the OAA and **e–h** the CSA at different temperatures

The *J-V* statistics of the solar cells (Fig. 2a-d) reveal a clear temperature-dependent trend under OAA conditions (samples O290-O380). As the annealing temperature increases from 290 to 320 °C, the short-circuit current density (*J*_SC_) significantly rises from an average of 6.99 to 9.16 mA cm^−2^, while the open-circuit voltage (*V*_OC_) and fill factor (FF) also improve slightly (from 620 to 636 mV and from 50.7% to 51.5%, respectively). This enhancement is attributed to improved crystallinity and reduced lattice defects at elevated temperatures, raising the average PCE from 2.19 to 3.08%. The best OAA device (O320) delivered *V*_OC_ = 638 mV, *J*_SC_ = 10.46 mA cm^−2^, FF = 54.6%, and PCE = 3.64%. However, when the temperature reaches 350 °C (O350), all device parameters degrade sharply (*V*_OC_ = 625 mV, *J*_SC_ = 3.40 mA cm^−2^, FF = 32.1%, and PCE = 0.67%), and the 380 °C-annealed sample (O380) loses its photovoltaic response entirely. This indicates that although OAA can moderately improve performance at ≤ 320 °C, its usable temperature window is narrow and incompatible with the high-temperature recrystallization required for high-quality films.

In stark contrast, the CSA strategy circumvents this temperature-induced limitation. As shown in Fig. 2e-h and Table S2, devices annealed via CSA (labeled C290-C470) exhibit markedly superior and more stable performance, especially in the 410–450 °C range (see also the lower temperature of 290–380 °C in Fig. S7). The C450-CSA devices deliver average photovoltaic parameters of 6.52% PCE, 746 mV *V*_OC_, 14.45 mA cm^−2^ *J*_SC_, and 60.2% FF, significantly surpassing the OAA result. To further optimize the process conditions of the CSA method, we systematically investigated the influence of annealing time (2, 3, and 4 min) on the morphology evolution of Sb_2_S_3_ thin films at 450 °C and the subsequent device performance (Figs. S8 and S9). As shown in Fig. S8, the 2 min annealed film exhibits fine Sb_2_O_3_ particles on the surface and a relatively loose cross‑sectional structure, indicating insufficient grain growth. After 3 min annealing, the cross section reveals a dense and continuous morphology with well‑developed grains. However, extending the annealing time to 4 min leads to excessive growth of Sb_2_O_3_ particles, blurred grain boundaries, and the appearance of voids in the cross section, suggesting over‑oxidation and structural degradation. In combination with the device performance statistics (Fig. S9), the devices fabricated under the 3 min annealing condition exhibited the highest PCE, confirming that 3 min is the optimal annealing time for the CSA strategy. All subsequent experiments were conducted under this condition. A similar volatile-suppression effect was confirmed using a simplified “up-coverage” configuration (covering the Sb_2_S_3_ surface with SLG, Fig. S10). Devices in this mode (U410-U470, Fig. S11) also showed significantly enhanced performance, with an average PCE of 6.21% (*V*_OC_ = 727 mV, *J*_SC_ = 14.1 mA cm^−2^, FF = 60.5%), far higher than the counterpart of OAA device.

**Fig. 3:**
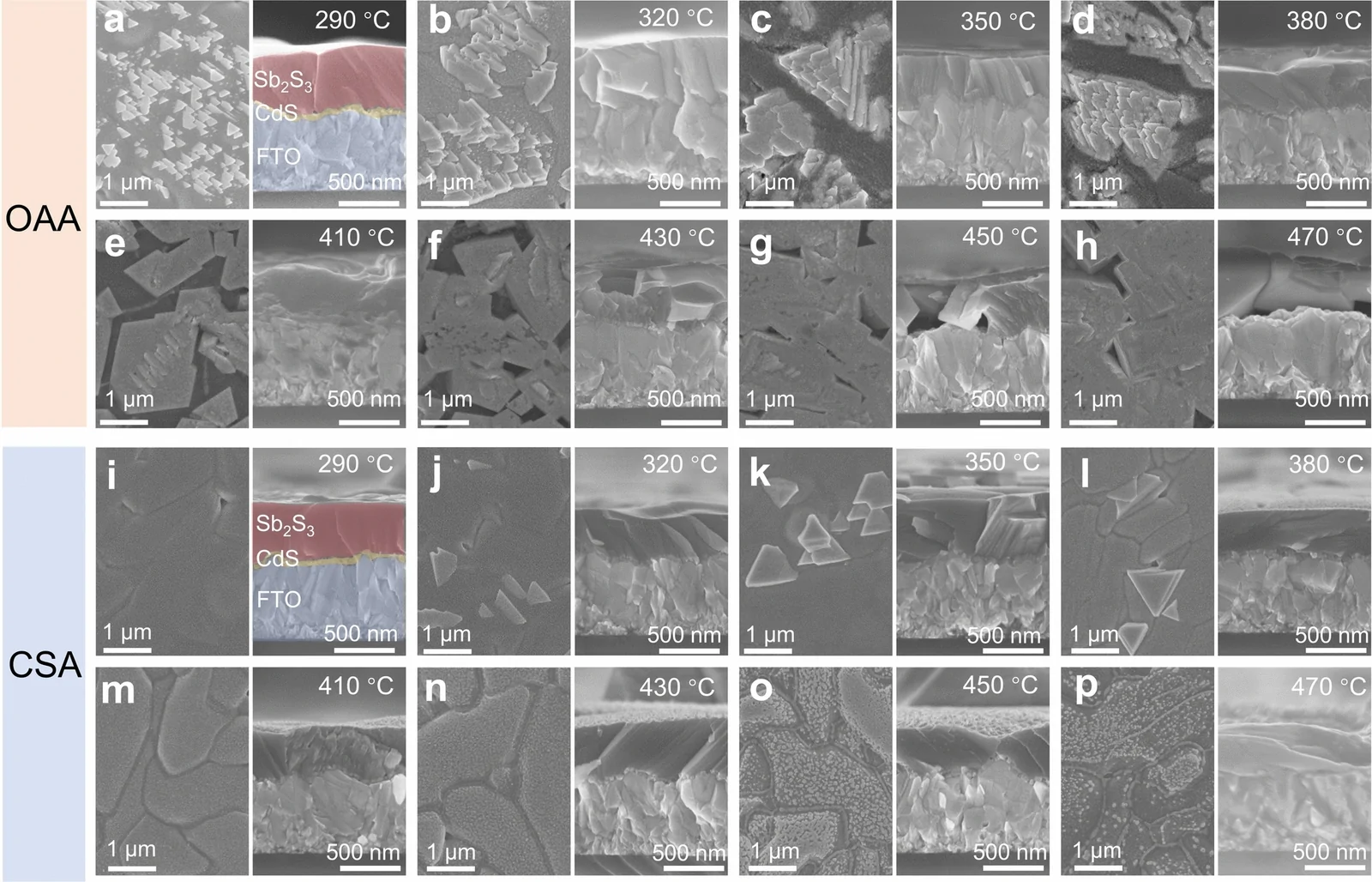
Morphological analysis. The surface and cross-sectional morphology of the Sb_2_S_3_ films annealed with the **a-h** OAA and **i-p** CSA at different temperatures

To elucidate the physical origins of the aforementioned device performance variations, we systematically investigated the effects of different annealing strategies on film morphology, crystallinity, and chemical composition. The superior device performance originates from the effective microstructural control achieved by the CSA strategy. We first examined the film morphology evolution under conventional OAA (samples O290-O470). As shown in the surface SEM images (Fig. 3a-h), uncontrolled oxidation led to the formation of prismatic Sb_2_O_3_ blocks on the film surface even at a low temperature of 290 °C. With increasing temperature from 320 °C, these Sb_2_O_3_ particles coarsened significantly (reaching ~ 1 μm) and covered extensive surface areas. Cross-sectional SEM images (Fig. 3a-h) revealed a more critical issue: Obvious voids and local perforations emerged within the films when the annealing temperature exceeded 350 °C, causing severe film discontinuity. This structural degradation is attributed to the preferential volatilization of Sb_2_S_3_ over Sb_2_O_3_ at high temperatures, as confirmed by vapor pressure calculations based on the Antoine equation (Note S1). In stark contrast, the CSA samples (C290-C470) exhibited markedly different morphological evolution. Surface SEM images (Fig. 3i-p) show that as the annealing temperature increased from 290 to 450 °C, the crystal grain and nano-belts structure at grain boundaries became more developed while surface particle density decreased. Crucially, even after 450 °C annealing, CSA films maintained dense and continuous morphology without the through-holes observed in OAA samples. Although some thinning and nano-belts discontinuity occurred at 470 °C due to volatilization, the film cross section remained intact without perforation defects. To comprehensively evaluate the film uniformity and morphological stability of the CSA strategy under high temperature, we conducted a systematic morphological characterization of the CSA samples that were annealed at 450 °C (C450). As shown in the digital photos in Fig. S12, the surface of the C450 sample with no macroscopic defects. Further large-area optical microscope images (Fig. S13) and low-magnification SEM images (Fig. S14) revealed that the C450 film exhibited a continuous, dense, and uniformly sized surface morphology. In contrast, low-magnification SEM images (Fig. S14a-h) show that the OAA sample is covered with a large area of densely distributed micrometer-sized pores and a continuous layer of large-sized Sb_2_O_3_ blocks, almost completely covering the entire film surface, indicating that oxidation damage has spread from local areas to the entire film surface. Such large-area Sb_2_O_3_ blocks will significantly hinder light absorption and reduce device performance [28]. These findings jointly confirm that the CSA strategy effectively maintained the structural integrity of the film throughout the entire scale from the microscopic to the macroscopic level. The crystallinity of these films was further characterized by X-ray diffraction. As shown in Fig. S15, OAA samples exhibited a gradual improvement in crystallinity with increasing temperature, as evidenced by sharper diffraction peaks. However, for sample O470, the characteristic Sb_2_S_3_ diffraction peaks (PDF#42–1393) nearly disappeared, indicating that severe re-volatilization of Sb_2_S_3_ at high temperatures ultimately undermines the benefits of improved crystallinity. In comparison, CSA samples (Fig. S16) demonstrated significantly enhanced and well-preserved crystallinity across the temperature range. Full-width at half maximum (FWHM) analysis of characteristic peaks (Fig. S17) quantitatively confirmed that CSA samples possess superior crystalline quality compared to the OAA counterparts. To further understand these structural transformations, we employed Raman spectroscopy (Fig. S18). The spectra revealed that increasing annealing temperature enhanced the crystallinity of Sb_2_S_3_ in both OAA and CSA samples. However, in OAA case, we observed a concurrent intensification of Sb_2_O_3_-related peaks, confirming excessive oxidation and accumulation of this inactive phase at high temperatures. This chemical change correlates directly with the morphological degradation as observed in above SEM.

The enhanced microstructural control in CSA films, evidenced by the morphological and crystallographic data discussed above, can be rationalized by the thermodynamic and kinetic interplay illustrated in Figs. S19 and S20. The confined space of the CSA strategy fundamentally alters the energy landscape for recrystallization. As illustrated in Fig. S19, following the Arrhenius equation (Note S2), the atomic diffusion coefficient *D* of Sb_2_S_3_ exhibits a pronounced upward trend with increasing temperature. Crucially, this temperature-dependent rise in *D* directly reflects the boost in atomic kinetic energy at high temperatures, which facilitates atomic migration for grain growth. This creates the optimal recrystallization condition depicted in Fig. S20. A large thermodynamic driving force coincides with high atomic mobility, enabling efficient atomic diffusion to grain nuclei and promoting the growth of large, well-ordered grains, which is consistent with the XRD and SEM observations. Conversely, in OAA, severe material loss at high temperatures disrupts film continuity before this kinetic advantage can be utilized, offsetting any crystallinity gain. Thus, the CSA strategy achieves a critical balance, utilizing high temperature for enhanced crystallization kinetics while the confined geometry preserves morphological integrity, directly leading to the superior carrier transport and collection efficiency observed in the devices.

### Mechanistic Insights into Dynamic In Situ Oxidation

Beyond suppressing volatilization, the confined architecture of the CSA critically limits oxygen supply and gas convection. When the CSA compresses the feature scale to the micrometer level (4.6 ± 0.5 μm), the Grashof number (Note S3) is far below the critical value of natural convection. This suppresses buoyancy-driven macroscopic convection, transforming the mass transport within the gap into a regime dominated by molecular diffusion [41]. The diffusion-limited microenvironment, on the one hand, causes volatile Sb_2_S_3_ to accumulate on the film surface, forming a local high-pressure zone approaching the equilibrium vapor pressure, thereby inhibiting further volatilization. On the other hand, it significantly reduces the transport flux of atmospheric oxygen to the film surface, preventing uncontrollable bulk-phase oxidation. Consequently, almost no detectable Sb_2_O_3_ forms during CSA annealing under confinement (natural cooling sample), as displayed in Fig. S21. However, the high-annealing temperature inevitably promotes Sb-S bond breaking, generating abundant V_S_. DFT calculations show that V_S_ exhibits the lowest formation energy among intrinsic defects in Sb_2_S_3_ (Fig. S22), far lower than that in traditional semiconductors such as silicon (> 3 eV) [42–44]. These vacancies act as deep donors and create strong non-radiative recombination, severely limiting carrier lifetime [45].

**Fig. 4:**
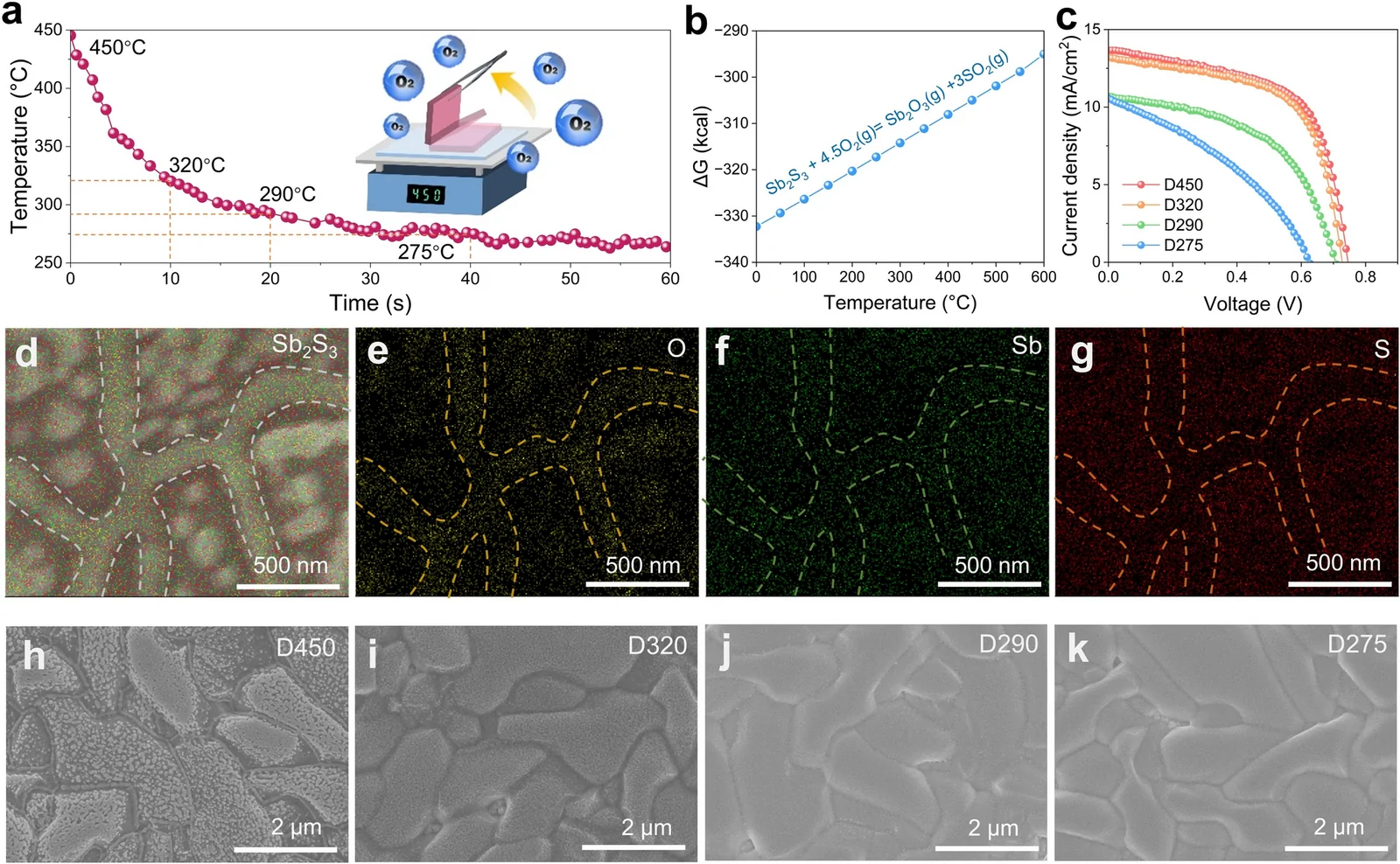
Dynamic in situ oxidation mechanism and performance correlation analysis. **a** Cooling time plots. **b** Gibbs free energy. **c** *J*-*V* curves of the devices based on DISO at different temperatures. The EDS mapping images of C450 sample: **d** Sb_2_S_3_, **e** O, **f** Sb, and **g** S. **h–k** SEM images corresponding to different lifting temperature of the samples

To mitigate this, post-CSA cover removal at high temperature enables controlled in situ oxidation and effective V_S_ passivation, which is displayed in Fig. 4a. The cooling profile after recrystallization creates a dynamic temperature window (450–275 °C within 40 s) for what we term dynamic in situ oxidation (DISO), in which the lip was removed at those target temperatures. Thermodynamically, Sb_2_S_3_ oxidation is spontaneous (ΔG < 0, as shown in Fig. 4b) in the above temperature range, indicating the feasible oxygen passivation during the DISO. From a kinetic perspective, as the temperature decreases, the reaction rate will decrease [46]. As a result, these delayed-exposure experiments reveal that immediate cover removal at 450 °C (D450) yields an optimal device PCE, with champion *V*_OC_ and *J*_SC_. The PCE declines systematically as the exposure temperature is lowered (D320, D290, D275), as shown in the *J-V* curve of Fig. 4c. When the delay temperature reached 275 °C, the benefits of defect passivation were completely lost, ultimately resulting in significantly inferior device performance compared to the samples with immediate lid removal, confirming that 450 °C immediate exposure provides the optimal balance between sufficient oxidation kinetics and avoidance of over-oxidation.

Material characterization directly links this trend to surface composition and morphology. As shown in Fig. 4d-g, the SEM and EDS spectra reveal the distribution characteristics of the components on the surface of D450. The oxygen element signal is mainly concentrated in the grain boundary regions, which highly coincides with the positions of the Sb_2_O_3_ nano-belts. Meanwhile, granular Sb_2_O_3_ is sporadically distributed on the grain surface. This confirms that high-temperature-induced DISO is the core feature of CSA. The SEM images of samples with different lid-opening temperature (Fig. 4h-k) visually present the characteristics of Sb_2_O_3_ particles and Sb_2_S_3_ grains. As the exposure temperature decreases, the population and size of these Sb_2_O_3_ particles diminish sharply. At 275 °C (sample D275), the surface is nearly devoid of Sb_2_O_3_ and appears smooth due to insufficient reaction kinetics temperature.

We therefore propose a two-step mechanism of the DISO. Upon atmospheric exposure, oxygen first rapidly fills V_S_ sites, passivating deep-level defects. Sustained high temperature then drives selective oxidation at defect-rich regions, resulting in epitaxial Sb_2_O_3_ nano-belts at grain boundaries and a surface mixed phase. This process simultaneously passivates defects and enhances light scattering, boosting *V*_OC_ and *J*_SC_. However, lower temperatures slow oxidation kinetics, reducing Sb_2_O_3_ formation and also diminishing passivation. This DISO strategy thus achieves precise modification, avoiding the detrimental over-oxidation of conventional OAA.

### Correlative Physical Analysis Linking Oxidation to Device Performance

**Fig. 5:**
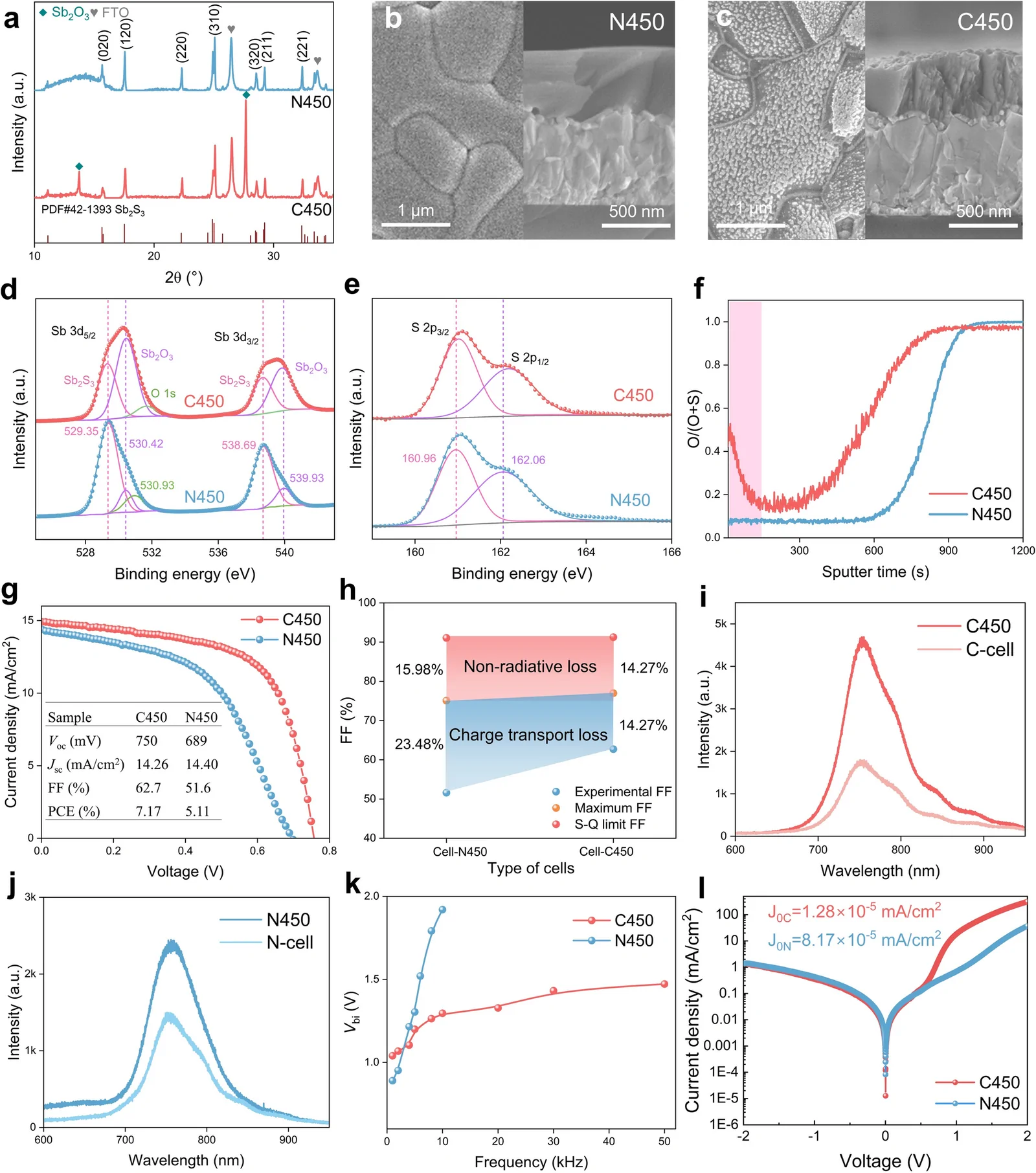
Structure and performance correlation analysis. **a** XRD patterns. **b, c** Surface and cross-sectional SEM images. High-resolution XPS spectra of **d** Sb 3*d* and **e** S 2*p.* **f** O/(O + S) counts ratio. **g** *J*-*V* curves. **h** FF loss analysis. **i, j** Photoluminescence spectra, **k** *V*_bi_ with frequency ranging from 1 to 50 kHz and **l** Dark *J*-*V* curves of N450 and C450 device

To definitively establish the role of ambient oxygen in the CSA process, a critical control experiment was conducted. Sb_2_S_3_ precursor films were annealed at 450 °C under CSA geometry in two distinct atmospheres: a strictly oxygen-free environment (N_2_ glove box, O_2_ < 10 ppm) and ambient air, yielding the N450 and C450 samples, respectively. XRD analysis confirmed that annealing in N_2_ enhanced crystallinity without inducing oxidation, whereas annealing in air led to detectable oxide formation (Fig. 5a). This compositional difference manifested directly in film morphology, cross-sectional SEM revealed that N450 formed a smooth, dense film (~ 500 nm), while C450 exhibited a distinct surface layer rich in Sb_2_O_3_ (Fig. 5b, c).

XPS and SIMS characterizations were employed to clarify the chemical states and distribution of incorporated oxygen. Full XPS spectra (Fig. S23) confirmed that both films contained only Sb, O, and S (no impurities). In the high-resolution XPS spectra of Sb 3*d* (Fig. 5d), the weak peaks at ~ 530.42 and ~ 539.93 eV correspond to Sb^3+^ in Sb_2_O_3_. The intensities of the Sb 3*d*_5/2_ and Sb 3*d*_3/2_ peaks associated with Sb_2_O_3_ are significantly enhanced compared to N450. Meanwhile, the peak at ~ 530.93 eV is assigned to the O 1* s* signal originating from lattice oxygen in the surface oxide [25, 47]. Moreover, the S 2*p* double peaks of C450 (Fig. 5e) slightly shift toward higher binding energy [14], indicating a decrease in the electron density around the S atoms, which is attributed to the substitution of some S vacancies in Sb_2_S_3_ by O atoms to form O_S_. SIMS depth profiling quantitatively mapped a gradient oxygen distribution in C450, featuring a surface Sb_2_O_3_-rich region (O/(O + S) > 0.5) overlying a bulk zone with moderate O_S_ doping (0.1 < O/(O + S) < 0.3) (Figs. S24, S25, and 5f). This tailored oxygen profile, absent in the uniformly low-oxygen N450 film, enables simultaneous passivation of bulk and surface defects.

The optoelectronic impact of this oxygen incorporation is profound, as reflected in the *J-V* curve of Fig. 5g. The champion C450 device achieved a PCE of 7.17%, a ~ 30% enhancement over the N450 baseline (5.11%). The marked improvements in *V*_OC_ (750 vs. 689 mV) and FF (62.7% vs. 51.6%) for C450 signal substantially suppressed carrier recombination by DISO, which is confirmed by the statistical results of the cells in Fig. S26. This is the highest reported PCE for all non-vacuum-processed, carbon-based Sb_2_S_3_ devices completely fabricated in an ambient atmosphere (Note S4). In addition, the tiny improvement in *J*_SC_ is validated via integrated currents from EQE response (Fig. S27), while lower Urbach energy for C450 (Fig. S28) confirms oxygen passivation reduced defect density. The transmittance of the films was tested by a UV–Vis–near-infrared spectrophotometer (Fig. S29). The optical band gap was calculated using the Tauc formula (Fig. S30), and the E_g_ of C450 was found to be 1.77 eV, while that of N450 was 1.75 eV. The slight difference in band gap indicates that the primary role of oxygen incorporation is defect passivation rather than bandgap engineering. Detailed loss analysis attributed this to reductions in both non-radiative recombination loss (from 15.98% to 14.27%) and transport loss (from 23.48% to 14.27%) (Fig. 5h, Note S5, and Table S3) [48]. Carrier dynamics and defect state modulation were further investigated. Photoluminescence (PL) spectroscopy confirmed that C450 films had stronger luminescence, indicating fewer defects (Fig. 5i, j). When integrated into full devices (FTO/CdS/Sb_2_S_3_/PbS/Carbon), C450 retained only 38% of its film PL intensity versus 60% for N450, demonstrating superior carrier extraction. Light intensity-dependent measurements revealed C450 had higher carrier collection efficiency (0.98 vs. 0.95) and a lower ideality factor (n = 1.88 vs. 1.94), indicating suppressed Shockley–Read–Hall recombination (Figs. S31 and S32; Notes S6 and S7) [49, 50]. This is supported by transient photocurrent (TPC) and transient photovoltage (TPV) results (Figs. S33 and S34). C450 demonstrates a faster carrier extraction rate (τ_CC_ = 1.33 μs, compared to 1.51 μs for N450). Meanwhile, the short decay constants (τ_VC1_ = 26.5 μs for C450, τ_VN1_ = 78.2 μs for N450) relate to interface recombination, with C450’s smaller value stemming from shallow defects formed by DISO-induced non-radiative relaxation [51]. Its long decay constants (τ_VC2_ = 1.02 ms, compared to 0.85 ms for N450) indicate that the overall recombination process is significantly inhibited, ultimately enhancing device PCE [51].

Electrical characterizations of the Sb_2_S_3_ films and devices provide consistent evidence. Conductive atomic force microscopy (c-AFM) shows that the current flow within the C450 grains is relatively higher (Figs. S35–S38). This is attributed to the Sb_2_O_3_ nano-belts at the grain boundaries, which act as insulating barriers, blocking the leakage current path. At the back interface, they prevent electrons and tunneling holes from passing through, effectively suppressing carrier recombination and improving the hole extraction efficiency. In combination with the defect passivation effect of the bulk O_S_ doping, the *V*_OC_ and FF of the device have been effectively enhanced [20, 25, 52]. Dark capacitance–voltage (*C*-*V*) measurements (Fig. S39) and derivatives of 1/*C*^2^-*V* curves (Fig. S40, Note S8) confirmed C450 possessed a higher built-in potential (*V*_bi_ = 1.04 vs. 0.89 eV) and acceptor density (*N*_A_ = 1.83 × 10^17^ vs. 9.11 × 10^16^ cm^−3^), confirming defect passivation and the creation of beneficial shallow acceptors [53–55]. Variable-frequency *C*-*V* tests revealed a more stable *V*_bi_ for C450 at high frequencies, indicating fewer deep traps, compared with abundant deep traps that induced a strong dependent relation in N450 (Figs. S41, S42, and 5k). Finally, device-level dark *J-V* analysis (Fig. 5l) and electrochemical impedance spectroscopy (EIS, Fig. S43, Table S4) also confirmed C450 exhibited a lower reverse saturation current (1.28 × 10^–5^ vs. 8.17 × 10^–5^ mA cm^−2^), reduced charge transfer resistance, and lower recombination loss. These indicate that the carrier recombination in the C450 cell has been significantly alleviated [48]. Based on the relationship between the *J*_0_ and *V*_OC_ (Note S9), the lower *J*_0_ of the C450 cell also leads to a higher *V*_OC_. Collectively, oxygen passivation alleviates deep defects, enhances conductivity, suppresses carrier recombination, and thus improves the PCE. The long-term operational stability (maximum power point tracking) of CSA devices was evaluated under continuous AM 1.5G illumination (100 mW cm^−2^) at room temperature for 7 days. As shown in Fig. S44, unencapsulated C450 devices retained 94.4% of their initial PCE after 170 h of continuous light soaking, significantly outperforming the N450 control devices, which degraded to 91.3% under identical conditions. This contrast highlights the superior stability imparted by the CSA strategy. To correlate this performance retention with microstructural evolution, we examined the surface morphology of the aged films by SEM. Figure S45 shows the C450 film surface after 0, 1, and 7 days of continuous illumination. Remarkably, the Sb_2_O_3_ nano-belts at grain boundaries remain intact and clearly visible throughout the test period, with no signs of coarsening, detachment, or new defect formation. This morphological stability confirms that the nano-belts are chemically and structurally robust under continuous light exposure.

**Fig. 6:**
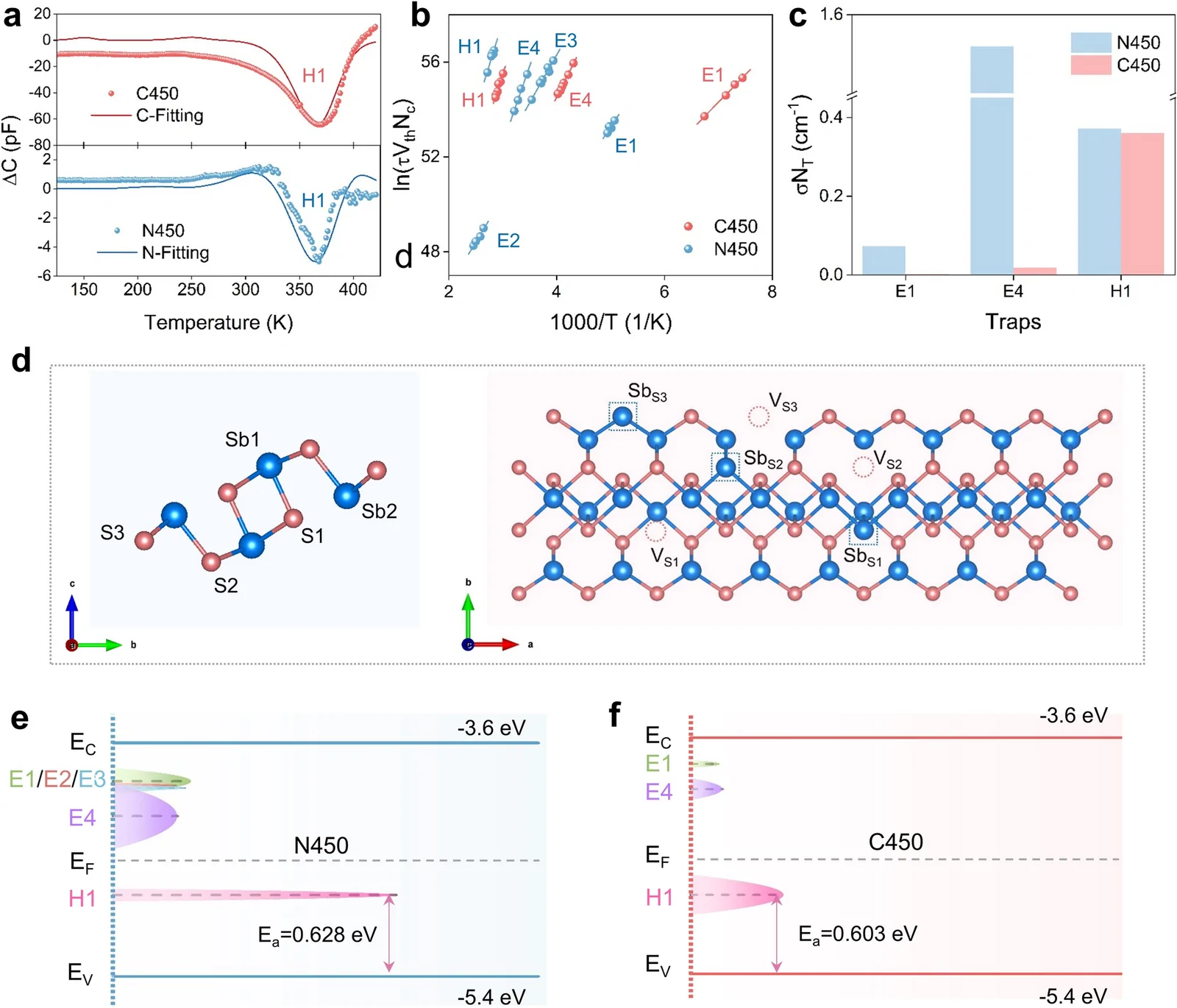
Characterizations and analysis of deep-level defects. **a** O-DLTS signals and high-resolution evaluation simulations based on N450 and C450. **b** The corresponding Arrhenius plots obtained from O-DLTS signals. **c** Histogram of the calculated σ × N_T_ values of different hole traps based on N450 and C450. **d** Schematic diagram of Sb_2_S_3_ with five non-equivalent atomic sites and defects in Sb_2_S_3_ lattice. **e, f** Energy states and defect levels of the devices based on N450 and C450

Building upon the established performance enhancement via oxygen passivation, a detailed analysis of defect energy levels provides fundamental insight into the improved carrier dynamics [54, 55]. We conducted defect analysis on the C450 and N450 devices using optical deep-level transient spectroscopy (O-DLTS) (the test conditions and information as shown in Note S10). Figure 6a shows the O-DLTS spectra of C450 and N450 samples at different pulse voltages. The negative peaks in the DLTS spectra correspond to hole traps [56]. According to the linear fittings of the Arrhenius plots (Fig. 6b), the N450 film exhibits a complex defect landscape, including four electron traps (E1-E4) and one hole trap (H1). In contrast, the C450 film only exhibits two electron traps (E1 and E4) and one hole trap (H1). The defect parameters obtained from O-DLTS and Arrhenius analysis are summarized in Table S5. Here, E_T_, σ, and N_T_ represent the trap level, capture cross section, and trap density, respectively. Further analysis of the defect density and carrier recombination characteristics reveals that the trap density of H1 in N450 is as high as 1.33 × 10^15^ cm^−3^, while it drops to 3.44 × 10^14^ cm^−3^ in C450. The trap densities of E1 and E4 also significantly decrease. Additionally, from σN_T_ (Fig. 6c), it can be seen that σN_T_ of E1 in C450 is almost zero, while that of E4 significantly decreases. The carrier lifetime related to trap-assisted Shockley–Read–Hall (SRH) recombination (τ_trap_) is inversely proportional to the product of σN_T_ [57]. Therefore, the σN_T_ of E1, E4, and H1 in C450 is reduced by an order of magnitude compared to N450, thereby significantly extending the carrier lifetime. Specifically, the hole energies of E1, E4, and H1 are higher than the valence band, and thus, they usually act as recombination centers due to their high ionization energy [7].

As shown in Fig. 6d, each Sb_2_S_3_ atom consists of five inequivalent atomic sites, namely Sb1, Sb2, S1, S2, and S3. The E1, E4, and H1 traps in this study may originate from the interstitial defect (Sb_i_), V_S_, and the substitutional defect (Sb_S_), respectively. We provide the experimentally determined level alignment results of these devices (Fig. 6e, f). In N450, traps E1/E2/E3 are attributed to Sb_i_, located 0.295, 0.326, and 0.347 eV below the conduction band minimum (CBM), respectively. E4 corresponds to a V_S_ at 0.564 eV below the CBM, while H1 is a Sb_S_ at 0.628 eV above the valence band maximum (VBM) [58]. In sharp contrast, the defect profile of the C450 sample is significantly simplified and shallower. The deep electron traps E2 and E3 are completely absent, leaving only E1, E4, and H1 coexisting. Additionally, the remaining defect levels shift toward the band edges. H1 is 0.603 eV above the VBM, and the energies of E1 and E4 have become shallower. This reconstructed defect configuration indicates effective passivation and modification of deep-level states in C450. From a physical perspective, in Sb-rich Sb_2_S_3_, the excess antimony atoms preferentially fill the V_S_ to form Sb_S_ rather than form interstitial Sb_i_, because the formation energy of Sb_S_ is lower than that of Sb_i_, making V_S_ and Sb_S_ the main defects [7]. Both are deep-level defects with large capture cross sections and high trap densities, acting as SRH recombination centers, seriously hindering carrier transport and shortening the lifetime [7, 33, 59]. The C450 sample, through targeted oxygen incorporation, achieves a dual benefit: It passivates V_S_ defects and concurrently restructures the defect ensemble, reducing both the density and carrier-capture activity of these dominant recombination centers. This fundamental improvement in electronic quality provides the definitive microscopic explanation for the enhanced carrier transport and superior device performance documented throughout this study.

## Conclusion

This work presents a CSA strategy that establishes a well-controlled microenvironment through simple physical constraints, enabling simultaneous enhancement of crystallization and defect passivation of Sb_2_S_3_ thin films in ambient air. The strategy operates via a dual-action mechanism: It suppresses high-temperature volatilization of Sb_2_S_3_, reducing sulfur vacancy formation, while simultaneously guiding oxygen atoms to preferentially fill these vacancies and inhibit the generation of interstitial antimony defects, thereby minimizing carrier capture by deep-level recombination centers. The CSA approach enables concurrent recrystallization and controlled oxidation. This process leads to the formation of dense Sb_2_O_3_ nano-belts at grain boundaries, which block leakage current paths and suppress interface recombination. Consequently, carbon-based Sb_2_S_3_ solar cells fabricated using this CSA approach achieve a PCE of 7.17% (*V*_OC_ = 750 mV, *J*_SC_ = 14.26 mA cm^−2^, FF = 62.7%), representing a 40.3% improvement over devices annealed in N_2_. This work elucidates the role of oxygen passivation in antimony chalcogenide solar cells and demonstrates that precisely controlled in situ oxidation is an effective approach for enhancing device performance, offering a scalable pathway for atmosphere-processable solar cells.

## Supplementary Information

Below is the link to the electronic supplementary material.

Supplementary file1 (DOCX 25313 KB)
