# Supplementary material for: Ambient Confined-Space Annealing for Crystallization Enhancement and Defect Passivation in Sb2S3 Thin-Film Solar Cells
Source: Nanomicro Lett. 2026 May 8;18:359. doi: 10.1007/s40820-026-02193-w (PMC13156396; doi:10.1007/s40820-026-02193-w)
Supplement: Supplementary file 1 — Supplementary file1 (DOCX 25313 KB) [file 40820_2026_2193_MOESM1_ESM.docx]

Supporting Information for

**Ambient Confined-Space Annealing for Crystallization Enhancement and Defect Passivation in Sb_2_S_3_ Thin-Film Solar Cells**

Li-Mei Lin^1#^, Jie Huang^1#^, Hu Li^1^, Jin-Rui Cai^2^, Shui-Yuan Chen^2^, Jian-Min Li^3, *^, Xiao-Min Wang^4, *^, Gui-Lin Chen^1, 2,^ ^*^

^1^ College of Physics and Energy, Fujian Normal University, Fuzhou 350117, P. R. China

^2^ Fujian Provincial Engineering Technology Research Center of Solar Energy Conversion and Energy Storage, Fujian Normal University, Fuzhou 350117, P. R. China

^3^ Key Laboratory of Artificial Micro and Nano-structures of Ministry of Education, School of Physics and Technology, Wuhan University, Wuhan 430072, P. R. China

^4^ Hubei Key Laboratory of Plasma Chemistry and Advanced Materials, School of Materials Science and Engineering, Wuhan Institute of Technology, Wuhan 430205, P. R. China

^#^Li-Mei Lin and Jie Huang contributed equally to this work.

^*^Corresponding authors. E-mail: [ljmphy@whu.edu.cn](mailto:ljmphy@whu.edu.cn) (Jian-Min Li); [wxm@wit.edu.cn](mailto:wxm@wit.edu.cn) (Xiao-Min Wang); [glchen@fjnu.edu.cn](mailto:glchen@fjnu.edu.cn) (Gui-Lin Chen)

**Note S1**

Calculation of the saturated vapor pressure.

The saturated vapor pressures of Sb_2_S_3_ and Sb_2_O_3_ can be calculated using the Antony equation [S1]. **Formula S1** is as follows:

$\log\frac{P}{6895}=A-\frac{B}{T}$**(S1)**

Among them, the saturated vapor pressure P is in Pa, parameter 6895 is the conversion factor between the pressure units psi and Pa, A and B are the parameters of the curve to be determined, and T is the Kelvin thermodynamic temperature.

The parameters A and B of Sb_2_S_3_ are 13.93 and 10490, respectively, and those of Sb_2_O_3_ are 3.885 and 4417, respectively.

**Note S2** Calculation of the relationship between the diffusion coefficient and temperature.

The variation law of the diffusion coefficient with temperature can be described by the Arrhenius equation [S2]. **Formula S2** is as follows:

$D=D_{0}\exp\left( -\frac{Q}{RT} \right)$**(S2)**

Here, *D*_0_ refers to the pre-factor, *Q* is the activation energy, and *R* is the general gas constant (where the value of *R* is the product of Avogadro's constant and Boltzmann's constant), with the unit being J/mol. *D*_0_ and *Q* are temperature-independent constants.

**Note S3** Calculation of air kinematic viscosity.

1. Schematic diagram of annealing strategy for thin atmospheric interlayers.


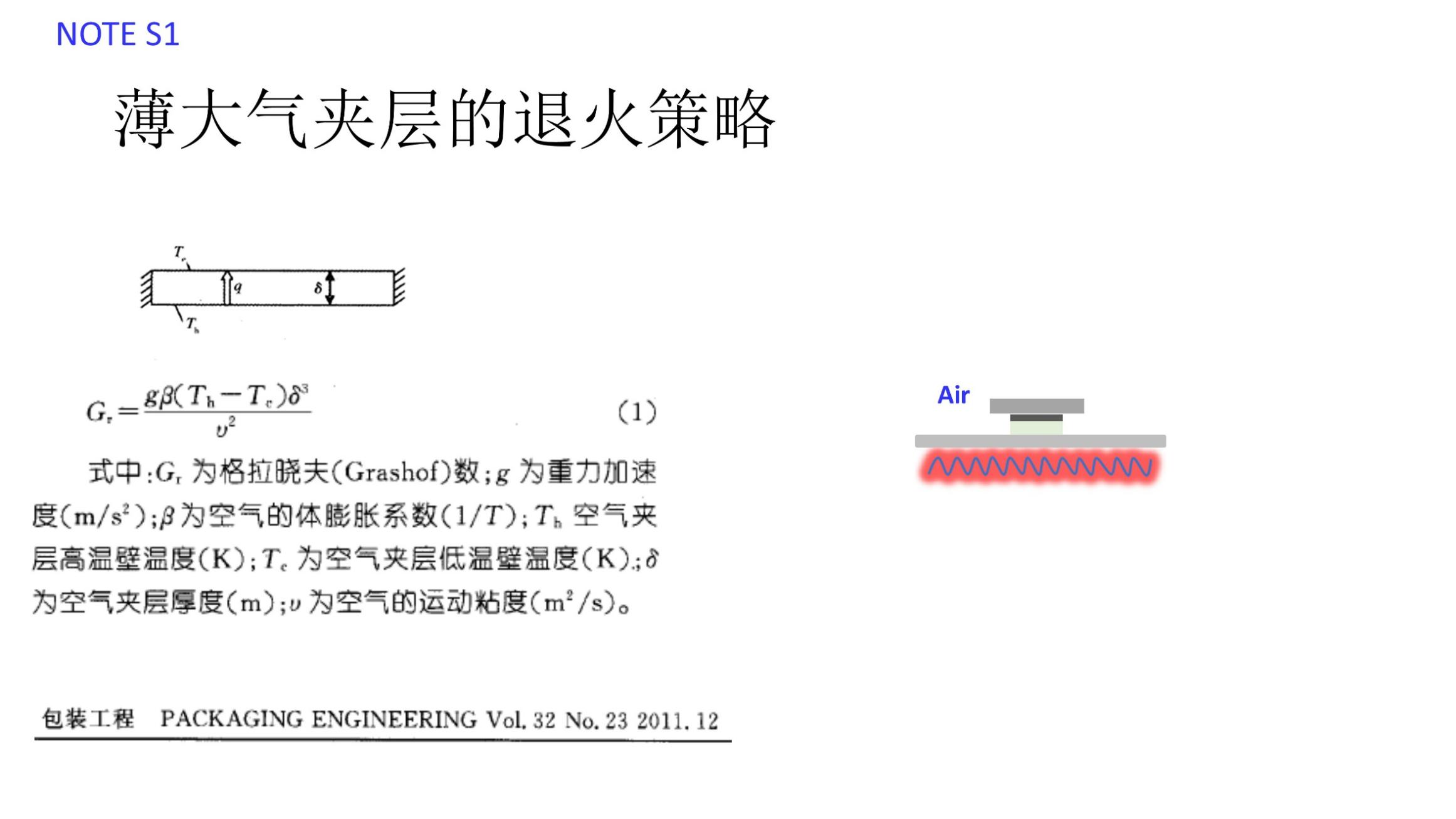


1. Schematic diagram of horizontal mezzanine.


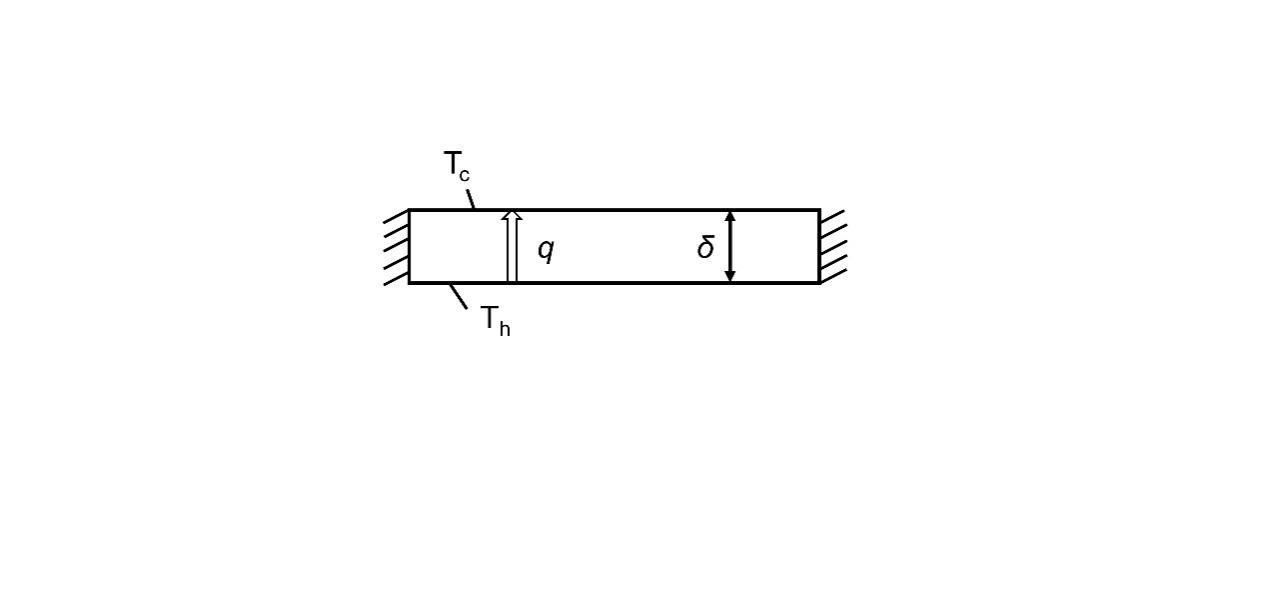


1. Grashof formula [S3]. **Formula S3** is as follows:

$G_{r}=\frac{g\beta(T_{h}-T_{c})\delta^{3}}{v^{2}}$**(S3)**

*G*_r_ is the Grashof number, *g* is the air acceleration, *β* is the volume expansion coefficient of air, *T*_h_ is the high-temperature wall temperature of the air interlayer, *T*_c_ is the low-temperature wall temperature of the air interlayer, *δ* is the thickness of the air interlayer, and *v* is the kinematic viscosity of the air.

In conventional OAA, due to the absence of physical confinement, the characteristic length *δ* is at the macroscopic scale (centimeter level), resulting in *G*_r_ being much higher than the critical value for the onset of natural convection, thereby triggering strong buoyancy-driven convection. However, in the CSA configuration, the confined gap is compressed to 4.6±0.5 μm, reducing the characteristic length by approximately four orders of magnitude. This leads to a sharp decrease in *G*_r_ to a level far below the critical threshold for natural convection, effectively suppressing macroscopic fluid motion. At this point, the mass transport mode within the gap shifts from convection-dominated to molecular diffusion-dominated.

**Note S4** Summary of the research work of Sb_2_S_3_ solar cells using carbon or Au as a back electrode in N_2_, Ar or Air.


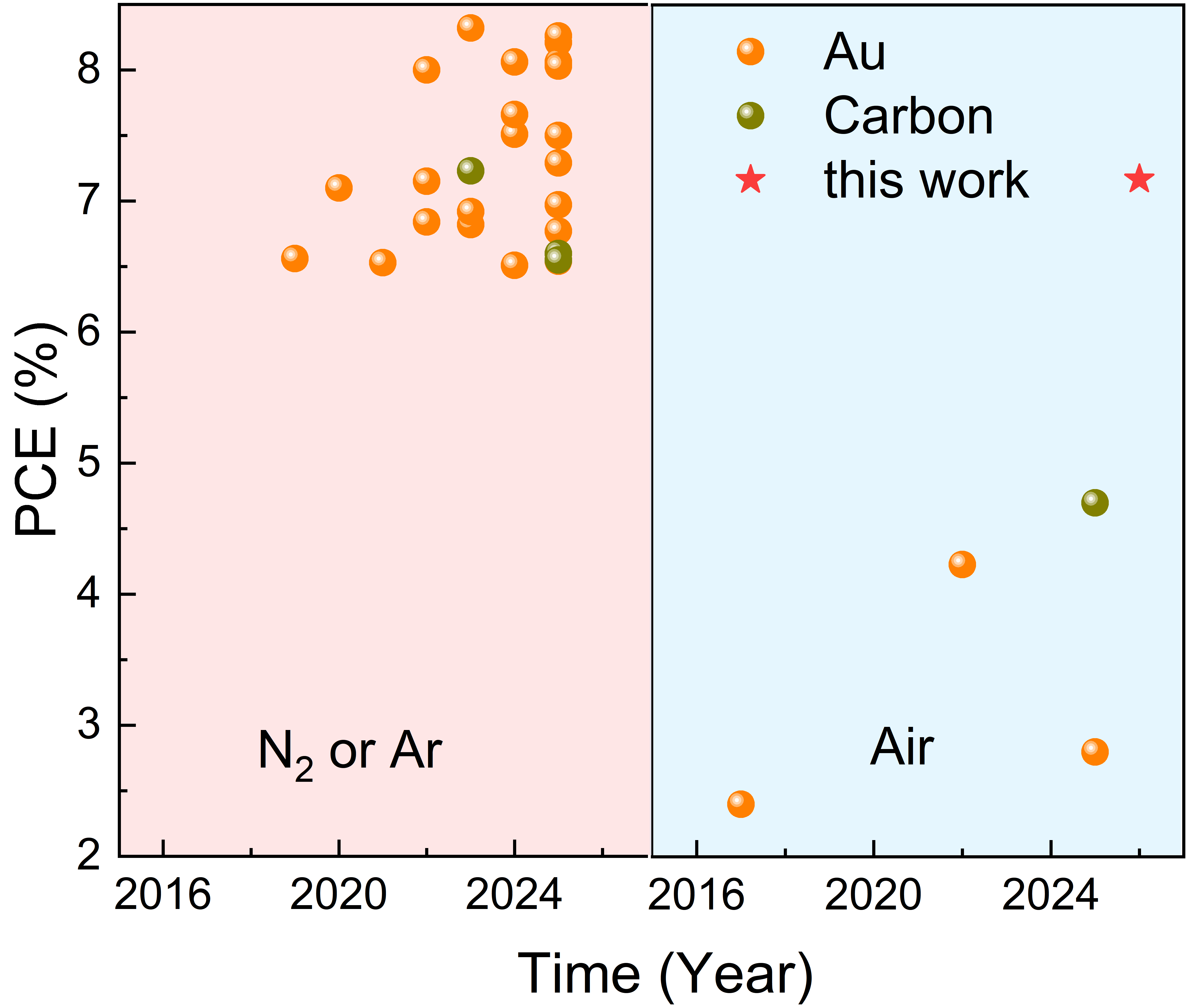


This work successfully fabricated a carbon-based Sb_2_S_3_ solar cell using the CSA strategy, achieving a certified efficiency of 7.17%. Through literature review, this efficiency represents the highest record among all carbon-based Sb_2_S_3_ devices that have been fabricated under air atmosphere conditions (without any vacuum steps).

| Device Structure | Year | PCE [%] | Atmosphere | REF No. |
| --- | --- | --- | --- | --- |
| FTO/TiO_2_/Sb_2_S_3_/P3HT/Au | 2017 | 2.40 | Air | [S8] |
| FTO/TiO_2_/CdS/Sb_2_S_3_/Au | 2022 | 4.23 | Air | [S9] |
| FTO/TiO_2_/Sb_2_S_3_/P3HT/Au | 2025 | 2.8 | Air | [S10] |
| FTO/CdS/Sb_2_S_3_/MoO_x_/Carbon | 2025 | 4.7 | Air | [S11] |
| FTO/CdS/Sb_2_S_3_/PbS/Carbon | 2026 | 7.17 | Air | This work] |
| FTO/TiO_2_/Sb_2_S_3_/Spiro-OMeTAD/Au | 2019 | 6.56 | Ar | [S12] |
| FTO/CdS/Sb_2_S_3_/Spiro-OMeTAD/Au | 2025 | 7.29 | Ar | [S13] |
| FTO/TiO_2_/CdS/Sb_2_S_3_/Spiro-OMeTAD/Au | 2021 | 6.53 | Ar+H_2_ | [S14] |
| FTO/TiO_2_/Sb_2_S_3_/Spiro-OMeTAD/Au | 2020 | 7.10 | N_2_ | [S15] |
| ITO/CdS:In/Sb_2_S_3_/Spiro-OMeTAD/Au | 2022 | 7.15 | N_2_ | [S16] |
| FTO/SnO_2_/CdS/Sb_2_S_3_/Spiro-OMeTAD/Au | 2022 | 6.84 | N_2_ | [S17] |
| FTO/CdS/Sb_2_S_3_/Spiro-OMeTAD/Au | 2022 | 8.00 | N_2_ | [S18] |
| FTO/TiO_2_/Sb_2_S_3_+MAPbI_3_/Spiro-OMeTAD/Au | 2023 | 8.32 | N_2_ | [S19] |
| FTO/CdS/Sb_2_S_3_/Spiro-OMeTAD/Au | 2023 | 6.82 | N_2_ | [S20] |
| FTO/CdS/Sb_2_S_3_/Spiro-OMeTAD/Au | 2023 | 6.92 | N_2_ | [S21] |
| FTO/SnO_2_/CdS/Sb_2_S_3_/BTR-TPA/Spiro-OMeTAD/Au | 2024 | 7.51 | N_2_ | [S22] |
| FTO/SnO_2_/CdS/Sb_2_S_3_/Spiro-OMeTAD/Au | 2024 | 7.66 | N_2_ | [S23] |
| FTO/CdS/Sb_2_S_3_/Spiro-OMeTAD/Au | 2024 | 6.51 | N_2_ | [S24] |
| FTO/CdS/Sb_2_S_3_/SAM/Spiro-OMeTAD/Au) | 2024 | 8.06 | N_2_ | [S25] |
| FTO/TiO_2_/ZnO/Sb_2_S_3_/P3HT/Au | 2025 | 7.5 | N_2_ | [S26] |
| FTO/CdS/Sb_2_S_3_ (PEAI)/Spiro-OMeTAD/Au | 2025 | 8.21 | N_2_ | [S27] |
| FTO/c-TiO_2_/TiO_2_-CdS-NA/Sb_2_S_3_/Spiro-OMeTAD/Au | 2025 | 8.06 | N_2_ | [S28] |
| FTO/SnO_2_/CdS/Sb_2_S_3_/PbS-EDT/Au | 2025 | 8.26 | N_2_ | [S29] |
| FTO/TiO_2_/Sb_2_S_3_/Spiro-OMeTAD:TMT-TTF/Au | 2025 | 6.97 | N_2_ | [S30] |
| FTO/SnO_2_/CdS/Sb_2_S_3_/Spiro-OMeTAD/Au | 2025 | 8.03 | N_2_ | [S31] |
| FTO/CdS/Sb_2_S_3_/Spiro-OMeTAD/Au | 2025 | 6.54 | N_2_ | [S32] |
| FTO/SnO_2_/CdS/Sb_2_S_3_ (SDBS)/Spiro-OMeTAD/Au | 2025 | 6.77 | N_2_ | [S33] |
| ITO/TiO_2_/CdS/Sb_2_S_3_/Carbon/Ag | 2023 | 7.23 | N_2_ | [S34] |
| FTO/CdS/Sb_2_S_3_/MnS−PbS/Carnon | 2025 | 6.60 | N_2_ | [S35] |
| FTO/CdS/Sb_2_S_3_/MnS/Carbon | 2025 | 6.55 | N_2_ | [S36] |

**Note S5** Charge-transport losses analysis.

1. Shockley-Queisser limit FF

When both transport losses and non-radiative losses are absent, the FF for solar cells operating at the radiative limit (i.e., the Shockley-Queisser limit) can be computed via a publicly accessible Python script (<https://github.com/marcus-cmc/Shockley-Queisser-limit>) [4]. The FF at SQ limits corresponding to samples C450 (1.77eV) and N450 (1.75eV) in this study were 91.24% and 91.06%, respectively.

1. Theoretical FF

The theoretical FF without charge-transport losses was computed using the following **Formulas S4-S5**:

${FF}_{max}=\frac{v_{OC}-\ln(v_{OC}+0.72)}{v_{OC}+1}$**(S4)**

$v_{OC}=\frac{V_{OC}}{nk_{B}T/q}$**(S5)**

where *n* is ideality factor, *V*_OC_ is open-circuit voltage, *k_B_* is the Boltzmann constant, T is the temperature, *q* is the elementary charge. In this work, the *V*_OC_ values of the C450 and N450 samples were 750 mV and 689 mV, with *n* values of 1.88 and 1.94 respectively. The theoretical FF_max_ calculated were 76.97% and 75.08%.

3. Experimental FF

The FF of C450 is 62.7%, and that of N450 is 51.6%.

**Note S6** The relationship between *J*_SC_ of the cell and the irradiation light intensity.

The relationship between *J*_SC_ and *V*_OC_ of the cell and the irradiation light intensity can reveal the characteristics of carrier transport and extraction efficiency [S5]. The function relationship between *J*_SC_ of the solar cell and the light intensity is as follows (**Formula S6**):

$J_{\mathrm{sc}}\propto I^{\alpha}$**(S6)**

where *I* represent the percentage of irradiation light intensity, and α is the power index. The α values of C450 and N450 devices can be obtained by fitting a formula, which are 0.98 and 0.95, respectively.

**Note S7** The relationship between *V*_OC_ of the cell and the irradiation light intensity.

As shown in **Formula S7**, the function relationship between *V*_OC_ of the solar cell and the light intensity is [S6]:

$V_{OC}=\frac{nk_{B}T}{q}lnI+C$**(S7)**

where T, k_B_, and q are the Kelvin temperature, Boltzmann constant, and elementary charge, respectively, and n is the ideality factor related to recombination. The ideality factor n of C450 and N450 devices can be obtained by fitting, which are 1.88 and 1.94, respectively.

**Note S8** The relationship between the 1/*C*^2^-*V* curve and *V*_bi_.

The 1/*C*^2^-*V* curves were plotted for the test data, and the linear segments of the curves were fitted and extrapolated based on **Formula S8** [S7]:

$\frac{1}{C^{2}}=\frac{2(V_{bi}-V)}{qS^{2}\varepsilon_{0}\varepsilon_{p}N_{A}}$**(S8)**

where *C* is the capacitance, *V* is the applied DC bias voltage, S (0.09 cm²) is the cell area, ε₀ (8.854 × 10^-14^ F/cm) is the vacuum permittivity, and εₚ (6.67) is the relative permittivity of Sb_2_S_3_.

**Note S9** The relationship between *J*_0_ and *V*_OC_.

The reverse saturation current of the cell also reflects the *V*_OC_ closely related to carrier recombination [S5], the **Formula S9** as follows:

$V_{OC}=\frac{Ak_{B}T}{q}\ln\left( \frac{J_{SC}}{J_{0}}+1 \right)$**(S9)**

where A is the diode ideality factor, and *J*_0_ is the reverse saturation current. The lower reverse saturation current of the C450 cell will result in a higher *V*_OC_, which is consistent with the photovoltaic test results of the device.

**Note S10** DLTS measurement configuration and trap type determination.

The deep-level transient spectroscopy (DLTS) measurement was carried out using the Phystech FT-1230 HERA DLTS system. The temperature scanning range was from 120 K to 425 K, with a step size of 2 K. The optical excitation mode (O-DLTS) was employed, using a 650 nm laser diode as the excitation source to preferentially fill the minority carrier traps.

In the DLTS spectrum, the sign of the signal is determined by the direction of the capacitance transient when the traps emit carriers. For p-type Sb_2_S_3_, the majority carriers are holes. Under optical excitation, the photogenerated minority carriers are captured by the traps, so the positive peak corresponds to the electron traps and the negative peak corresponds to the hole traps. Based on this, we attribute the negative peak in **Fig. 6a** to the hole trap H1.


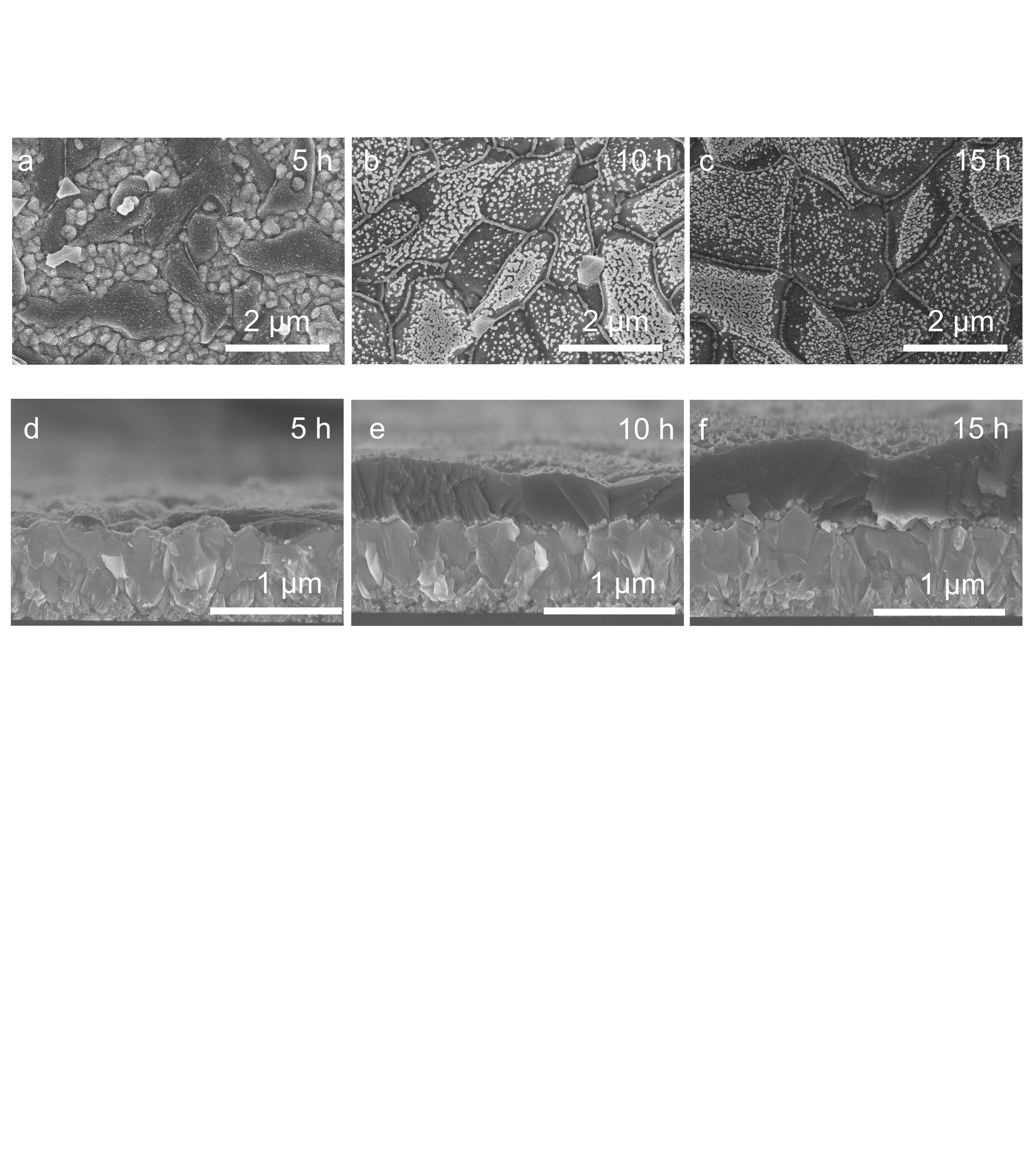


**Fig. S1** Top-view and cross-sectional SEM images of Sb_2_S_3_ precursor films deposited for different hydrothermal times: **a, d** 5 h, **b, e** 10 h, **c, f** 15 h

The hydrothermal deposition time was varied (5, 10, 15 h) to control the precursor thickness, and 10 h was determined as the optimal condition for achieving high-quality films under CSA (**Fig. S1**), as evidenced by the device performance statistics shown in **Fig. S2**. At a deposition time of 5 h (**Fig. S1a, d**), the resulting film was relatively thinwith incomplete surface coverage and sparse grain distribution, leading to insufficient light absorption and poor crystallinity. In contrast, when the deposition time was extended to 15 h (**Fig. S1c, f**), the film became excessively thick which compromises film uniformity and increases recombination losses. The 10 h deposition (**Fig. S1b, e**) produced a film with optimal thickness, dense and uniform grain structure, and well-defined grain boundaries, corresponding to the best device performance.

**
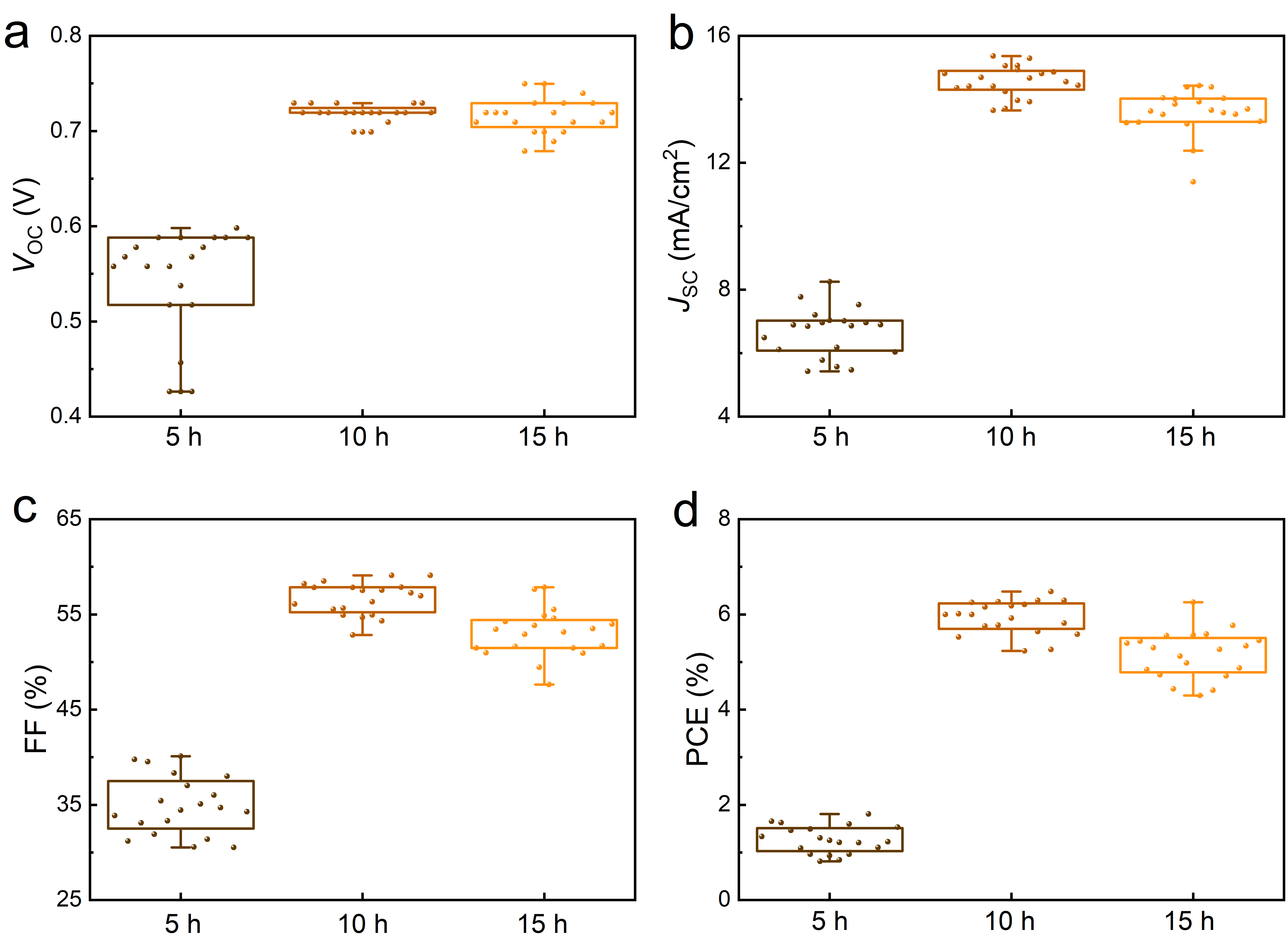
**

**Fig. S2** The performance parameters of Sb_2_S_3_ precursor films deposited for different hydrothermal times


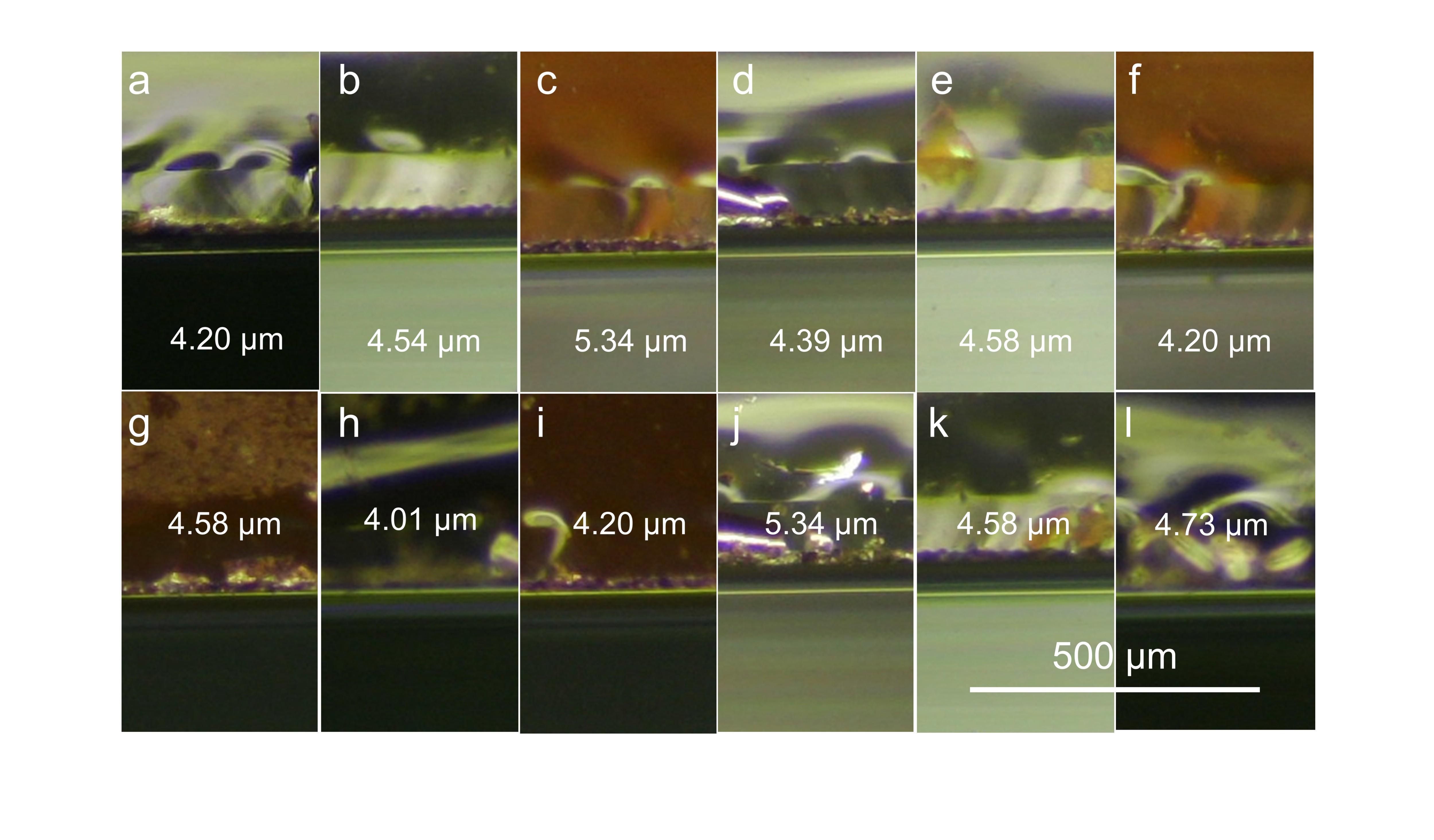


**Fig. S3** The results of multiple measurements of the gap size


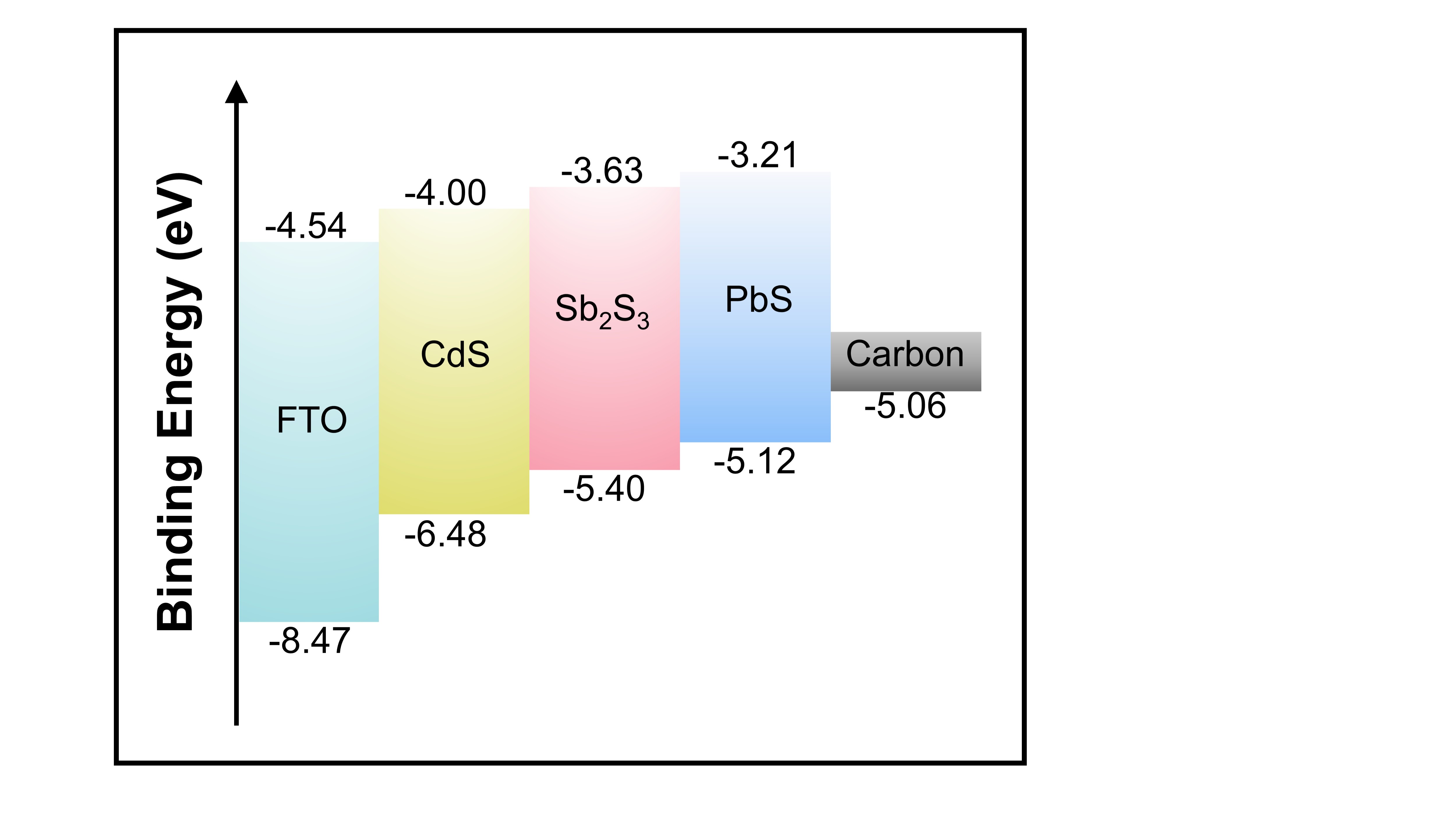


**Fig. S4** Energy level arrangement diagram


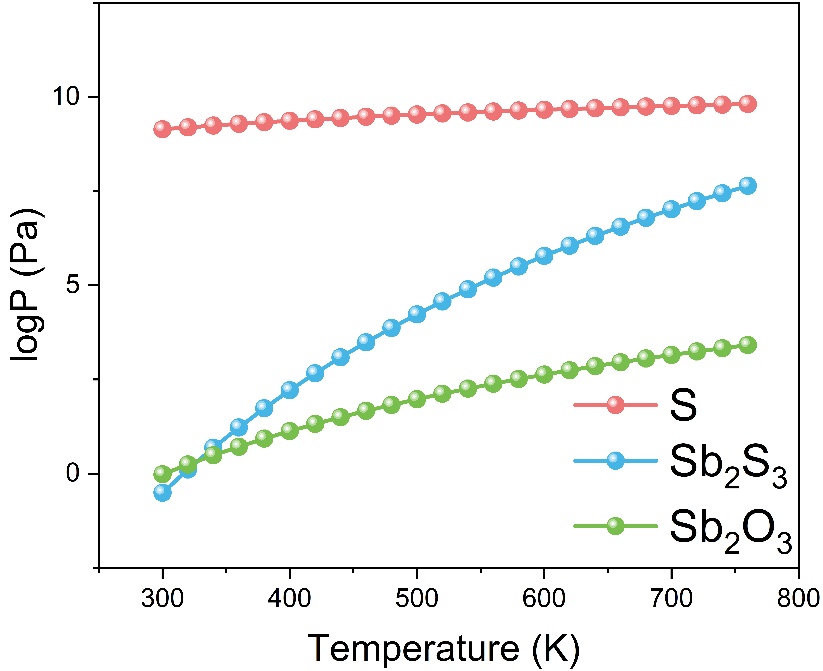


**Fig. S5** Saturated vapor pressure

Under the same temperature and pressure conditions, the higher the saturated vapor pressure of a substance, the stronger its volatility.


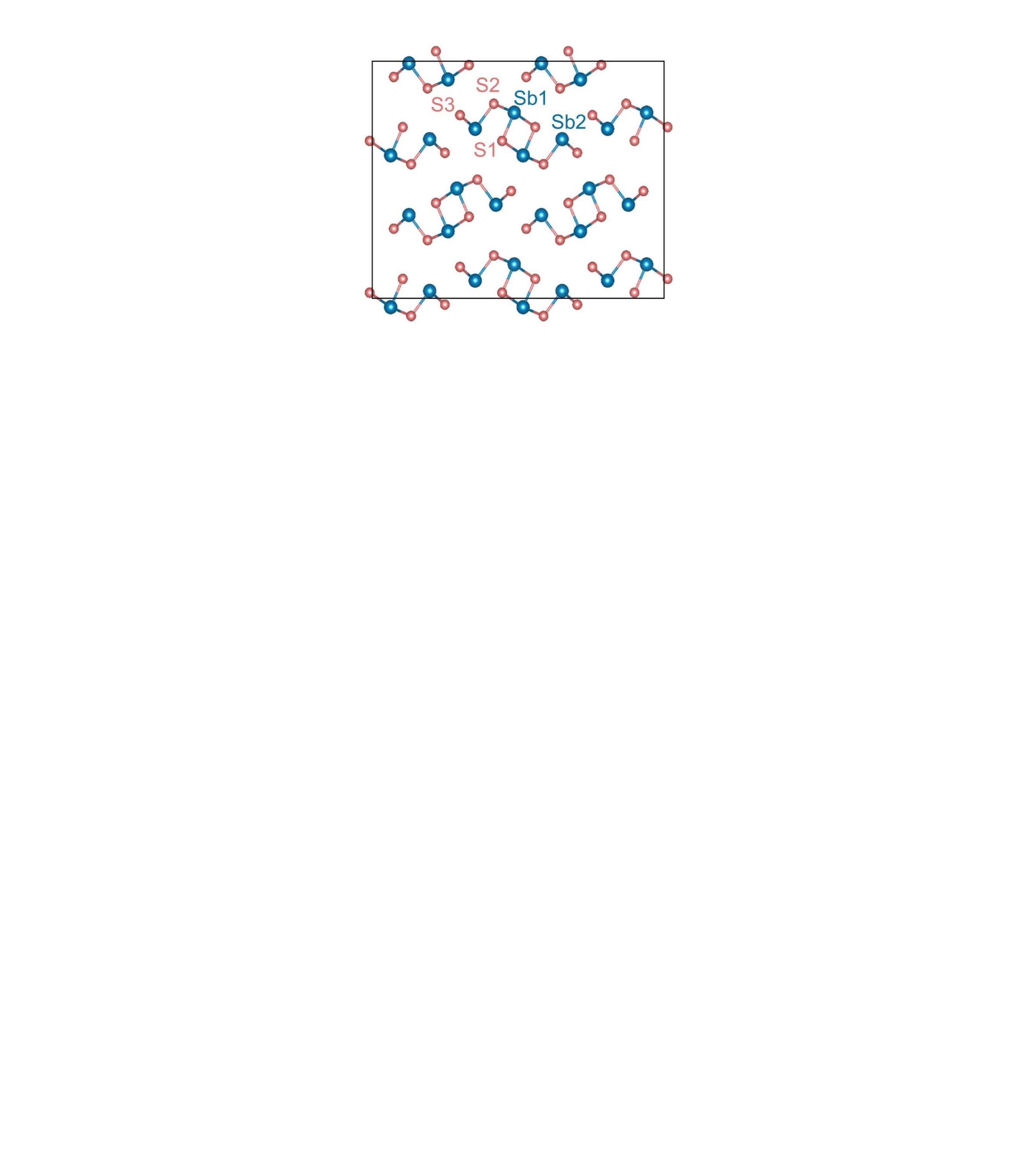


**Fig. S6** Crystal structure of Sb_2_S_3_


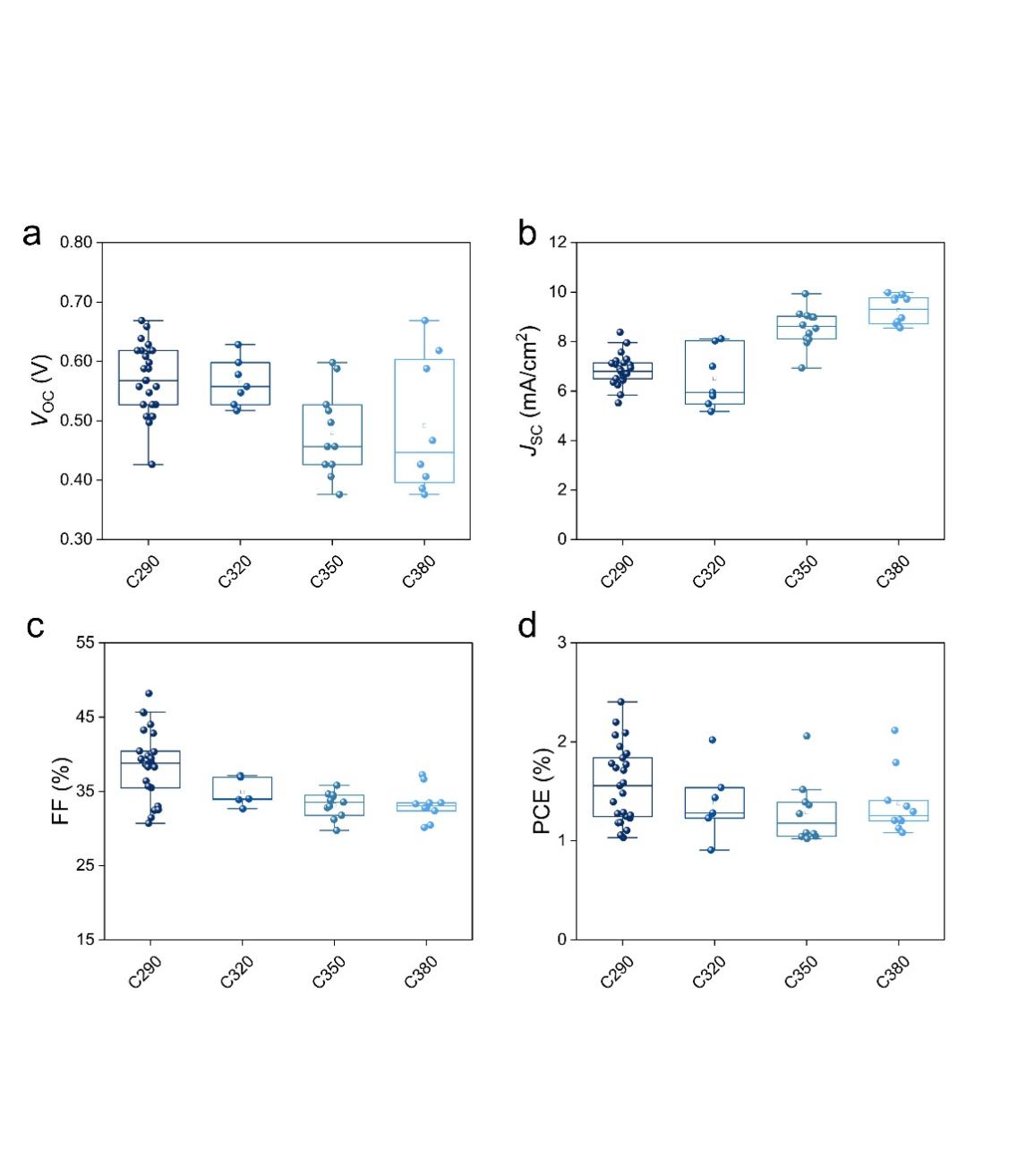


**Fig. S7** The performance parameters of C290-C380 devices


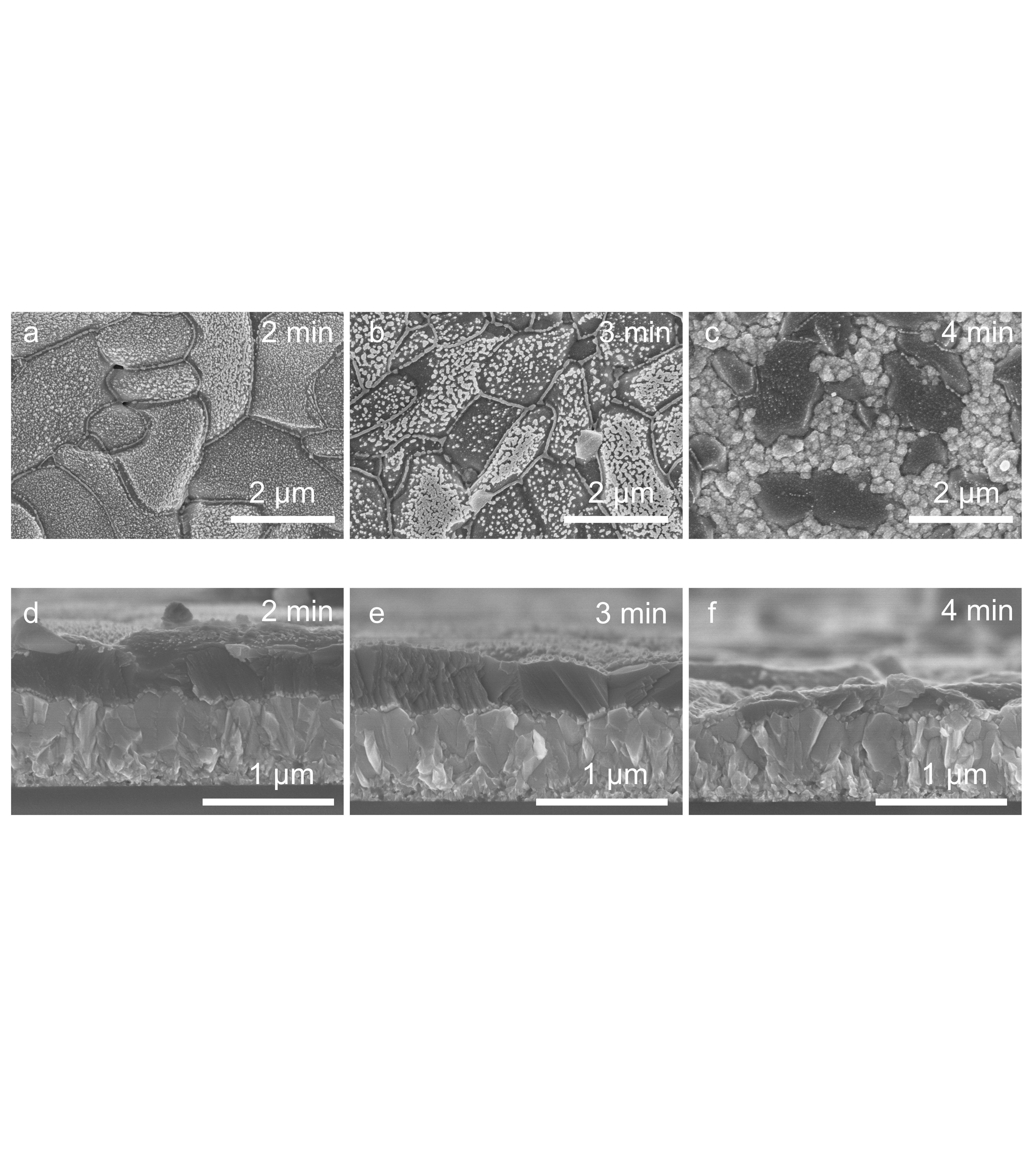


**Fig. S8** Top-view and cross-sectional SEM images of different annealing times under CSA: **a, d** 2 min, **b, e** 3 min, **c, f** 4 min

As shown in **Fig. S8**, when the annealing time was 2 min (**Fig. S8a, d**), only fine Sb_2_O_3_ particles were formed on the film surface, with a few holes present, and the cross-sectional structure was relatively loose; when the annealing time was extended to 3 min (**Fig. S8b, e**), the surface Sb_2_O_3_ particles were of moderate size and evenly distributed, with clear grain boundaries visible, and the cross-section showed a dense and continuous film, indicating good crystalline quality; when the annealing time was further increased to 4 min (**Fig. S8c, f**), the surface Sb_2_O_3_ particles overgrew and their size significantly increased, with some areas having blurred or even covered grain boundaries, and the cross-section showed discontinuous structures, indicating that excessive oxidation had damaged the film integrity.

**
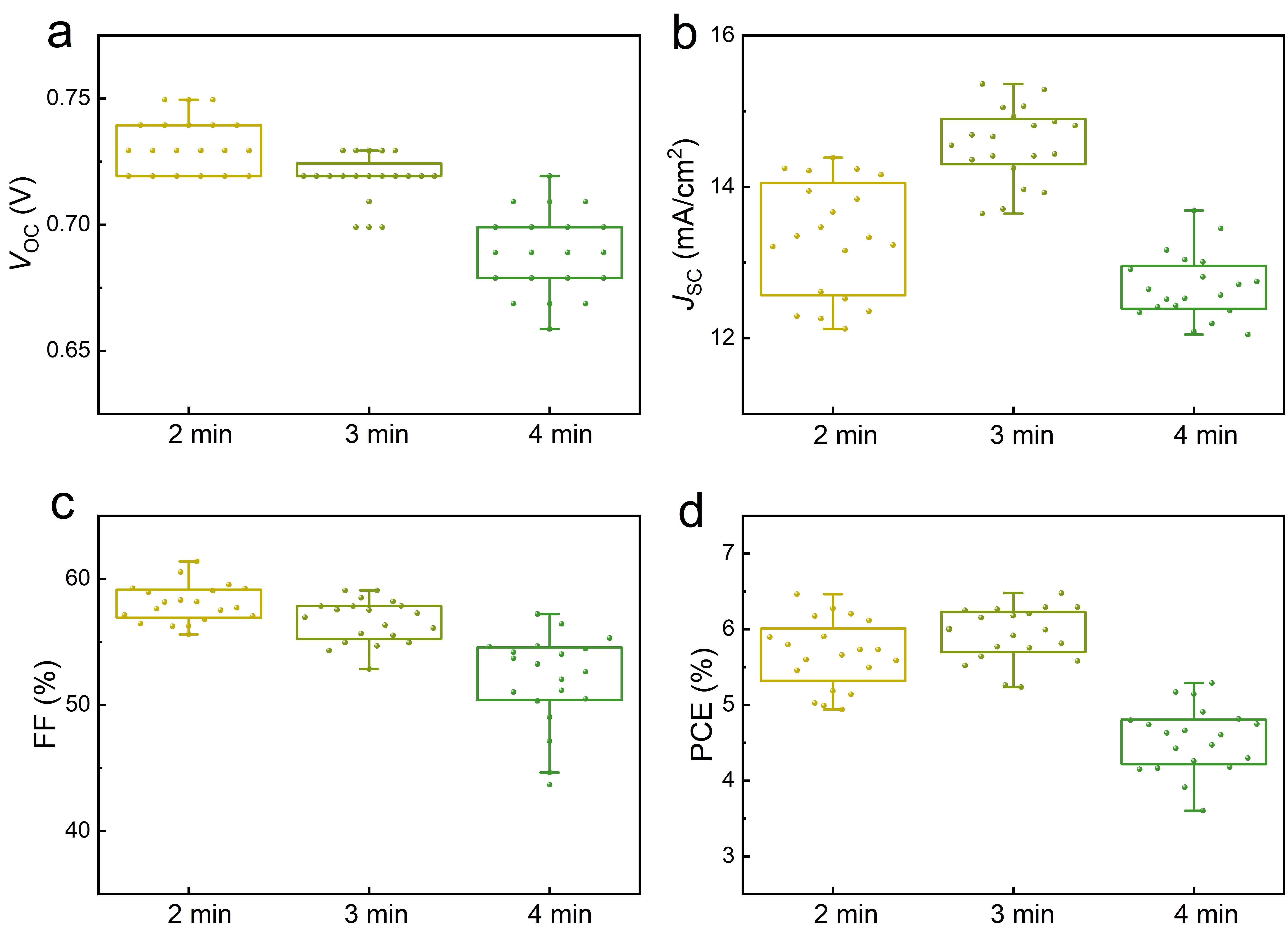
**

**Fig. S9** The performance parameters of different annealing times under CSA


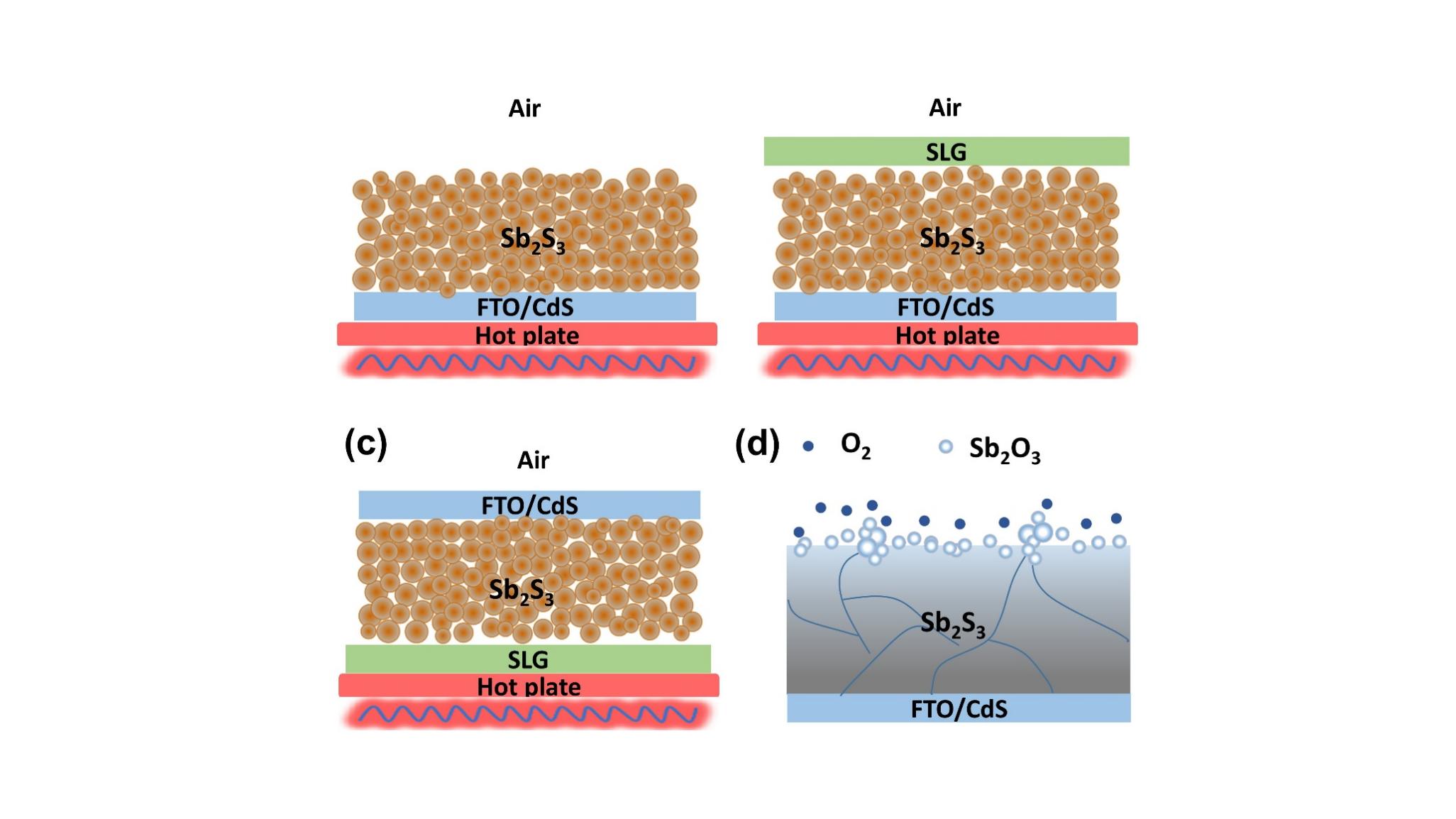


**Fig. S10** Schemes of Sb_2_S_3_ precursor films annealing in atmosphere using the up mode


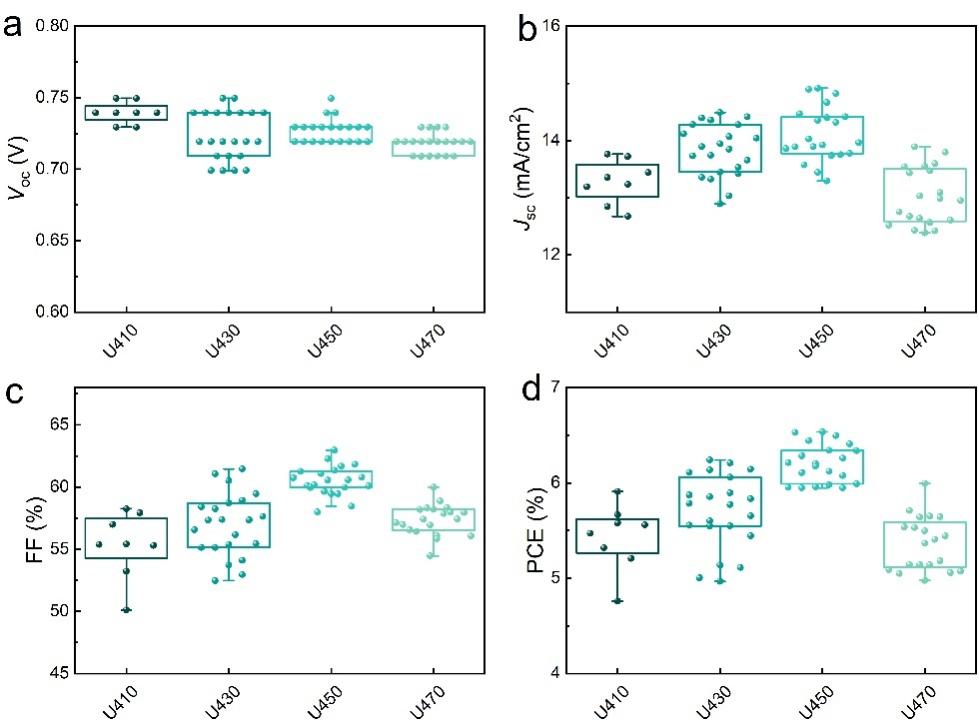


**Fig. S11** The performance parameters of devices with Sb_2_S_3_ annealed in the up mode

**
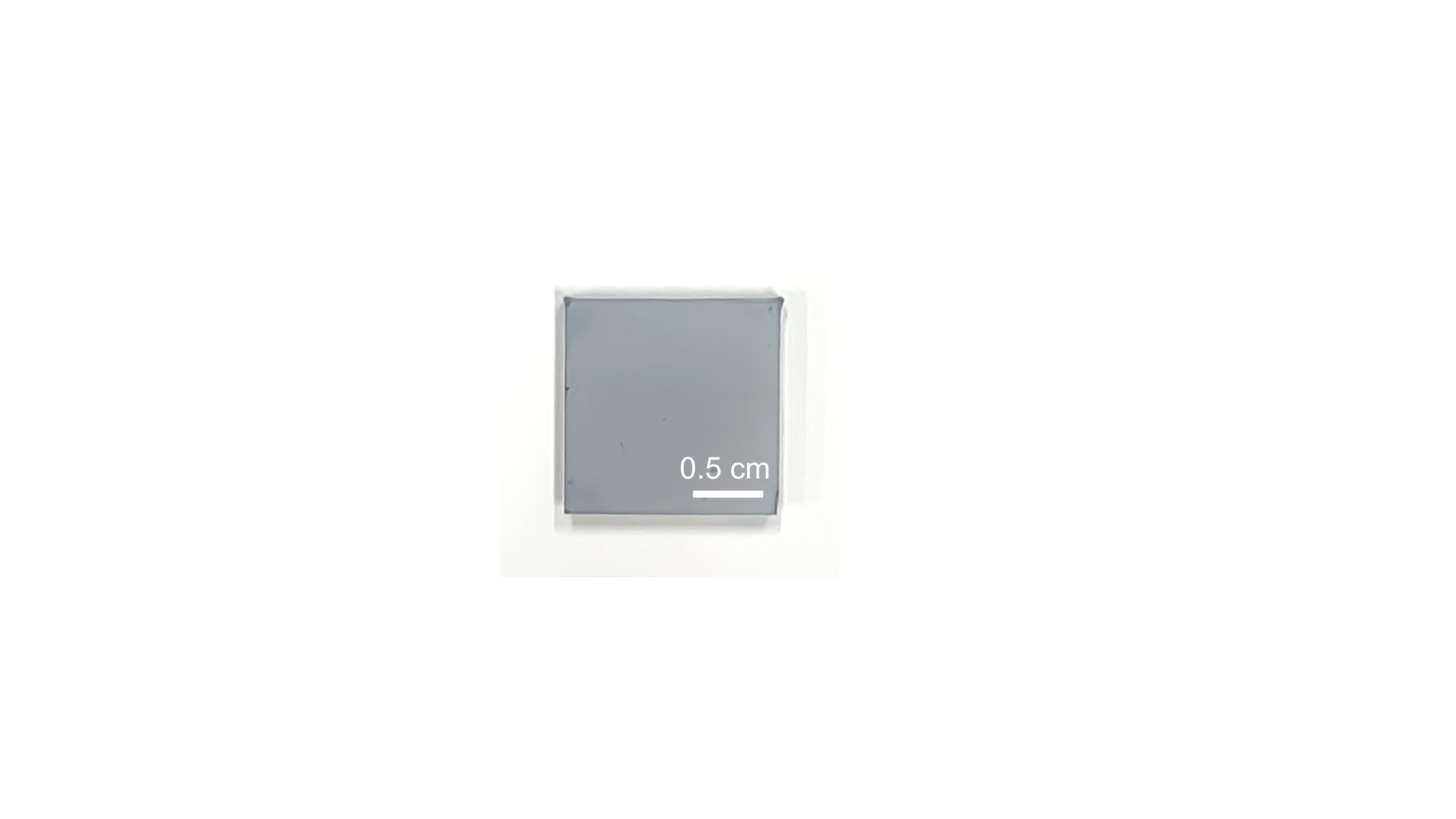
**

**Fig. S12** Macro digital photo of C450 after annealing

**
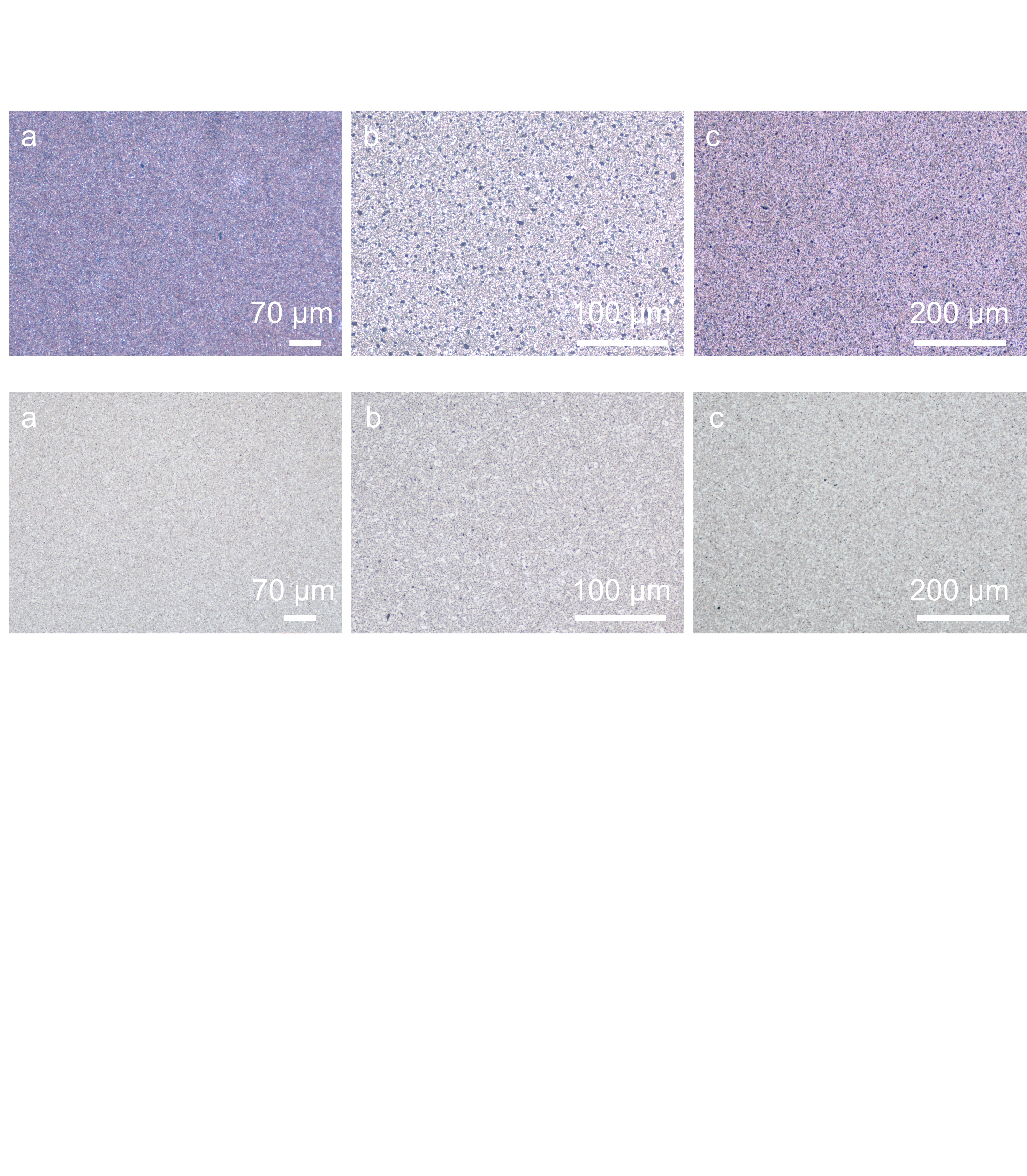
**

**Fig. S13** The different magnification larger-area optical microscope image of the C450 after annealing


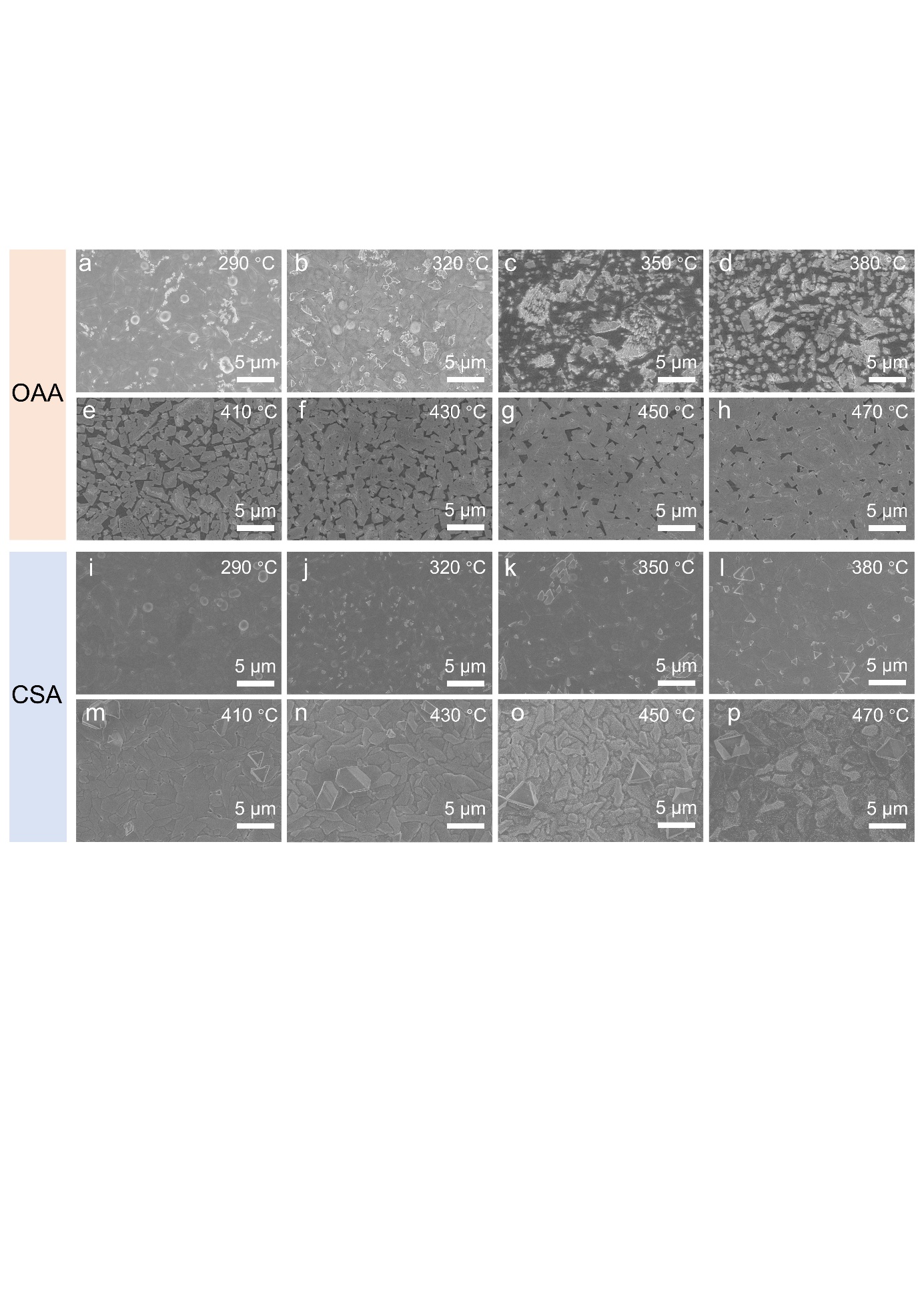


**Fig. S14** Low-magnification SEM images of **a-h** OAA and **i-p** CSA samples

**Figure S14** showed that the OAA sample exposed large areas of densely distributed micrometer-sized holes, as well as large-sized Sb_2_O_3_ blocks coverings, indicating that the oxidation damage had extended from local areas to the entire film surface. In contrast, the CSA sample still presented a completely dense, hole-free, and non-peeling flat surface at the same magnification, with only Sb_2_O_3_ nano-belts visible at the grain boundaries.


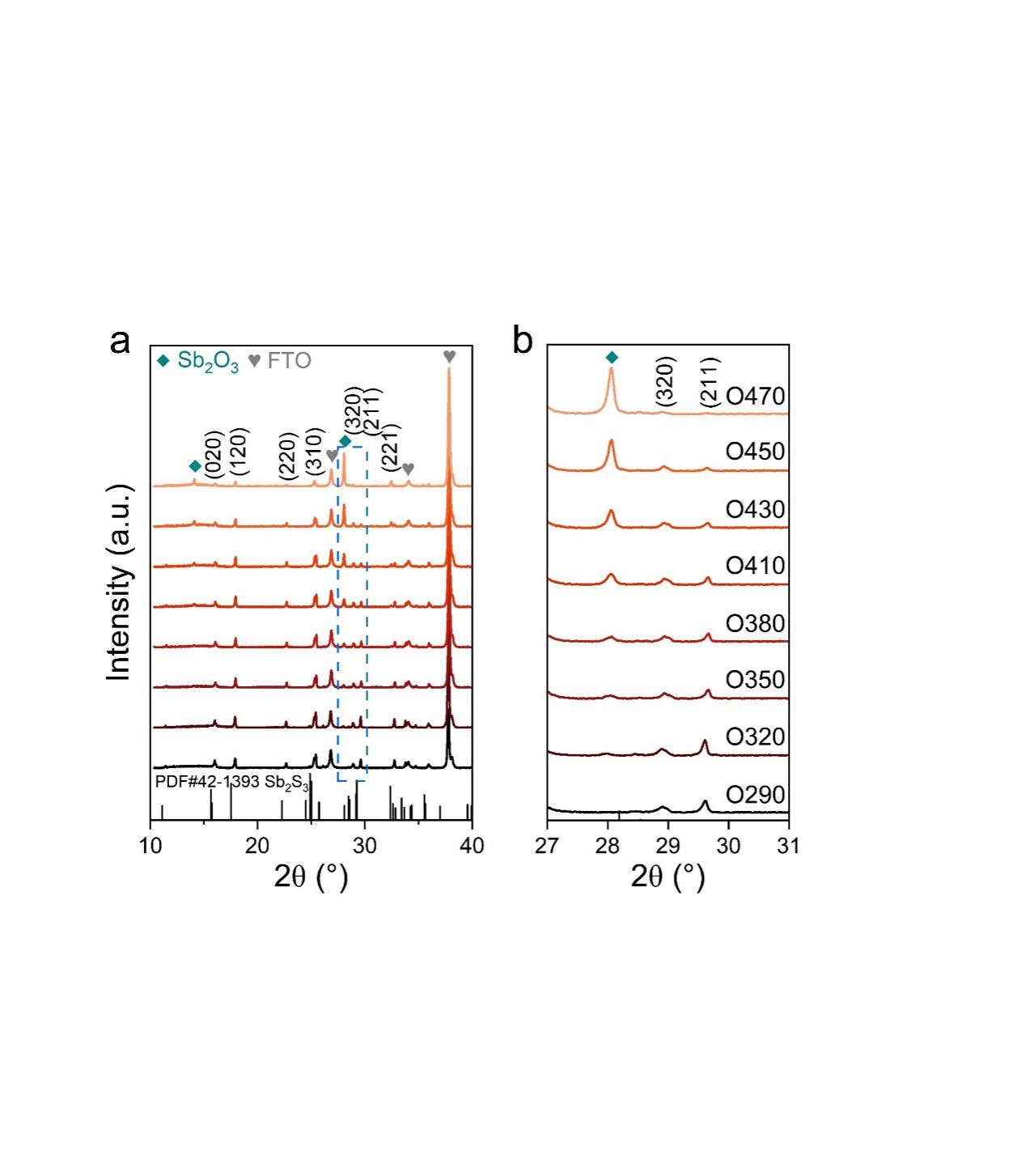


**Fig. S15** **a** XRD patterns of sample O290-O470; **b** Magnified view of XRD pattern in the range of 27-31^o^

**Figure S15** shows the XRD patterns of OAA samples treated at different temperatures. The crystallinity shows a certain increasing trend with temperature.


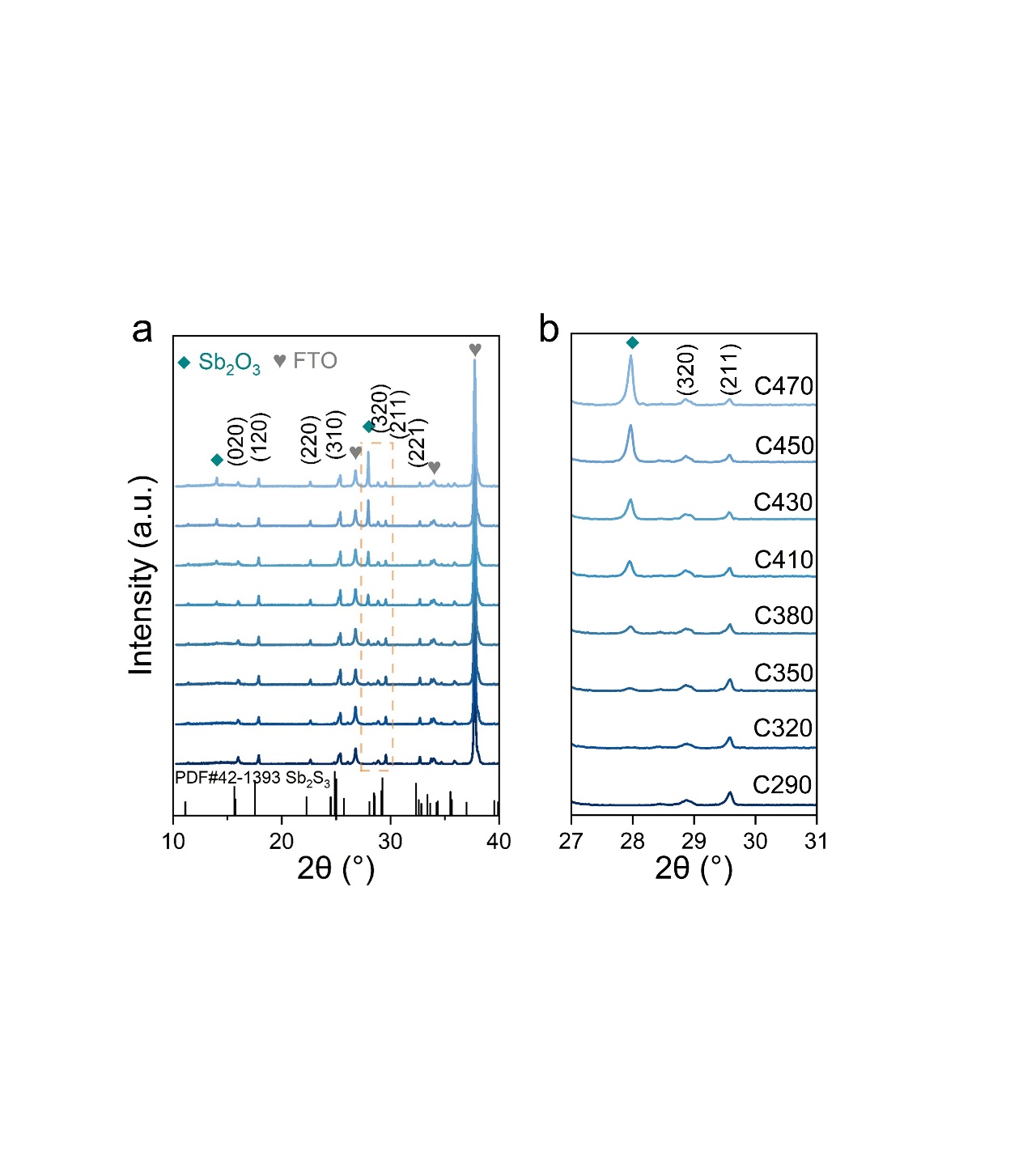


**Fig. S16** **a** XRD patterns of sample C290-C470; **b** Magnified view of XRD pattern in the range of 27-31^o^

**Figure S16** shows the XRD patterns of samples under the CSA strategy within the range of 290 to 470 °C. The diffraction peaks are sharper, indicating a significant optimization of crystallinity.


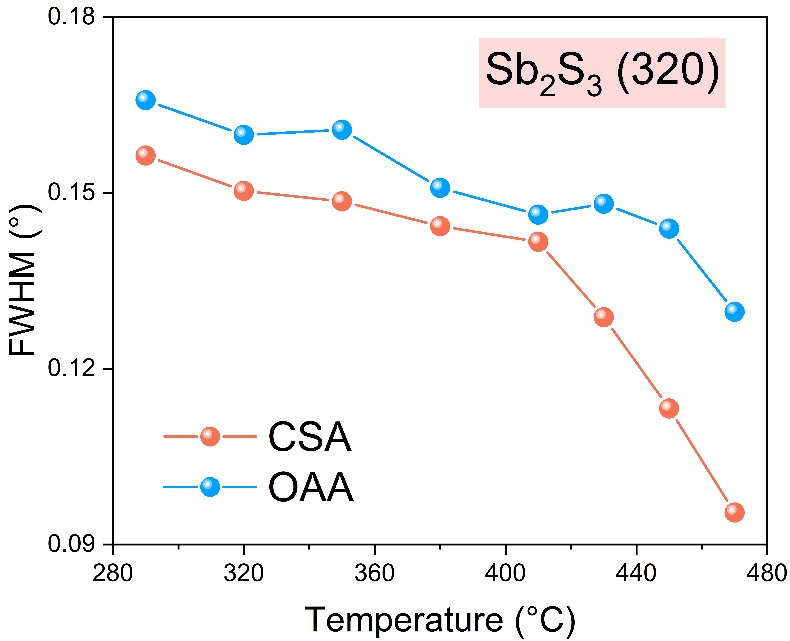


**Fig. S17** FWHM plots of samples for the Sb_2_S_3_ (320) crystal plane

Compared with the same characteristic crystal plane, the FWHM value of CSA samples is smaller. Since FWHM is negatively correlated with grain size and lattice order (narrower peaks correspond to better crystallinity), this result directly supports that the crystallinity of CSA samples is significantly better than that of OAA samples.


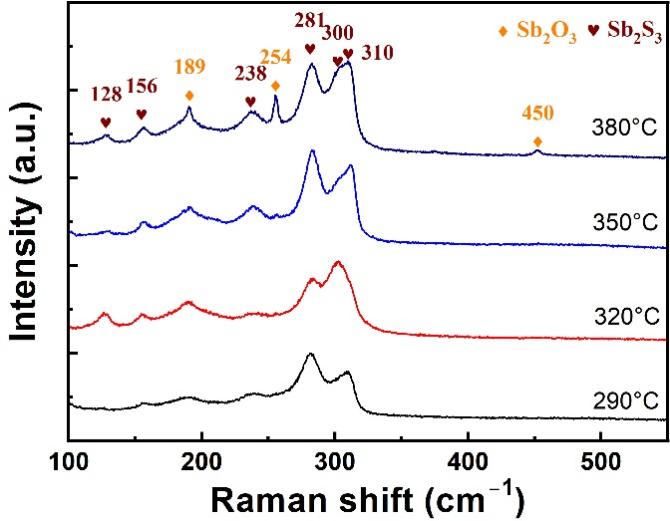


**Fig. S18** Raman spectra of O290-O380 samples

Raman spectra (**Fig. S18**) show that peaks near 128, 156, 238, 300, and 310 cm^-1^ are characteristic peaks of Sb_2_S_3_, and peaks near 189, 254, and 450 cm^-1^ are characteristic peaks of Sb_2_O_3_. As the annealing temperature increased from 290 °C to 380 °C, the intensity of the characteristic peaks of Sb_2_S_3_ increased and the peak shapes became sharper, indicating that the crystallinity of Sb_2_S_3_ improved with increasing temperature. Simultaneously, the intensity of the characteristic peaks of Sb_2_O_3_ gradually increased, suggesting that the increase in temperature promoted the oxidation of Sb_2_S_3_ and increased the formation of Sb_2_O_3_. Although the increase in temperature improved the crystallinity of Sb_2_S_3_, it also exacerbated the oxidation side reaction, leading to excessive accumulation of the inactive Sb_2_O_3_ phase.

**
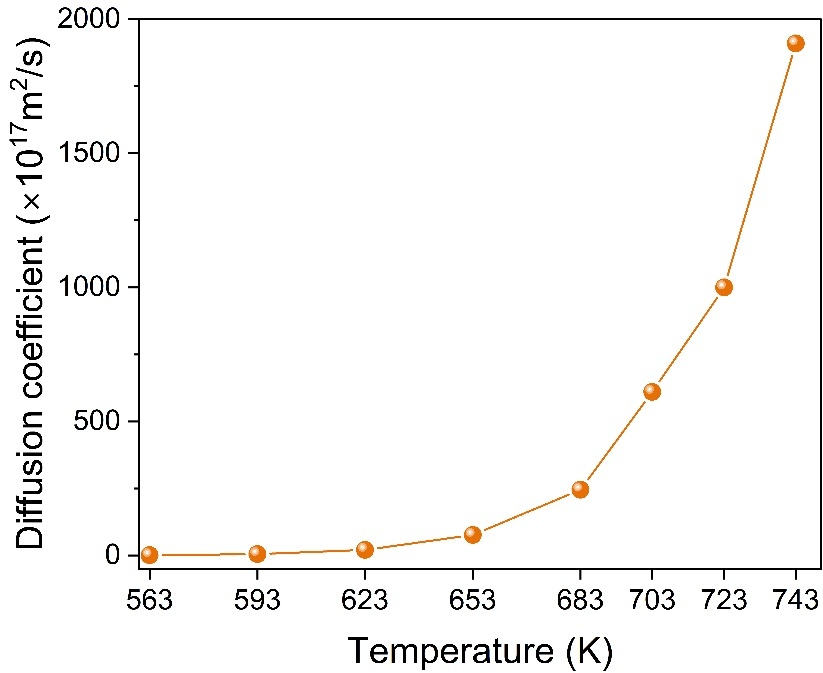
**

**Fig. S19** Diffusion coefficient (*D*) at different temperatures


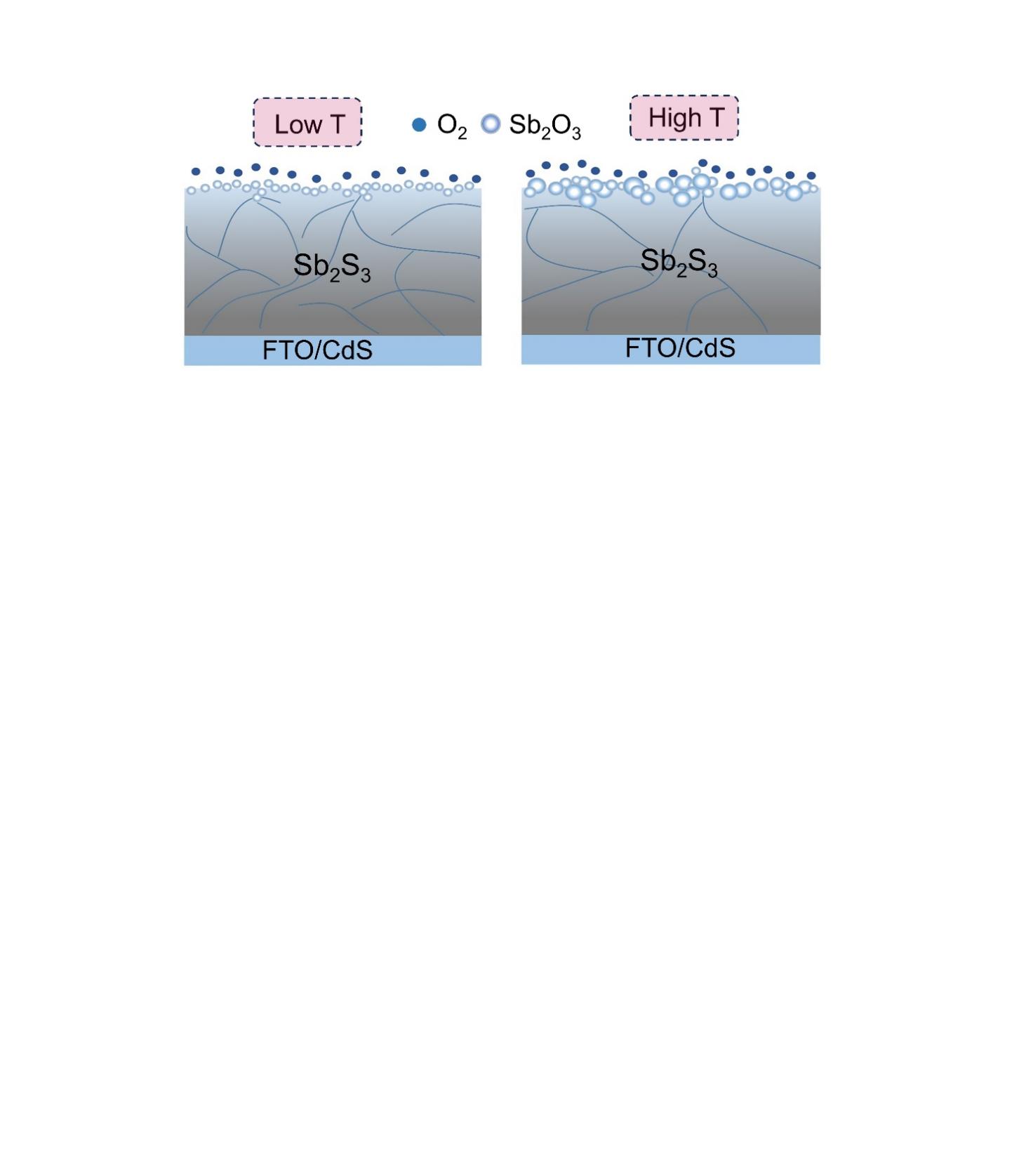


**Fig. S20** Crystallization schematic diagrams of Sb_2_S_3_ under CSA at low-temperature and high-temperature


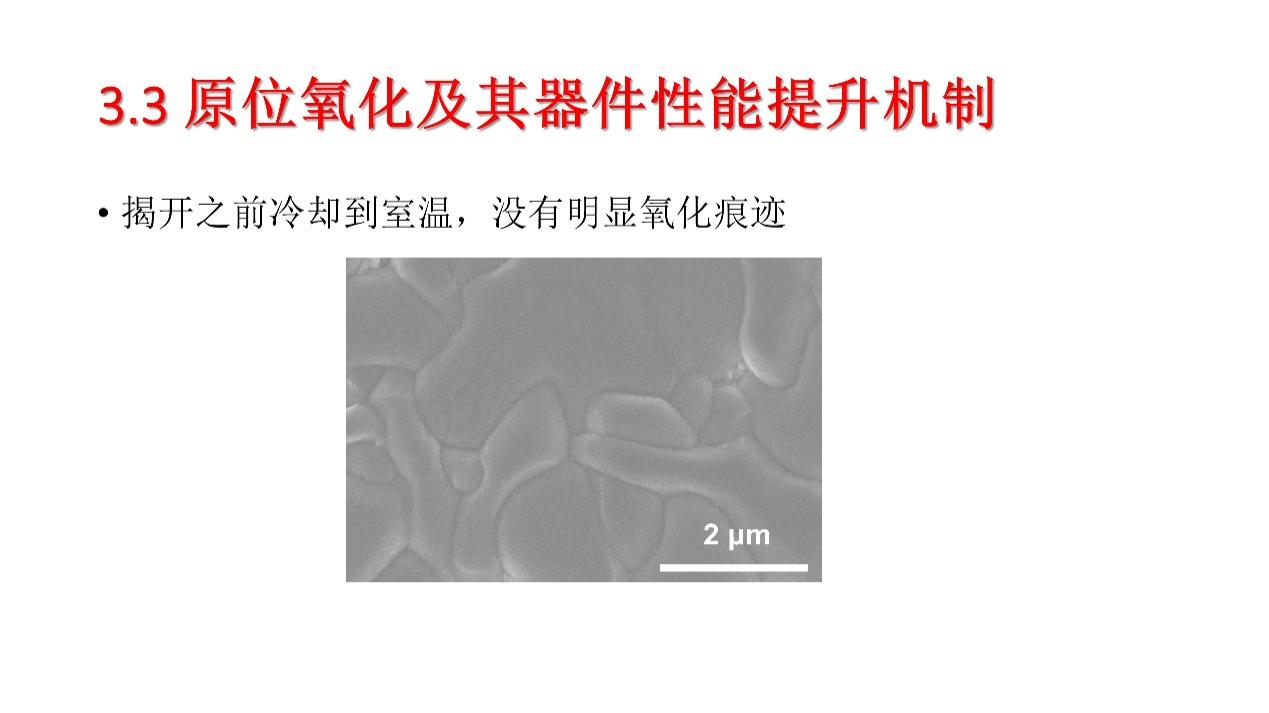


**Fig. S21** SEM image of the CSA sample that has naturally cooled to room temperature

There were no significant oxidation signs on the film surface.


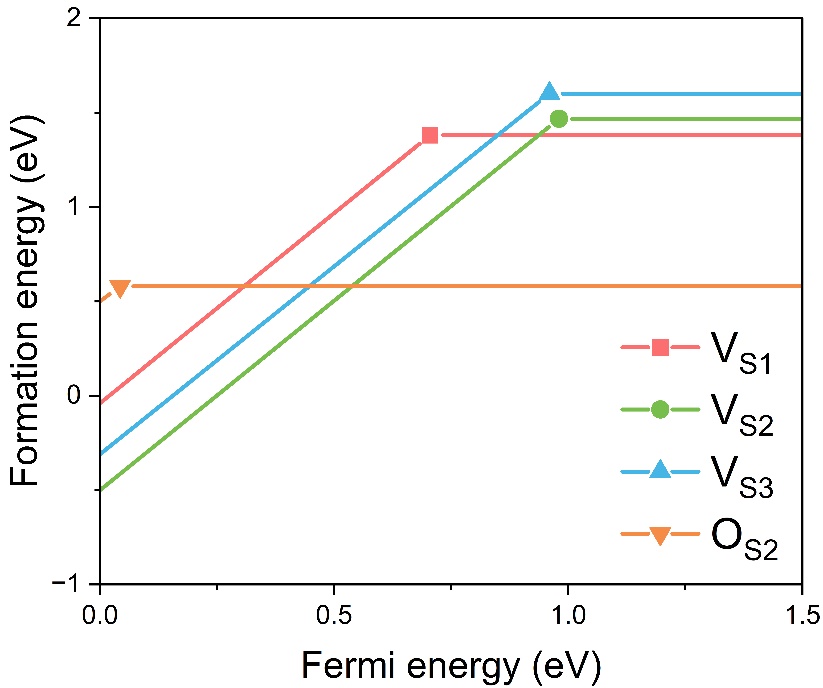


**Fig. S22** DFT calculation of defects in Sb_2_S_3_

DFT calculations confirm that among all intrinsic point defects in Sb_2_S_3_, the formation energy of V_S2_ is the lowest. The formation of O_S2_ defects is lower than that of V_S2_ when Fermi energy is larger than 0.5 eV.


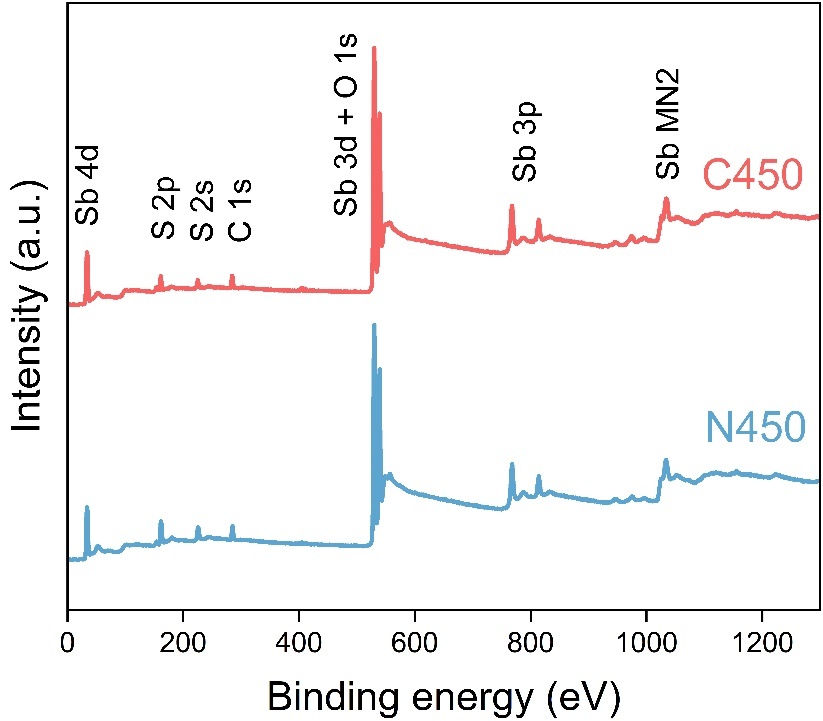


**Fig. S23** XPS survey spectra of C450 and N450 samples


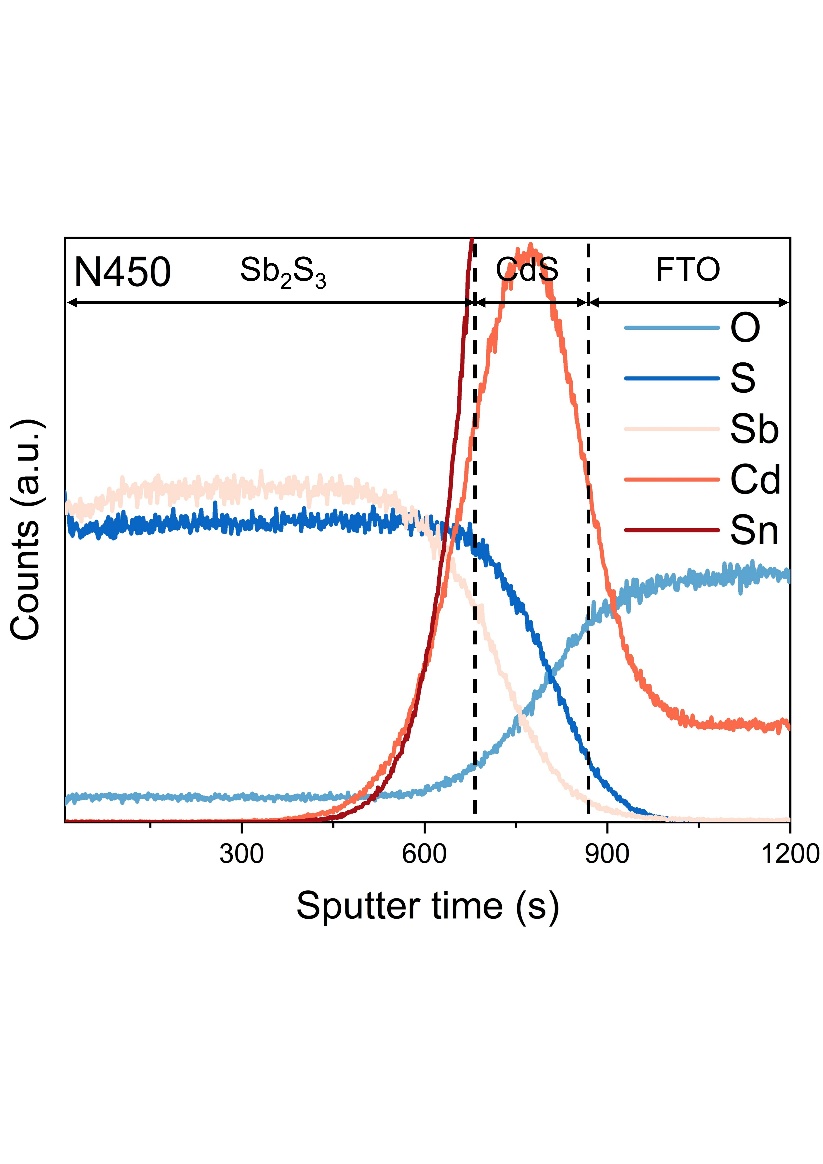


**Fig. S24** SIMS intensity of N450 samples

The counts of Sb and S in N450 samples remained basically constant with the etching time, indicating that the Sb_2_S_3_ composition was uniformly distributed in the bulk.


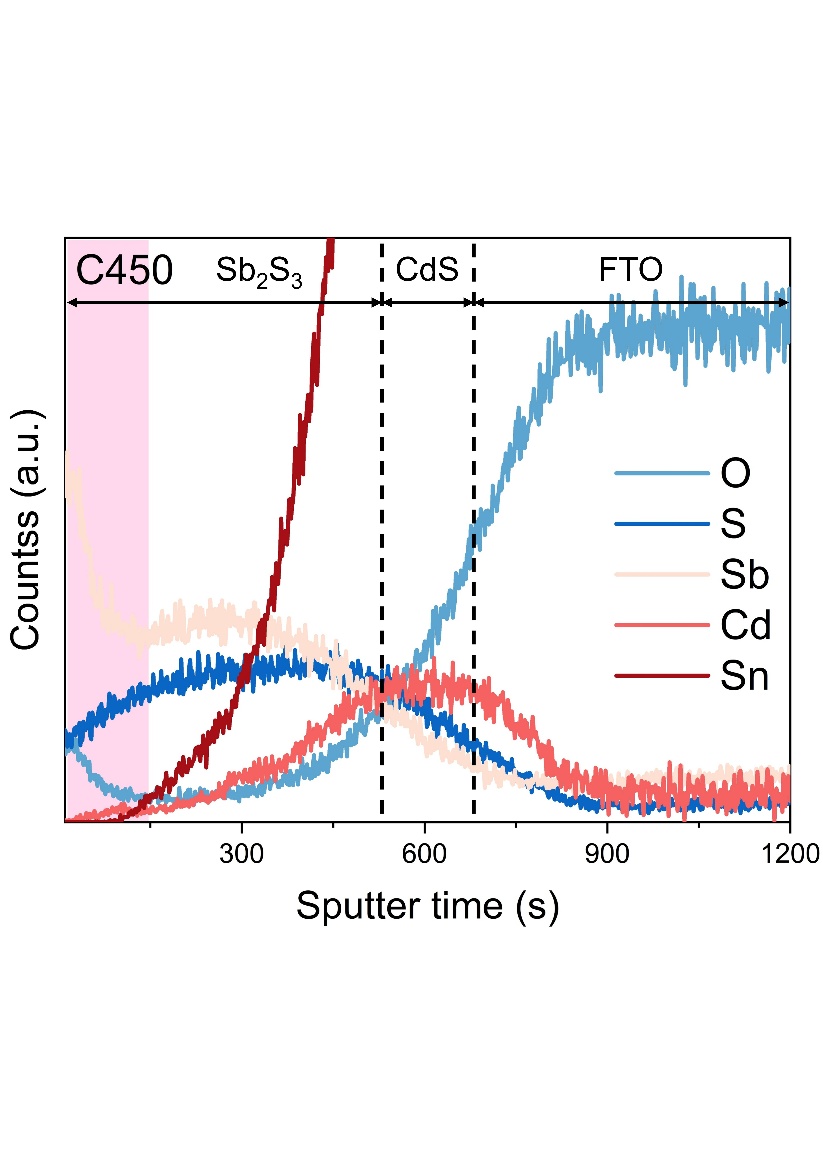


**Fig. S25** SIMS intensity of C450 samples

Due to the oxidation of Sb_2_S_3_, the surface of C450 film was uneven, causing some Cd and Sn to be exposed earlier. The surface layer (sputtering time ≤ 140 s) of C450 showed a gradient change in Sb, O, and S.


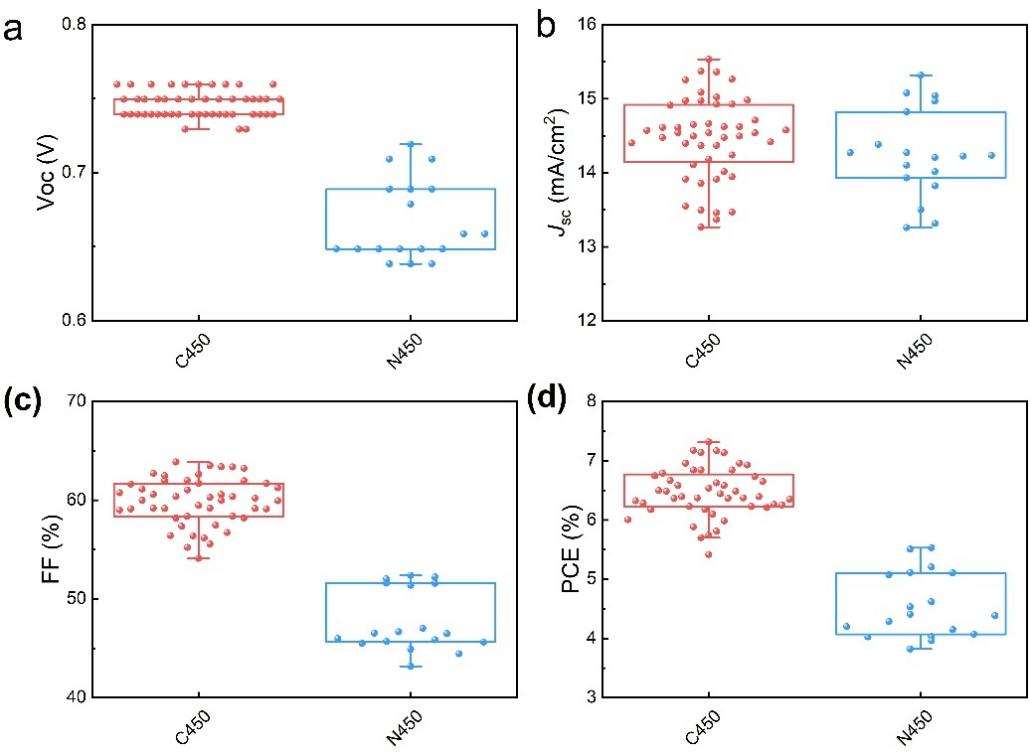


**Fig. S26** Statistical device parameters of the C450 and N450 solar cells


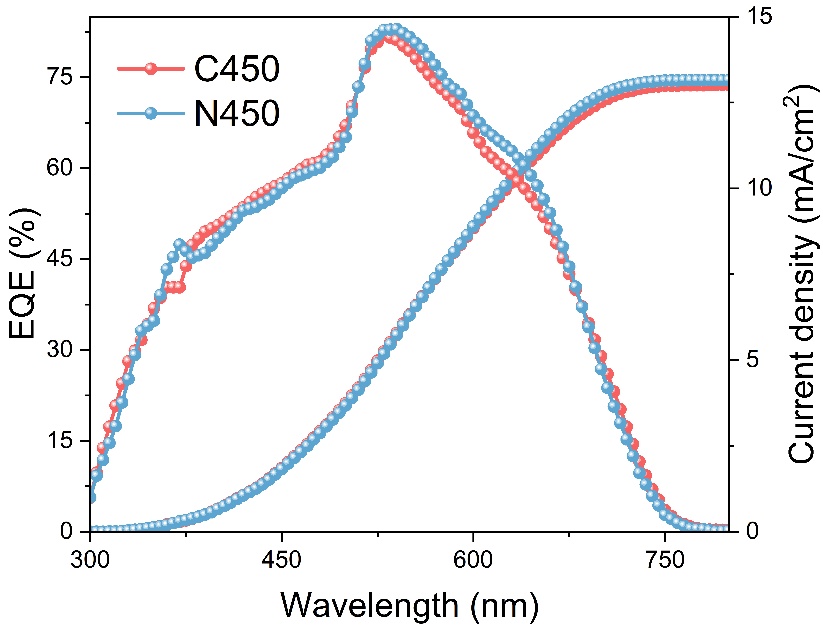


**Fig. S27** EQE spectra of C450 and N450 devices


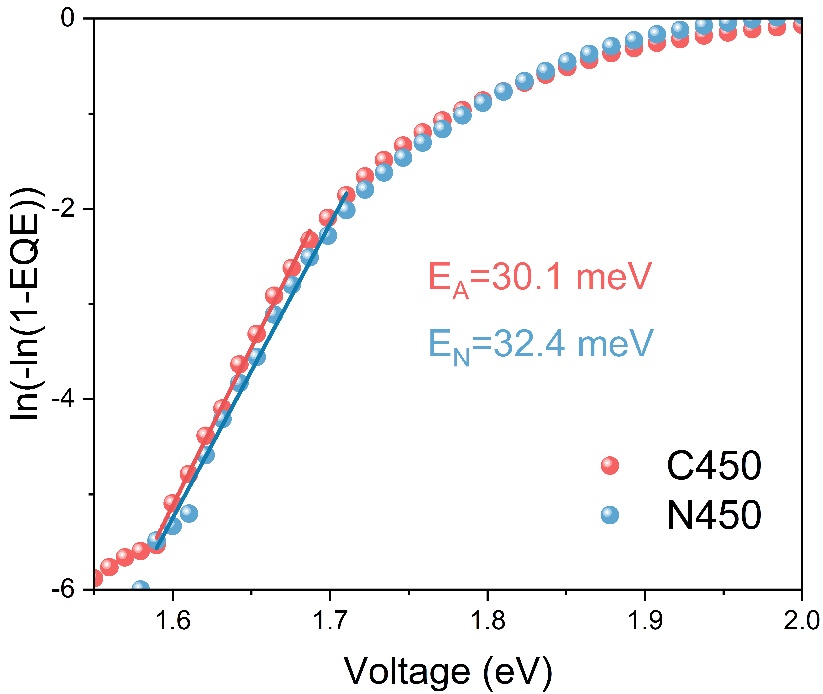


**Fig. S28** Urbach energy of the C450 and N450 devices

**
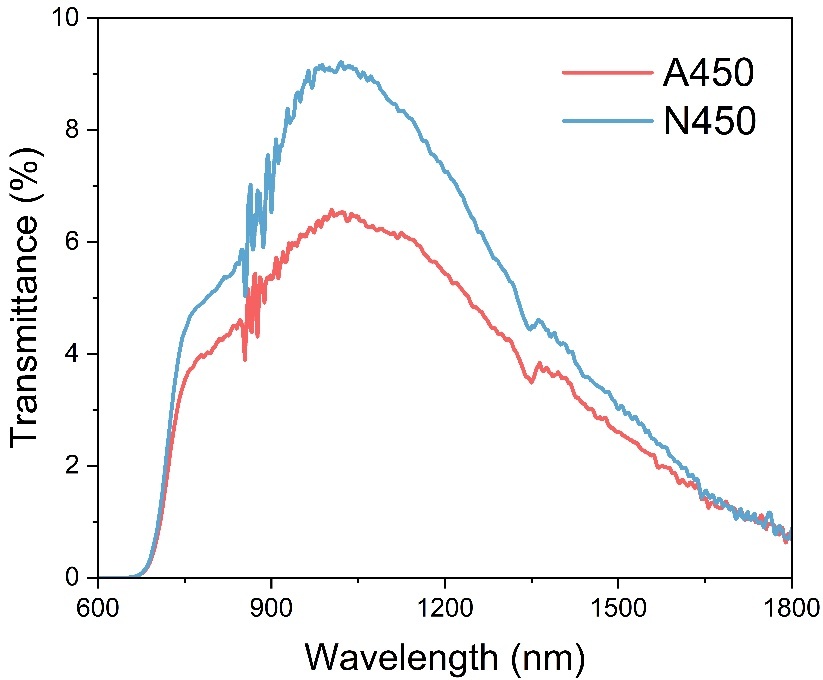
**

**Fig. S29** Transmittance of the C450 and N450 sample


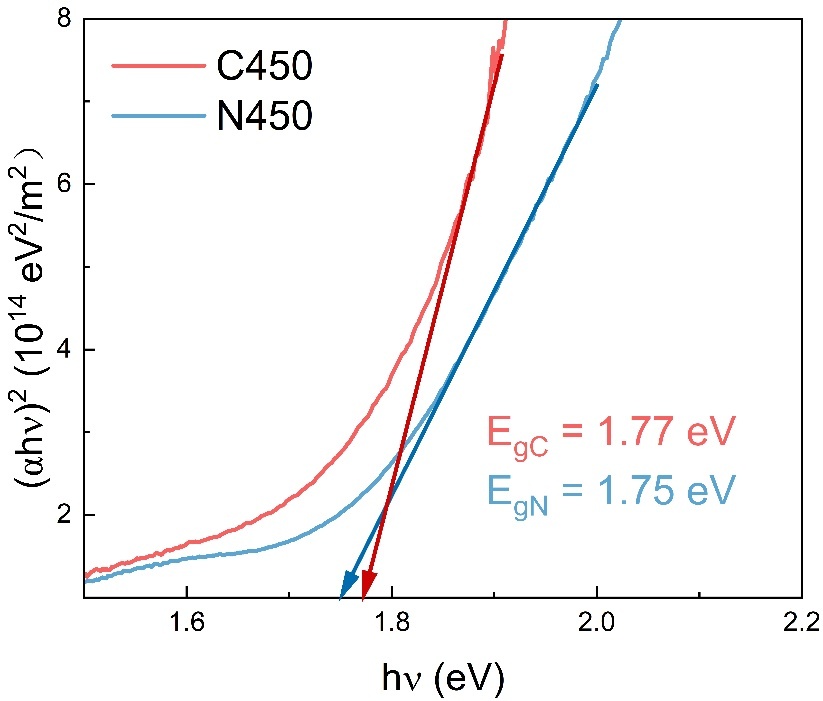


**Fig. S30** Tauc plot of the C450 and N450 sample


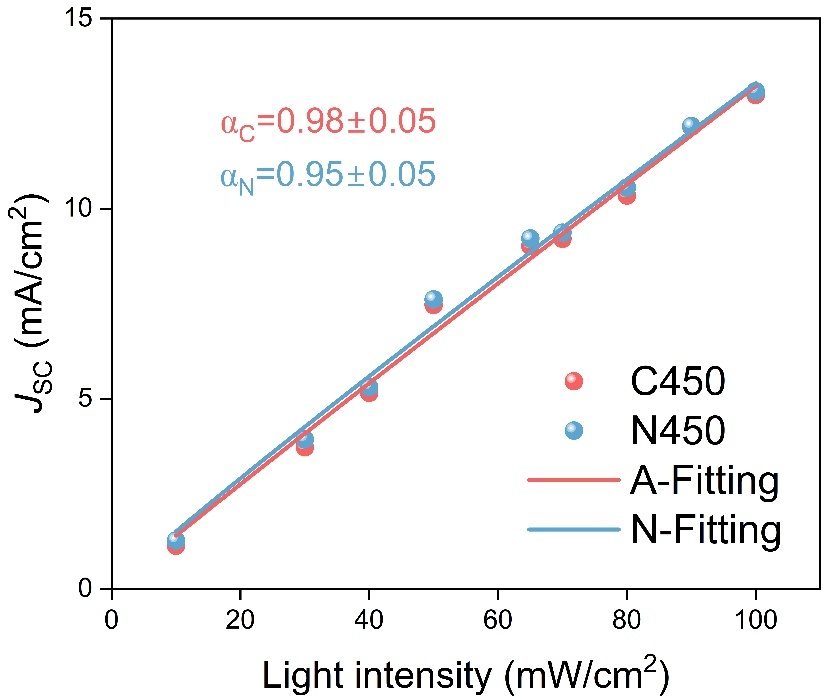


**Fig. S31** Short circuit current density as a function of light intensity of C450 and N450 devices


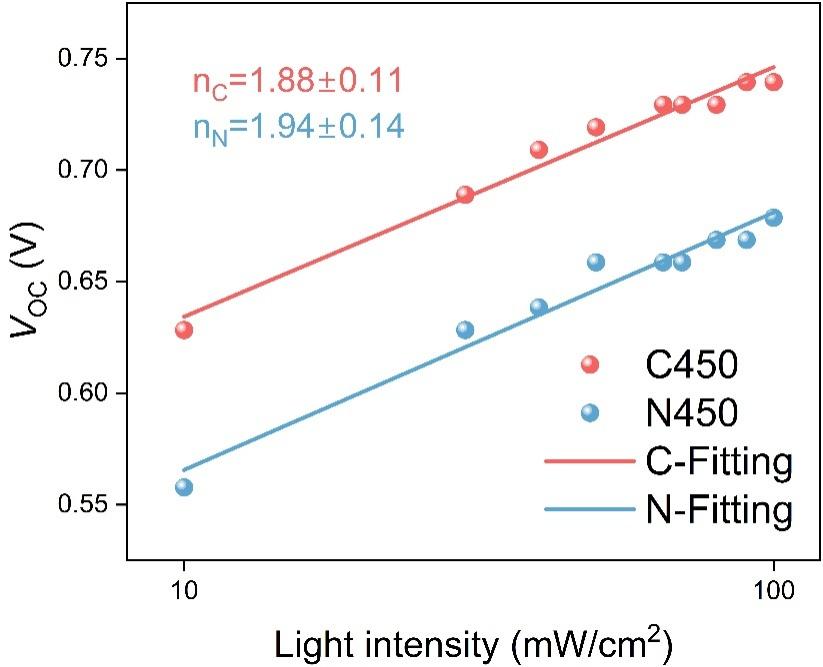


**Fig. S32** Open circuit voltage as a function of light intensity of C450 and N450 devices


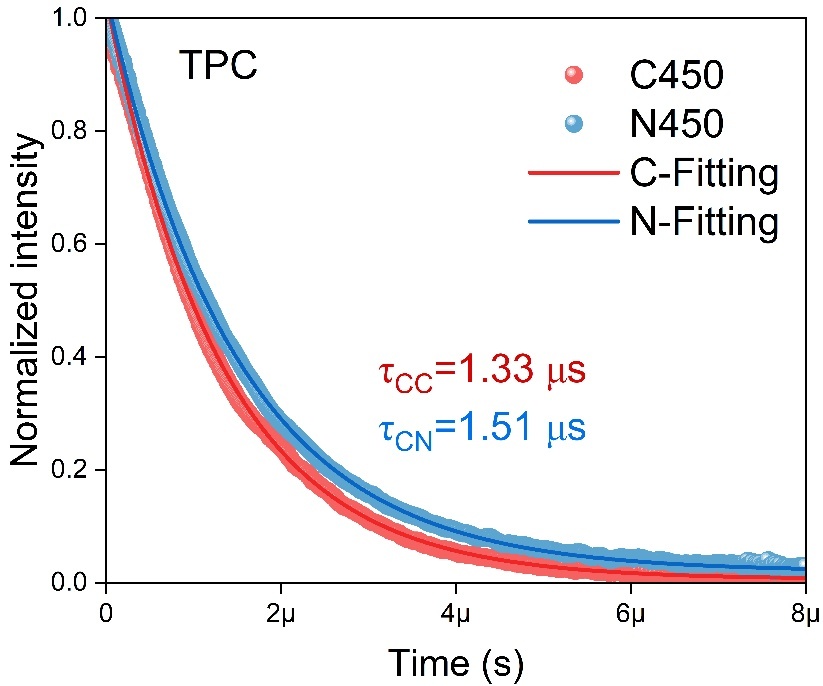


**Fig. S33** Transient photocurrent (TPC) of C450 and N450 devices


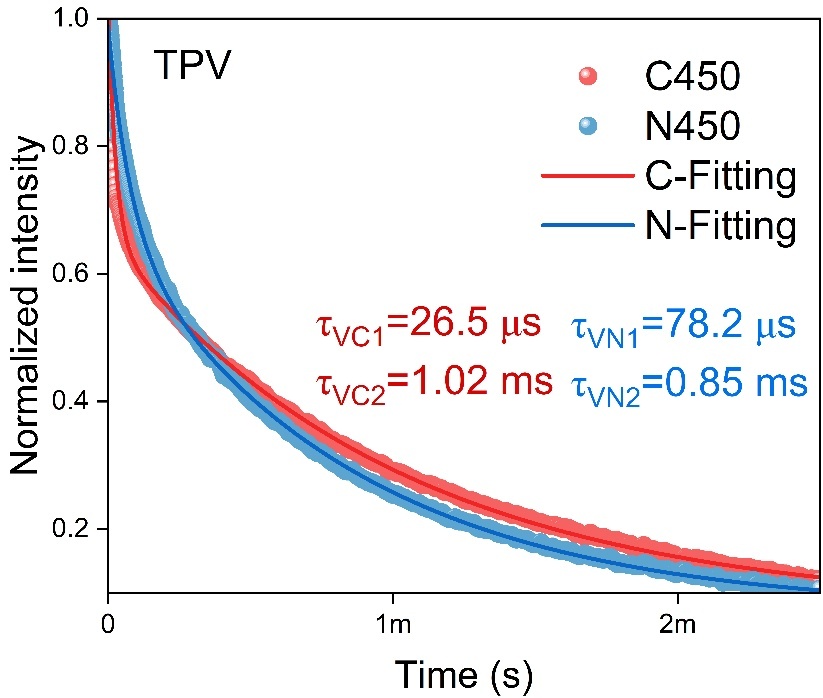


**Fig. S34** Transient photovoltage (TPV) of C450 and N450 devices


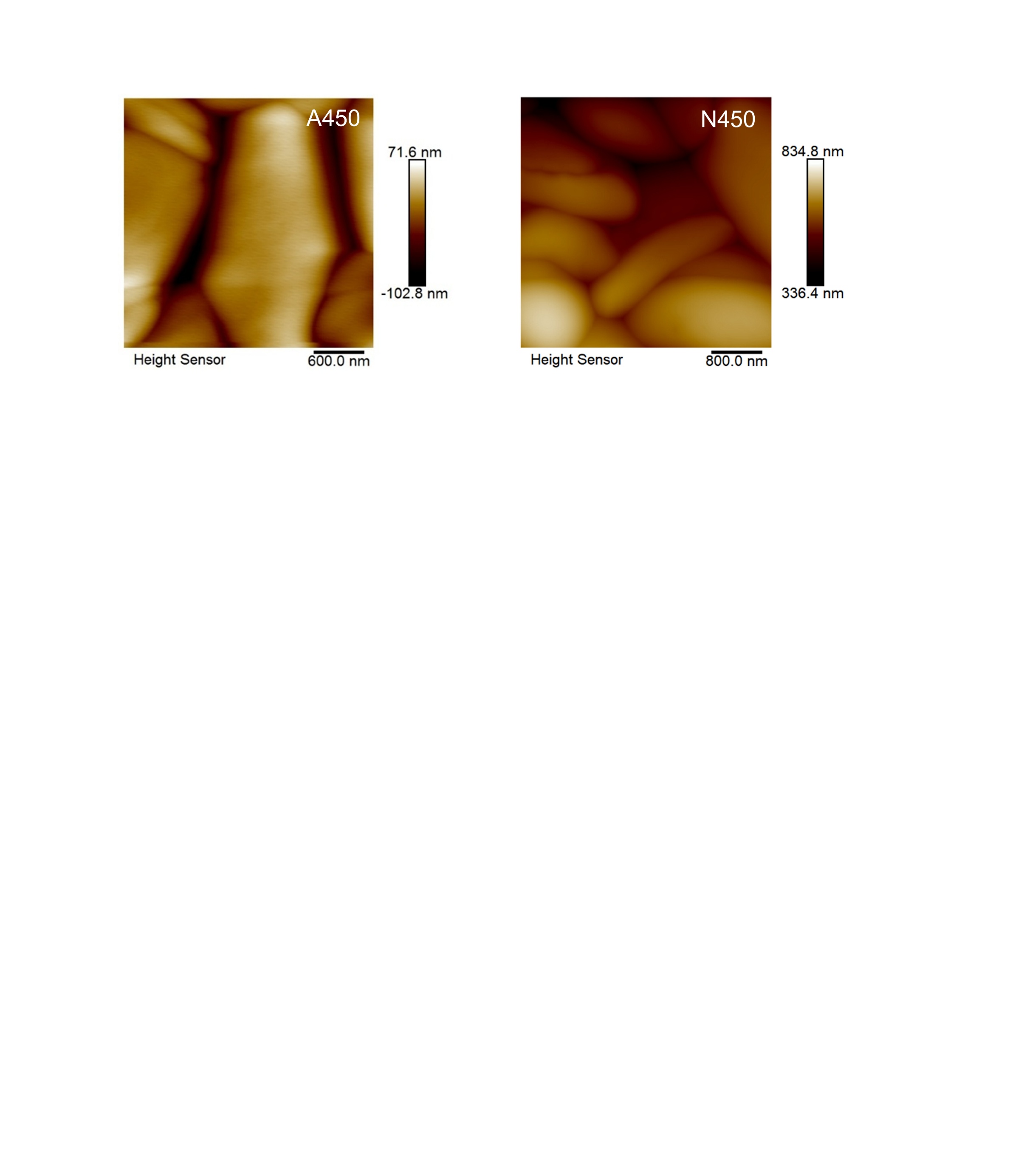


**Fig. S35** AFM image of N450 devices


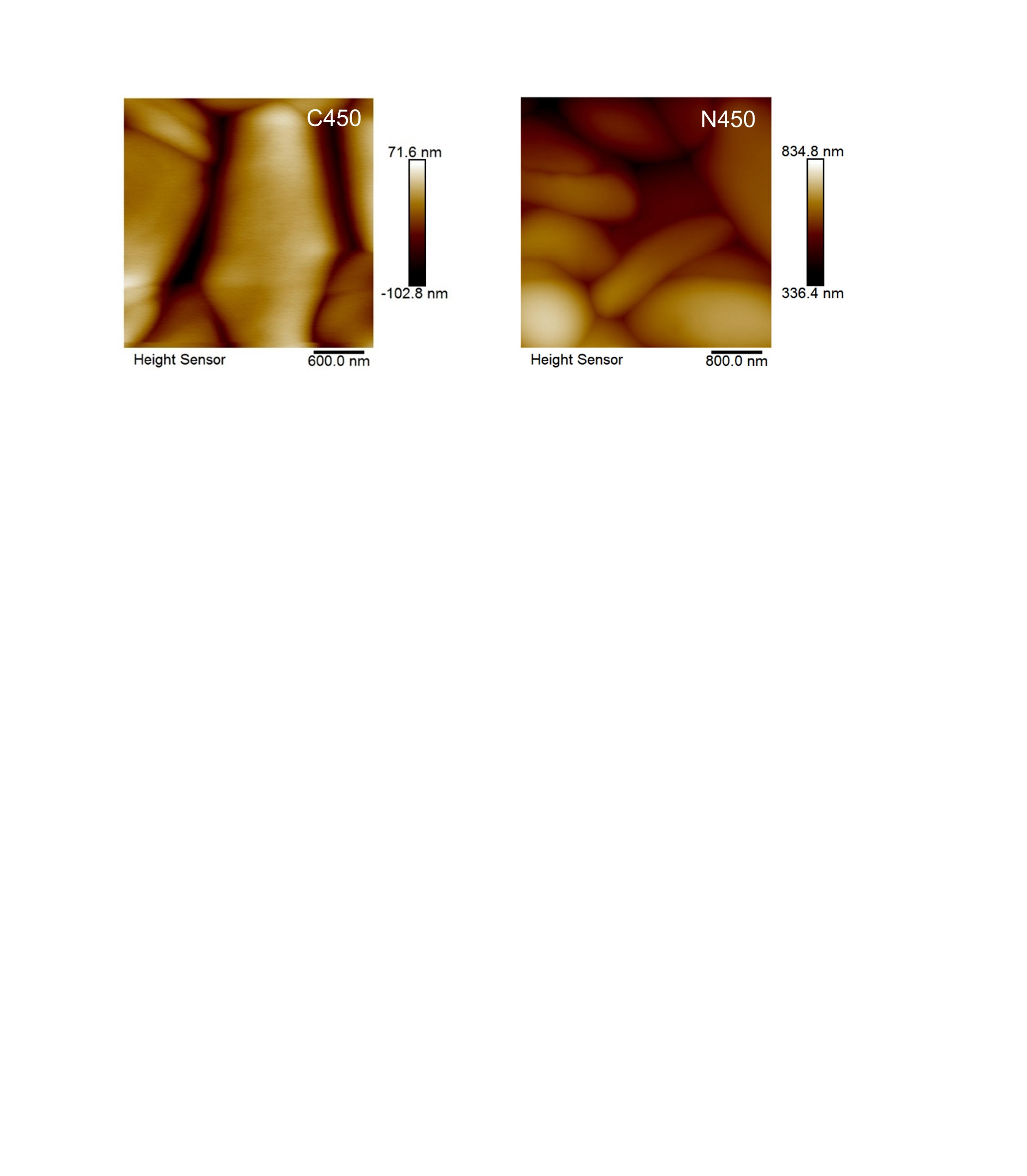


**Fig. S36** AFM image of C450 devices


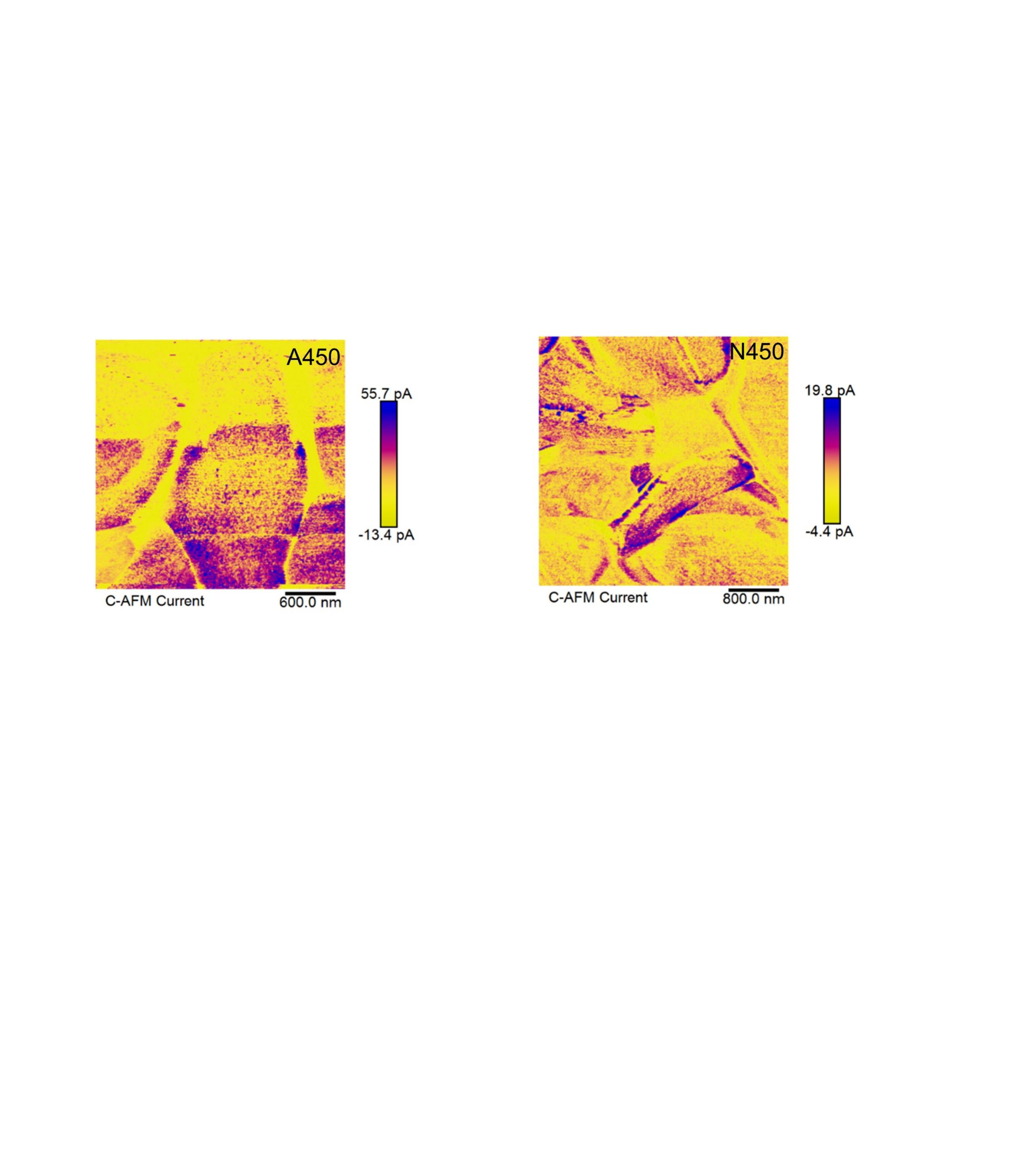


**Fig. S37** c-AFM image of N450 devices


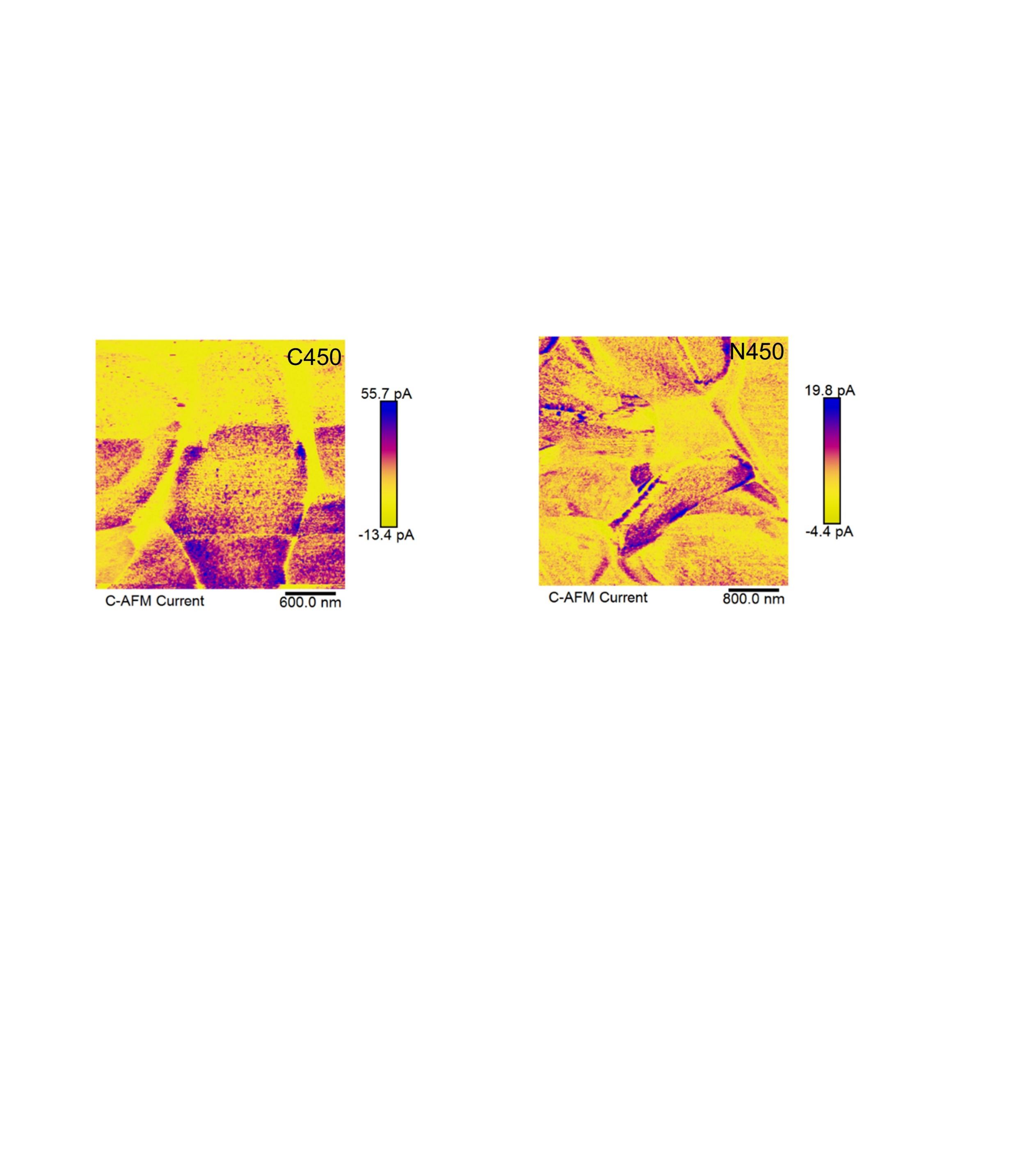


**Fig. S38** c-AFM image of C450 devices


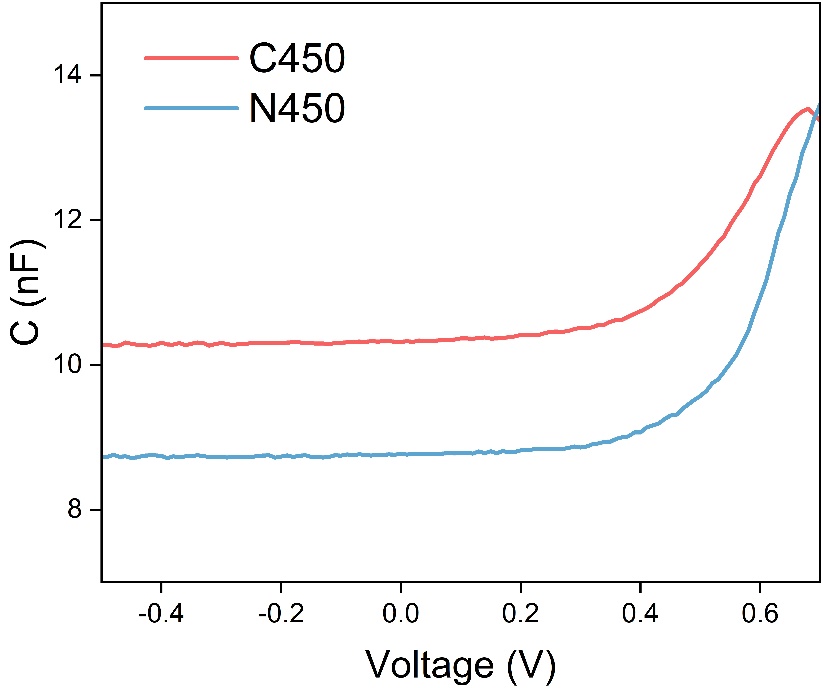


**Fig. S39** *C*-*V* curves of C450 and N450 devices


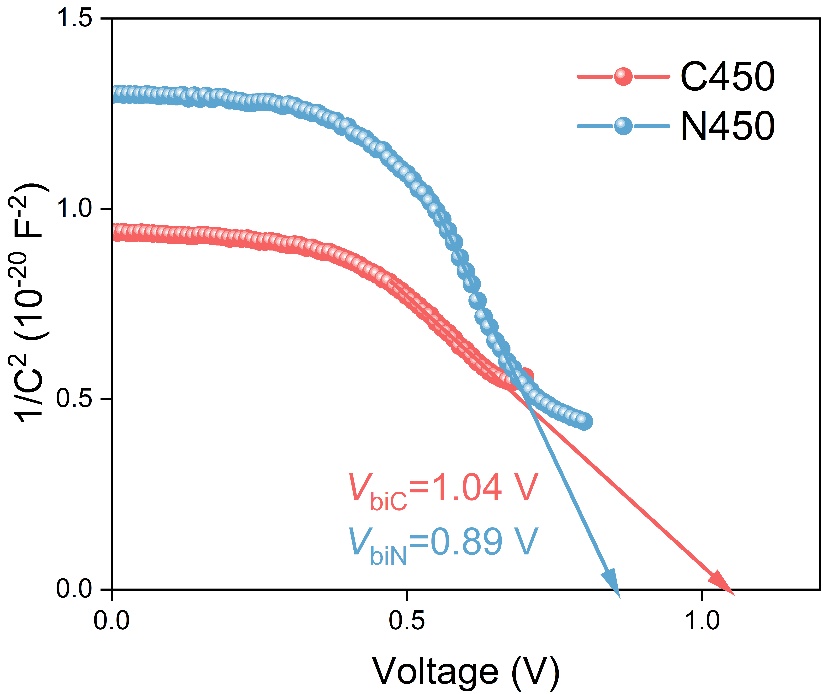


**Fig. S40** 1/*C*^2^-*V* curves with a frequency of 1 kHz for C450 and N450 devices


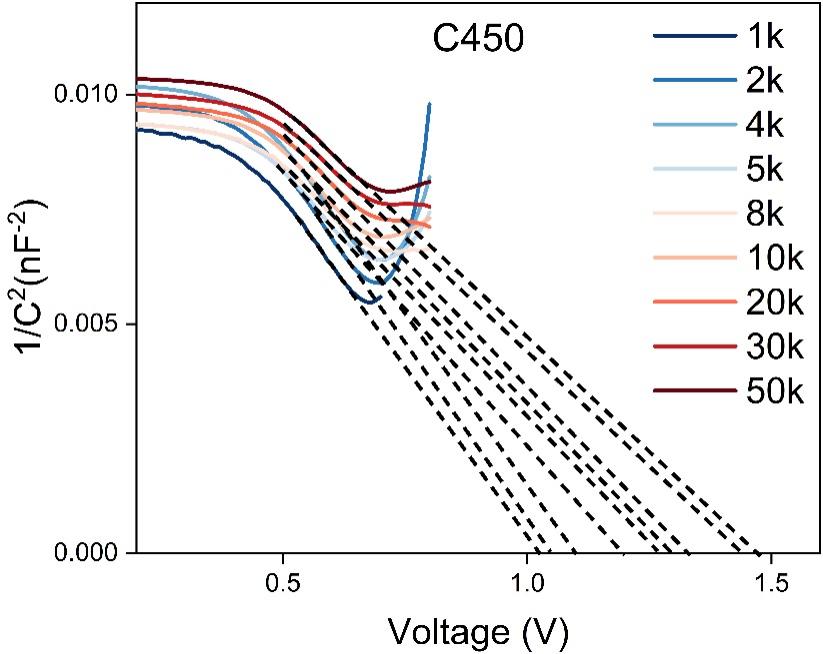


**Fig. S41** 1/C^2^-*V* curves of the C450 device at different frequencies


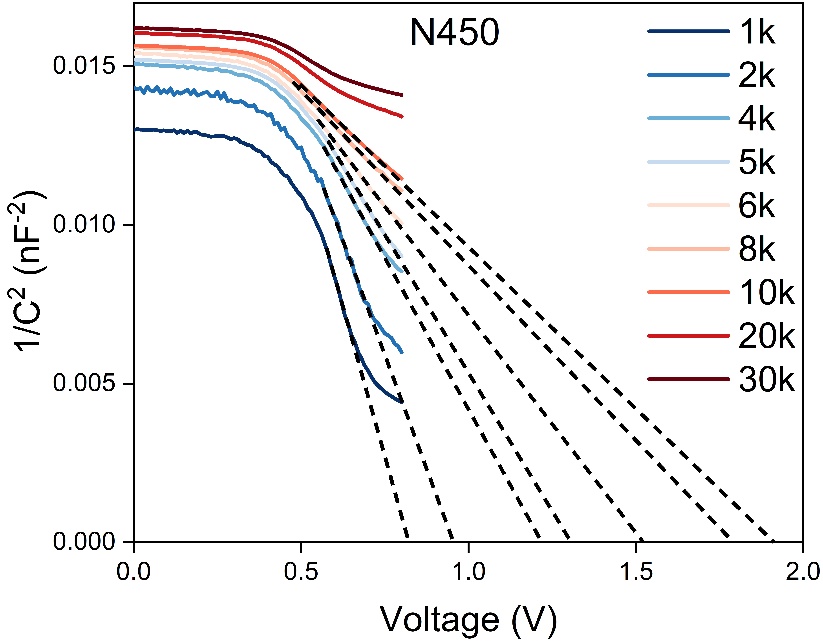


**Fig. S42** 1/C^2^-*V* curves of the N450 device at different frequencies


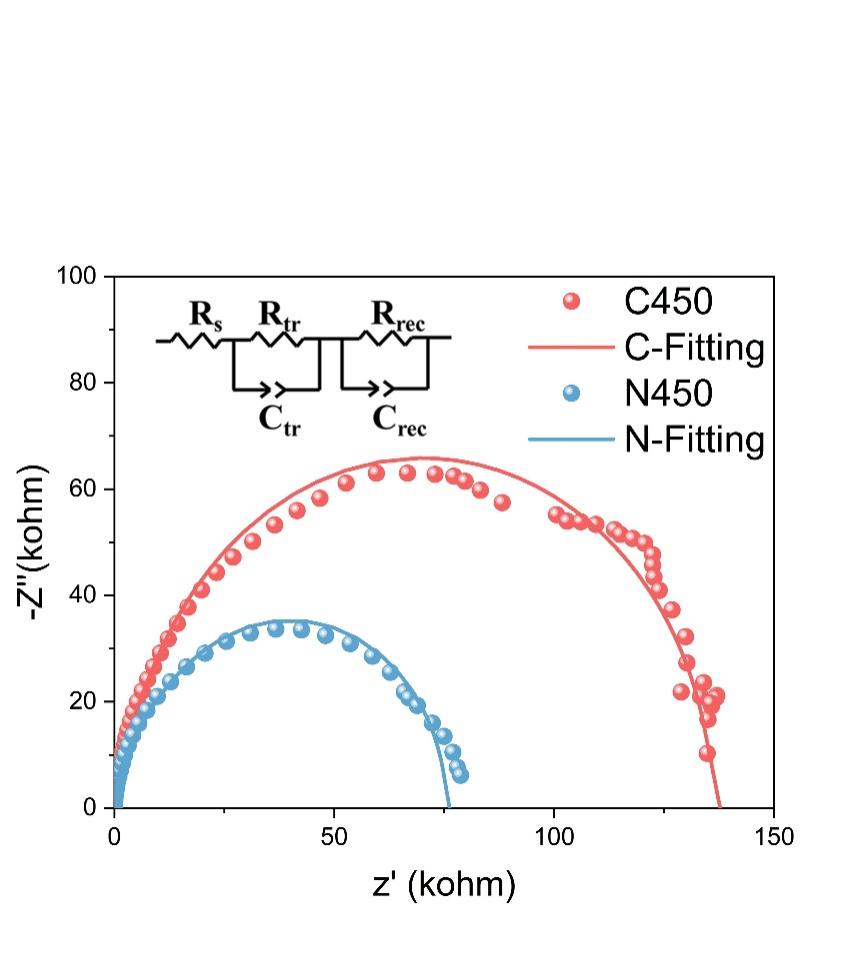


**Fig. S43** Nyquist plots of C450 and N450 devices

The equivalent circuit consists of a series resistance (*R*_s_), a charge conduction resistance (*R*_tr_), a recombination resistance (*R*_rec_), and the corresponding parallel capacitances *C*_tr_ and *C*_rec_. The fitting parameters are listed in **Table S4**. The *R*_s_ and *R*_tr_ of the C450 cell are 51 Ω and 5.2 kΩ, respectively, which are lower than those of the N450 cell (69 Ω and 7.1 kΩ), indicating that the C450 cell has less energy loss during the charge transfer process and a higher charge conductivity, consistent with the above c-AFM test results. *R*_rec_ is inversely proportional to the carrier recombination rate of the device. The *R*_rec_ of the C450 solar cell is 131 kΩ, significantly higher than that of the N450 cell (68.3 kΩ), indicating that the carrier recombination loss in the C450 device is smaller.

**
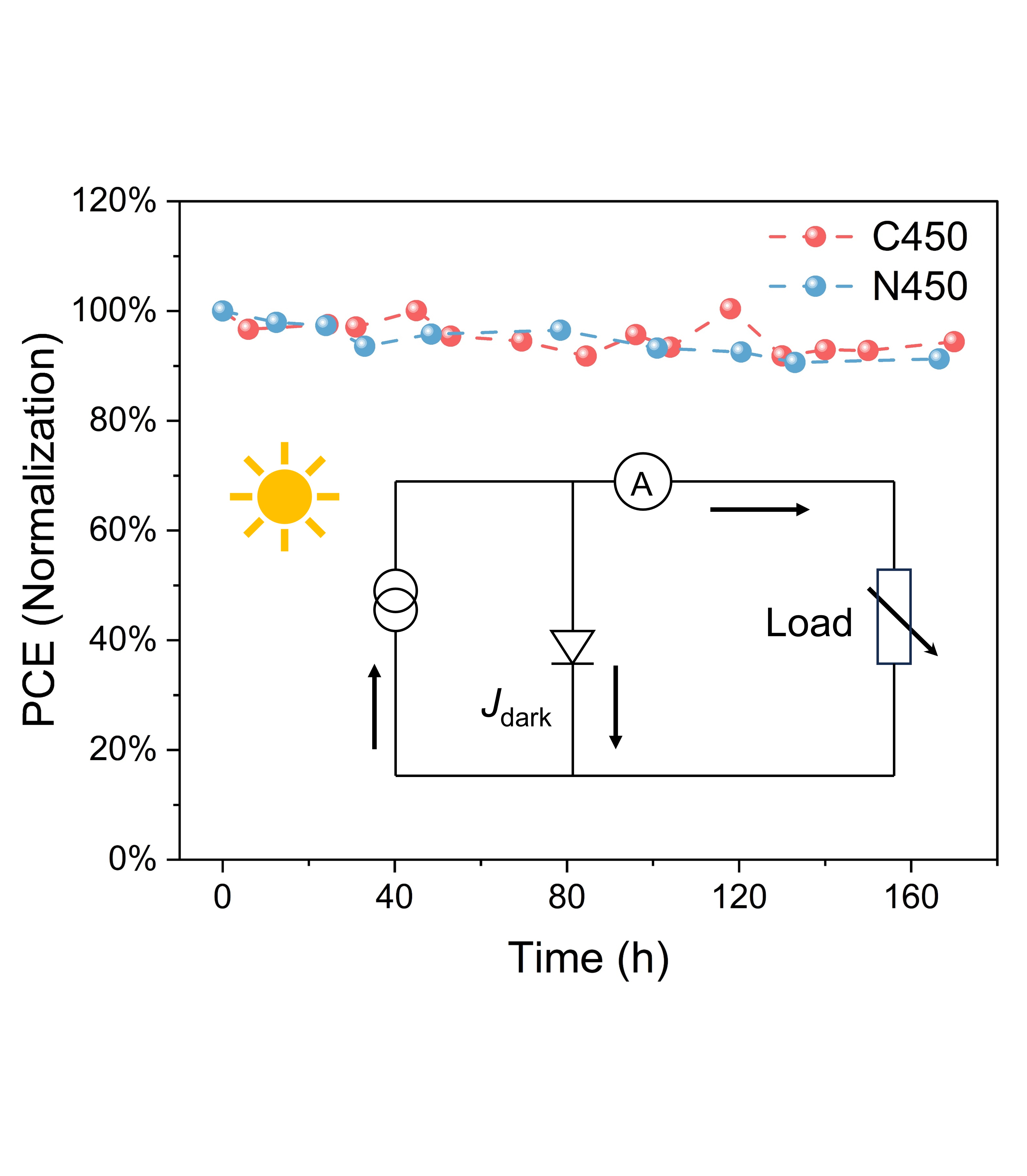
**

**Fig. S44** The performance of the devices in the stability test


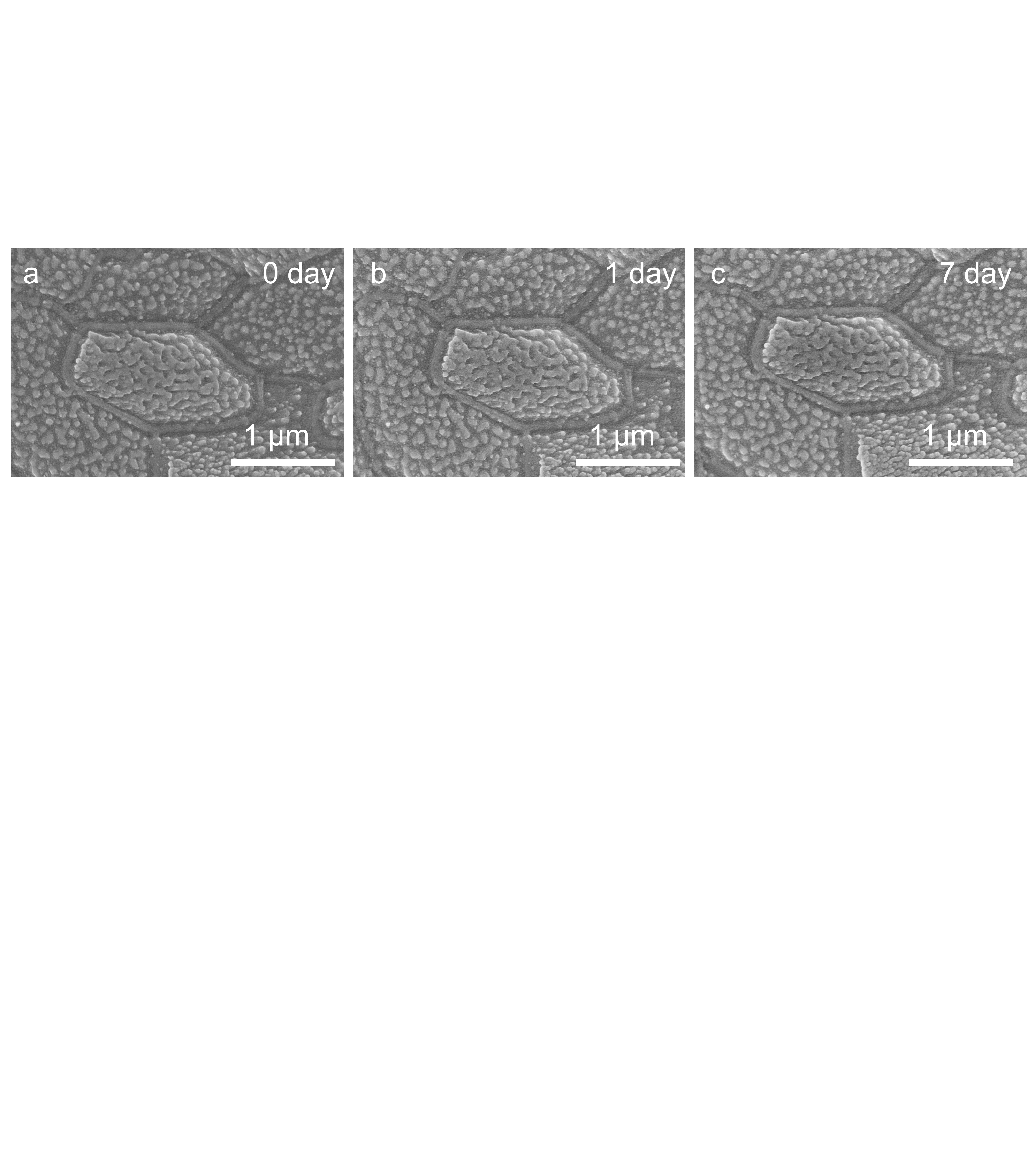


**Fig. S45** SEM images of C450 under stability tests of **a** 0 day, **b** 1 day, and **c** 7 day

**Table S1** Sample Naming Instructions

| Sample | Annealing Mode | Annealing Temperature (°C) | Opening Temperature (°C) |
| --- | --- | --- | --- |
| O290-O470 | open-air annealing | 290-470 | 290-470 |
| C290-C470 | confined-space annealing | 290-470 | 290-470 |
| U410-U470 | up-coverage annealing | 410-470 | 410-470 |
| D275-D450 | dynamic in-situ oxidation | 450 | 275-450 |
| N450 | nitrogen atmosphere annealing | 450 | 450 |

**Table S2** The average value of the solar cell statistics results

| Sample | *V*_oc_ (mV) | *J*_sc_ (mA/cm^2^) | FF (%) | PCE (%) |
| --- | --- | --- | --- | --- |
| O290 | 620 | 6.99 | 50.7 | 2.19 |
| O320 | 636 | 9.16 | 51.5 | 3.00 |
| O350 | 626 | 3.40 | 32.1 | 0.68 |
| C410 | 735 | 14.28 | 58.6 | 6.16 |
| C430 | 739 | 14.15 | 59.5 | 6.21 |
| C450 | 746 | 14.45 | 60.2 | 6.52 |
| C470 | 682 | 11.28 | 47.1 | 3.63 |

**Table S3** Value of charge-transport loss and nonradiative loss

| Sample | FF_SQ_ (%) | FF_max_ (%) | FF_Experiment_ (%) | Non-radiative loss (%) | Transport loss (%) |
| --- | --- | --- | --- | --- | --- |
| C450 | 91.24 | 76.97 | 62.7 | 14.27 | 14.27 |
| N450 | 91.06 | 75.08 | 51.6 | 15.98 | 23.48 |

**Table S4** Retrieved parameters of Nyquist plots of C450 and N450 devices

| Sample | *R*_s_ (ohm) | *R*_tr_ (kohm) | *C*_tr_ (nF) | *R*_rec_ (kohm) | *C*_rec_ (nF) |
| --- | --- | --- | --- | --- | --- |
| C450 | 51 | 5.2 | 32.7 | 131 | 12.7 |
| N350 | 69 | 7.1 | 20.5 | 68.3 | 12.4 |

**Table S5** Defect state, energy level (E_T_), cross-section (σ), defect density (N_T_), and (σN_T_) of the detected defects in C450 and N450 devices

| Sample | Trap type | E_T_ (eV) | σ (cm^2^) | N_T_ (cm^-3^) | σN_T_ (cm^-1^) | PCE (%) |
| --- | --- | --- | --- | --- | --- | --- |
| C450 | E1 | E_c_-0.200 | 3.02×10^-17^ | 1.07×10^14^ | 3.23×10^-3^ | 7.17 |
|  | E4 | E_c_-0.391 | 1.65×10^-16^ | 1.17×10^14^ | 1.93×10^-2^ |  |
|  | H1 | E_v_+0.603 | 1.05×10^-15^ | 3.44×10^14^ | 3.61×10^-1^ |  |
| N450 | E1 | E_c_-0.295 | 2.03×10^-16^ | 3.63×10^14^ | 7.37×10^-2^ | 5.11 |
|  | E2 | E_c_-0.326 | 1.23×10^-17^ | 2.95×10^14^ | 3.63×10^-3^ |  |
|  | E3 | E_c_-0.347 | 3.47×10^-18^ | 3.41×10^14^ | 1.18×10^-3^ |  |
|  | E4 | E_c_-0.564 | 5.09×10^-15^ | 2.99×10^14^ | 1.52 |  |
|  | H1 | E_v_+0.628 | 2.80×10^-16^ | 1.33×10^15^ | 3.72×10^-1^ |  |

**Supplementary References**

1. N. A. Lange, J. G. Speight, Lange’s Handbook of Chemistry, 16th edn. (McGraw-Hill, New York, 2005).
2. A. D. Le Claire, The theory of D_0_ in the Arrhenius equation for self-diffusion in cubic metals. Acta Metall. **1**, 438-447 (1953). <https://doi.org/10.1016/0001-6160(53)90127-8>
3. N. A. Shah, I. L. Animasaun, R. O. Ibraheem, H. A. Babatunde, N. Sandeep et al., Scrutinization of the effects of Grashof number on the flow of different fluids driven by convection over various surfaces. J. Mol. Liq. **249**, 980-990 (2018). <https://doi.org/10.1016/j.molliq.2017.11.042>
4. R. Tang, X. Wang, W. Lian, J. Huang, Q. Wei et al., Hydrothermal deposition of antimony selenosulfide thin films enables solar cells with 10% efficiency. Nat. Energy **5**, 587-595 (2020). <https://doi.org/10.1038/s41560-020-0652-3>
5. H. Li, J. Bao, J. Cai, Y. Xia, L. Liu et al., Solution‐Processed multivalent molybdenum oxide tailoring band alignment for efficient Sb_2_S_3_ solar cells. Small **21**, e07731 (2025). <https://doi.org/10.1002/smll.202507731>
6. X. Wang, R. Tang, C. Jiang, W. Lian, H. Ju et al., Manipulating the electrical properties of Sb_2_(S,Se)_3_ film for high‐efficiency solar cell. Adv. Energy Mater. **10**, 2002341 (2020). <https://doi.org/10.1002/aenm.202002341>
7. J. T. Heath, J. D. Cohen, W. N. Shafarman, Bulk and metastable defects in CuIn_1−x_Ga_x_Se_2_ thin films using drive-level capacitance profiling. J. Appl. Phys. **95**, 1000-1010 (2004). <https://doi.org/10.1063/1.1633982>
8. K. Gӧdel, B. Roose, A. Sadhanala, Y. Vaynzof, S. Pathak et al., Partial oxidation of the absorber layer reduces charge carrier recombination in antimony sulfide solar cells. Phys. Chem. Chem. Phys. **19**, 1425-1430 (2017). <https://doi.org/10.1039/c6cp07559b>
9. Pawar, R. Nandi, K. Neerugatti, I. Sharma, R. Yadav et al., Atomic-layer-deposited TiO_2_ and SnO_2_ coupled with CdS as double buffer layers for HTL-free Sb_2_S_3_ thin-film solar cells. Sol. Energy **246**, 141-151 (2022). <https://doi.org/10.1016/j.solener.2022.09.044>
10. N. Gunasekaran, D. Nagarajan, D. Nataraj, K. Prabakar. Modulation of energy band positions in Sb_2_S_3_ thin films for enhanced photovoltaic performance of FTO/TiO_2_/Sb_2_S_3_/P3HT/Au solar cell. Energy Technol. **13**, 2401475 (2025). <https://doi.org/10.1002/ente.202401475>
11. H. Li, J. Bao, J. Cai, Y. Xia, L. Liu et al., Solution-processed multivalent molybdenum oxide tailoring band alignment for efficient Sb_2_S_3_ solar cells, Small **21**, e07731 (2025). <https://doi.org/10.1002/smll.202507731>
12. C. Jiang, R. Tang, X. Wang, H. Ju, G. Chen et al., Alkali metals doping for high-performance planar heterojunction Sb_2_S_3_ solar cells. Sol. RRL **3**, 1800272 (2019). <https://doi.org/10.1002/solr.201800272>
13. X. Li, Y. Yang, L. Feng, Y. Yang, K. Hu et al., Tailoring presynthesized amorphous Sb_2_S_3_ particles enables highefficiency pure antimony sulfide solar cells. ACS Appl. Mater. Interfaces **17**, 16738-16746 (2025). <https://doi.org/10.1021/acsami.4c17684>
14. H. Ning, H. Guo, J. Zhang, X. Wang, X. Jia et al., Enhancing the efficiency of Sb_2_S_3_ solar cells using dual-functional potassium doping. Sol. Energy Mater. Sol. Cells **221**, 110816 (2021). <https://doi.org/10.1016/j.solmat.2020.110816>
15. J. Han, S. Wang, J. Yang, S. Guo, Q. Cao et al., Solution-processed Sb_2_S_3_ planar thin film solar cells with a conversion efficiency of 6.9% at an open circuit voltage of 0.7 V achieved via surface passivation by a SbCl_3_ interface layer. ACS Appl. Mater. Interfaces **12**, 4970-4979 (2020). <https://doi.org/10.1021/acsami.9b15148>
16. S. Chen, M. Li, Y. Zhu, X. Cai, F. Xiao et al., A codoping strategy for efficient planar heterojunction Sb_2_S_3_ solar cells. Adv. Energy Mater. **12**, 2202897 (2022). <https://doi.org/10.1002/aenm.202202897>
17. J. Zheng, C. Liu, L. Zhang, Y. Chen, F. Bao et al., Enhanced hydrothermal heterogeneous deposition with surfactant additives for efficient Sb_2_S_3_ solar cells. Chem. Eng. J. **446**, 136474 (2022). <https://doi.org/10.1016/j.cej.2022.136474>
18. S. Wang, Y. Zhao, B. Che, C. Li, X. Chen et al., A novel multi-sulfur source collaborative chemical bath deposition technology enables 8%-efficiency Sb_2_S_3_ planar solar cells. Adv. Mater. **34**, e2206242 (2022). <https://doi.org/10.1002/adma.202206242>
19. L. Zhu, R. Liu, Z. Wan, W. Cao, C. Dong et al., Parallel planar heterojunction strategy enables Sb_2_S_3_ solar cells with efficiency exceeding 8%. Angew. Chem. **135**, e202312951 (2023). <https://doi.org/10.1002/anie.202312951>
20. C. Wu, L. Zhang, B. Che, P. Xiao, J. Yang et al., The role of grain growth in controlling the crystal orientation of Sb_2_S_3_ films for efficient solar cells. J. Mater. Chem. A **11**, 8184-8191 (2023). <https://doi.org/10.1039/d3ta00678f>
21. Y. Huang, H. Gao, X. Peng, G. Wang, P. Xiao et al. A robust hydrothermal sulfuration strategy toward effective defect passivation enabling 6.92% efficiency Sb_2_S_3_ solar cells. Sol. RRL **7**, 2201115 (2023). <https://doi.org/10.1002/solr.202201115>
22. Y. Wang, Z. Wang, M. Fan, M. Li, L. Xie et al., An in situ polymerization-assisted grain growth strategy for efficient and stable Sb_2_S_3_ solar cells. ACS Appl. Energy Mater. **7**, 4252-4259 (2024). <https://doi.org/10.1021/acsaem.4c00646>
23. X. Liu, Z. Cai, L. Wan, P. Xiao, B. Che et al., Grain engineering of Sb_2_S_3_ thin films to enable efficient planar solar cells with high open-circuit voltage. Adv. Mater. **36**, 2305841 (2024). <https://doi.org/10.1002/adma.202305841>
24. Z. Chen, X. Chen, J. Zhou, B. Tang, Y. Li et al., Evaluating the film orientation and grain boundary of vacuum and solution-processed Sb_2_S_3_ films toward efficient solar cells. Energy Fuels **38**, 22536-22542 (2024). <https://doi.org/10.1021/acs.energyfuels.4c04739>
25. X. Chen, Y. Zhao, C. Li, X. Wang, P. Xiao et al., Interfacial engineering by self-assembled monolaver for high-performance Sb_2_S_3_ solar cells. Adv. Energy Mater. **14**, 2400441 (2024). <https://doi.org/10.1002/aenm.202400441>
26. H. Hussien, M. Krunks, N. Spalatu, A. Katerski, Z. Li-Kao et al., Interface engineering approach of in-air-processed Sb_2_S_3_ solar cells enabling 7.5% AM 1.5G device efficiency and an 18% indoor milestone performance. J. Mater. Chem. A **13**, 37215-37231 (2025). <https://doi.org/10.1039/d5ta05790f>
27. Y. Wang, D. Yang, M. Jin, Z. Wan, W. Cao et al., Full-dimensional penetration strategy with degradable PEAI enables 8.21% efficiency in bulk heterojunction Sb_2_S_3_ solar cells. Adv. Energy Mater. **15**, 2502805 (2025). <https://doi.org/10.1002/aenm.202502805>
28. Y. Wang, M. Jin, Z. Wan, C. Chen, W. Cao et al., PbSe-induced Sb_2_S_3_ crystallization and interface band optimization for high-efficiency bulk heterojunction Sb_2_S_3_ solar cells. Adv. Funct. Mater. **35**, 2420361 (2025). <https://doi.org/10.1002/adfm.202420361>
29. G. Shen, A. Ke, S. Chen, T. Ma, S. Ali et al., Strong chelating additive and modified electron transport layer for 8.26%-efficient Sb_2_S_3_ solar cells. Adv. Energy Mater. **15**, 2406051 (2025). <https://doi.org/10.1002/aenm.202406051>
30. C. Ying, Y. Xie, X. Zhu, Y. Zhang, X. Zhu et al., Low Sb-O content Sb_2_S_3_ thin films via a nonaqueous-based chemical bath deposition for efficient indoor Sb_2_S_3_ solar cells. ACS Appl. Energy Mater. **8**, 11643-11651 (2025). <https://doi.org/10.1021/acsaem.5c01820>
31. G. Wang, L. Feng, Y. Huang, J. Yang, H. Wang et al., Boosting efficiency of hydrothermally grown Sb_2_S_3_ solar cells via rational sulfur engineering. Adv. Funct. Mater. **35**, e18624 (2025). <https://doi.org/10.1002/adfm.202518624>
32. Y. Li, Y. Yin, R. Deng, L. Feng, Z. Yu et al., Efficient Sb_2_S_3_ solar cells via thioacetamide-based hydrothermal deposition. Chem. Commun. **61**, 17013-17016 (2025). <https://doi.org/10.1039/d5cc05163k>
33. X. Mao, F. Shi, W. Tang, H. Wu, T. Cheng et al., Surfactant sodium dodecylbenzenesulphonate assisted growth of large-grain Sb_2_S_3_ films with reduced defect for highly efficient solar cells. Sol. RRL **9**, e2500473 (2025). <https://doi.org/10.1002/solr.202500473>
34. L. Yao, L. Lin, Z. Huang, Y. Mao, H. Li et al. A liquid medium annealing strategy for highly [041]/[141]-oriented planar antimony sulfide solar cells with 7.23% efficiency. Nano Energy **106**, 108064 (2023). <https://doi.org/10.1016/j.nanoen.2022.108064>
35. M. Rui, X. Lu, Y. Li, S. Liu, X. Jin et al., Enhanced performance of Sb_2_S_3_ solar cells via coevaporated MnS-PbS composite hole transport layers. ACS Appl. Energy Mater. **8**, 11584-11593 (2025). <https://doi.org/10.1021/acsaem.5c01707>
36. Y. Li, X. Lu, S. Liu, M. Rui, S. Wang, Sb_2_S_3_/MnS interface optimization for improved all-inorganic Sb_2_S_3_ solar cell performance based on NH4F impregnation. Sol. Energy Mater. Sol. Cells **292**, 113805 (2025). <https://doi.org/10.1016/j.solmat.2025.113805>
